# Copper-Catalyzed Synthesis of Axially Chiral Biaryls with Diaryliodonium Salts as Arylation Reagents

**DOI:** 10.3390/molecules26113223

**Published:** 2021-05-27

**Authors:** Ji-Wei Zhang, Shao-Hua Xiang, Shaoyu Li, Bin Tan

**Affiliations:** 1 Shenzhen Key Laboratory of Small Molecule Drug Discovery and Synthesis, Department of Chemistry, Southern University of Science and Technology, Shenzhen 518055, China; 11649015@mail.sustech.edu.cn (J.-W.Z.); lisy@sustech.edu.cn (S.L.); 2 Academy for Advanced Interdisciplinary Studies, Southern University of Science and Technology, Shenzhen 518055, China

**Keywords:** NOBIN, BINAM, arylation, sigmatropic rearrangement, copper-catalysis

## Abstract

NOBIN and BINAM derivatives harboring biaryl frameworks are recognized as a class of important atropisomers with versatile applications. Here, we present an efficient synthetic route to access such compounds through copper-catalyzed domino arylation of *N*-arylhydroxylamines or *N*-arylhydrazines with diaryliodonium salts and [3,3]-sigmatropic rearrangement. This reaction features mild conditions, good substrate compatibility, and excellent efficiency. The practicality of this protocol was further extended by the synthesis of biaryl amino alcohols.

## 1. Introduction

**Figure 1:**
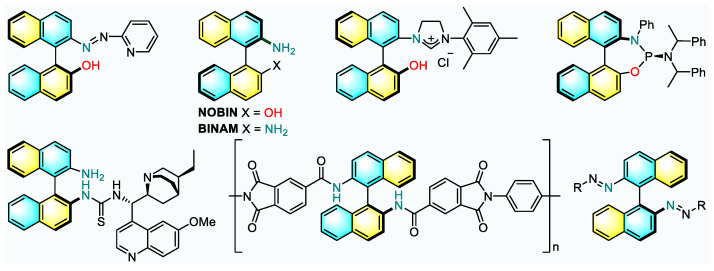
Representative molecules deriving from NOBIN or BINAM.

Axially chiral biaryl frameworks constitute the core structure of a wide range of natural products and biologically active molecules. They are also found widespread applications as chiral catalysts and ligands. In the asymmetric catalysis field, 2-amino-2′-hydroxy-1,1′-binaphthyl (NOBIN) and 1,1′-binaphthyl-2,2′-diamine (BINAM) are among the most frequently utilized structures [1,2,3,4]. Now, NOBIN and BINAM derivatives have been involved in metal catalysis [5,6,7], organocatalysis [8,9], photocatalysis [10], and even heterogeneous catalysis [11] for effective chirality induction. Meanwhile, the significance of such backbones is further illustrated by their prevalence in functional materials [12,13] (Figure 1). Accordingly, the construction of NOBIN and BINAM scaffolds has attracted extensive attentions from the synthetic research community.

For NOBIN and its derivatives, they could be accessed from other binaphthyl compounds such as BINOL or BINAM [14,15,16]. However, in these synthetic processes, excess noble metal reagents, harsh conditions, or expensive reagents were commonly required to achieve satisfactory efficiency [17]. The oxidative cross-coupling of 2-naphthol and 2-naphthylamine catalyzed by transition metal represents the most effective and direct method to establish the aryl–aryl axis. Kočovský and coworkers pioneered the strategy of oxidative cross-coupling using copper amine complexes as oxidants [18,19,20,21]. Subsequently, Ding [22] and Carreira [23] provided a series of improved approaches to enhance the synthetic efficiency and applicability in large-scale preparations and inhibit inseparable homo-coupling by-products. Recently, Tu and coworkers successfully constructed enantioenriched 3,3′-disubstituted NOBINs by aerobic oxidative cross-coupling utilizing a novel Cu/SPDO catalytic system [24]. The redox potential difference between two coupling partners ensured good chemoselectivity and chemical yield during the coupling process. Our group developed an efficient coupling approach for the synthesis of NOBINs via a palladium-catalyzed highly site-selective C-H arylation reaction of *N*-Boc-2-naphthylamines with diazoquinones under mild conditions [25].

As an effective Ar-Ar bond formation method, [3,3]-sigmatropic rearrangement reaction was emerged as an attractive alternative [26,27,28,29,30,31,32]. In this context, Gao [33] and our group [34] independently developed a transition metal-free approach to generate NOBIN derivatives following a domino arylation of naphthylhydroxylamines with diaryliodonium salts and [3,3]-sigmatropic rearrangement. It should be mentioned that moderate yields were normally obtained for Gao’s conditions, while the mixed solvent of dichloromethane and trifluoroethanol was required to improve reaction results.

**Figure 2:**
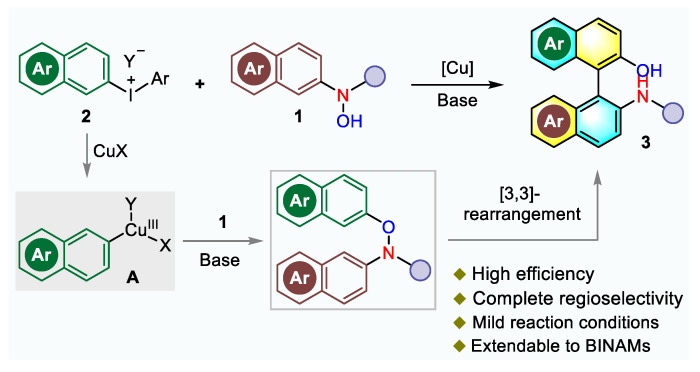
Our strategies for synthesis of NOBIN derivatives via copper-catalyzed domino arylation and rearrangement.

The progress in synthesis of biaryls employing diaryliodonium salts as aryl cation equivalents has made this class of bench-stable, nontoxic, and readily available reagents attract attention [35,36,37,38,39]. Moreover, copper catalyst can be oxidized in the presence of diaryliodonium salts to form a highly electrophilic aryl-Cu(III) intermediate and a range of latent nucleophiles undergo arylation reactions to form synthetically versatile products [39,40]. In view of the advantages and reliability with diaryliodonium salts, Cu-catalyzed arylation of *N*-arylhydroxylamine or *N*-arylhydrazine can effectively generate transient diaryl groups linked by heteroatoms, which can easily undergo rearrangement reactions. Motivated by our continuous research interests in constructing biaryl frameworks [41,42,43,44], we turned our attention to construct the NOBIN and BINAM derivatives via copper-catalyzed *N*-/*O*-arylation with diaryliodonium salts and subsequent [3,3]-sigmatropic rearrangement under mild conditions (Figure 2). The properties of biaryls are affected by the steric hindrance and electronic effect of substituents, which will bring new opportunities in application and expansion. The arylation-rearrangement sequence that allows new library synthesis is still desired.

## 2. Results and Discussion

### 2.1. Optimization of Reaction Conditions

Upon exploring some reaction conditions through variation of the copper catalysts, solvents and bases (see Supplementary Materials for details), the following protocol was identified to be optimal: reaction of **1a** and **2a** with the molar ratio of 1.0:1.2 by using Cu(TFA)_2_ (10 mol%) as catalyst in dichloromethane (DCM) at room temperature, **3a** was obtained almost quantitatively (Table 1, entry 1). When evaluating different solvents, DCM outcompeted others to form a desired product (Table 1, entries 2–5). As far as the catalyst is concerned, other screened Cu(II) or Cu(I) bearing different anions also gave **3a** in high yield under mild conditions (Table 1, entries 6–9). Finally, other carbonate salts, NaOH, *t*BuOK, and amine are inferior to Na_2_CO_3_ in facilitating the arylation process (Table 1, entries 10–14).

### 2.2. Substrate Scope

**Figure 3:**
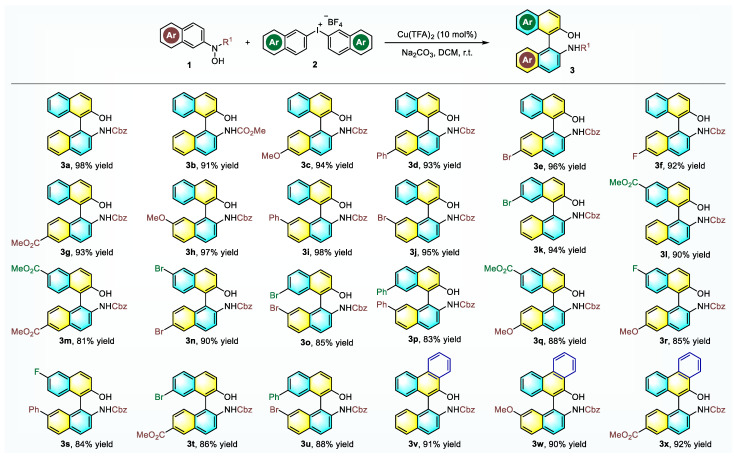
Substrate scope of *N*-naphthylhydroxylamines and diaryliodonium salts. All reactions were performed with Cu(TFA)_2_ (10 mol%), **1a** (0.20 mmol), **2a** (0.24 mmol) and base (0.26 mmol) in DCM (4.0 mL) at room temperature; Isolated yields were provided.

With the optimized conditions in hand, the generality of this transformation was then explored with respect to *N*-naphthylhydroxylamines **1** and diaryliodonium salts **2**. As shown in Figure 3, all the investigated substrates were completely transformed and furnished the respective product in generally high efficiency with a yield of up to 98%. In detail, the replacement of the Cbz-protecting group with a methyl formate gave the desired product in 91% yield. Different substituents on the aromatic ring including halides, methyl ester, phenyl, and methoxy were all compatible for this set of reaction conditions, and meanwhile, the substitution patterns and electronic properties of substituents exerted a limited influence on the reaction outcome. Further evaluations revealed that all the tested *N*-naphthylhydroxylamines **1** and diaryliodonium salts **2** with varied substitutions could undergo effective combination to give multi-substituted NOBINs **3m**–**x** in 81–92% yield. In addition, diaryliodonium salt with an extended fused ring system proved to be an applicable arylation reagent and produced the corresponding NOBINs in about 90% yield (products **3v**–**x**). It should be mentioned that the Br atom, which could act as an effective handle for further transformation, survived during this process.

**Figure 4:**
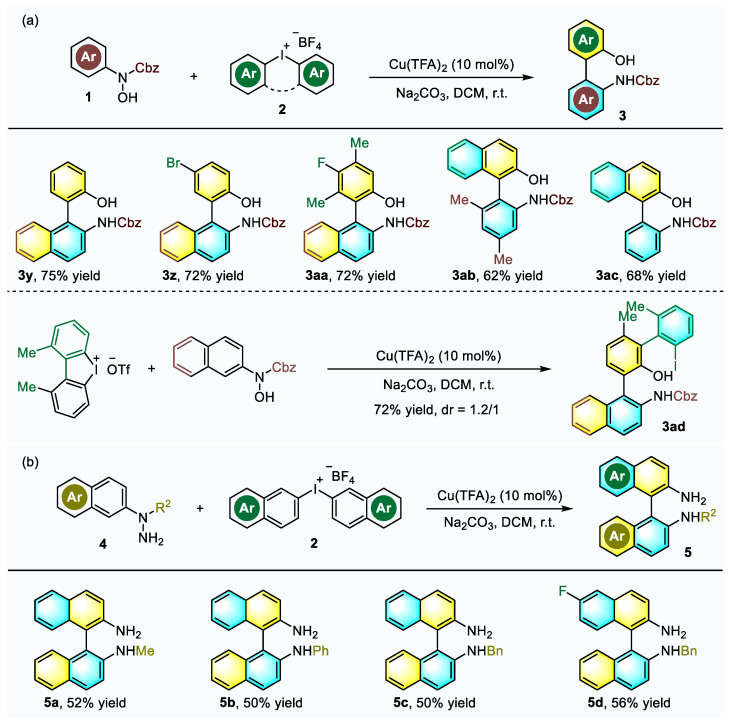
(**a**) Synthesis of biaryl amino alcohols under standard conditions; (**b**) Synthesis of BINAM derivatives under standard conditions.

**Figure 5:**
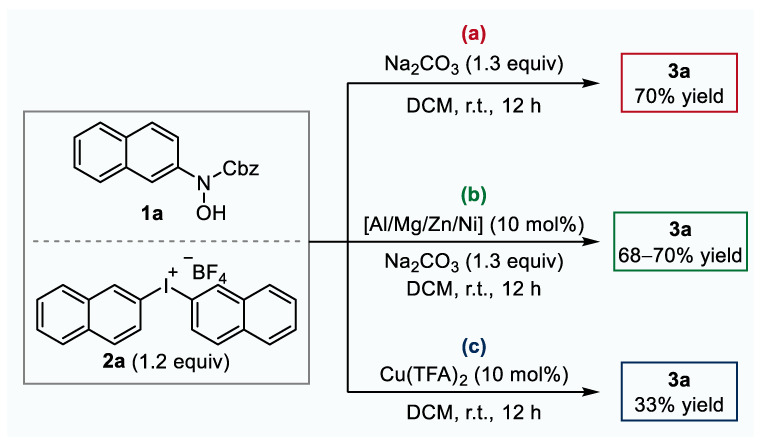
Control experiments: (**a**) transition metal-free; (**b**) aluminum(III), magnesium(II), zinc(II), or nickel(II) trifluoromethanesulfonate instead of copper(II) trifluoroacetate; (**c**) base free.

Subsequently, *N*-phenylhydroxyamines or diphenyliodonium salts were evaluated for this reaction to synthesize the biaryl amino alcohols. A series of structurally diverse compounds (Figure 4a, **3y**–**ac**) were obtained in good yields under the standard conditions. Cyclic diaryliodonium salt was verified to be a suitable arylation reagent, and the expected diaxial product was obtained in 72% yield as a pair of diastereomers with a ratio of 1.2:1 (Figure 4a, **3ad**). The successful establishment of a highly efficient domino approach to construct NOBINs inspired us to explore the feasibility in constructing BINAMs, which is another type of privileged biaryl atropisomers, to further extend the applicability and flexibility of the developed method. Pleasingly, when *N*-naphthylhydrazines **4** were utilized, the reactions with diaryliodonium salts **2** underwent smoothly to give BINAMs in moderate yield (Figure 4b, **5a**–**d**).

### 2.3. Control Experiments and Plausible Mechanism

Under transition metal-free conditions, the substrates could be completely converted, and the desired product **3a** was obtained in 70% yield (Figure 5a), along with several by-products. The use of Cu(TFA)_2_ not only improved the yield significantly but also shortened the reaction time, indicating that copper salt had an obvious catalytic effect on this type of reactions. Moreover, other examined Lewis acids such as the triflate of aluminum, magnesium, zinc, or nickel brought about a negligible effect on the reaction outcome (Figure 5b). In addition, a stoichiometric base was necessary for this reaction. When sodium carbonate was removed from the standard conditions, the target product **3a** could only be obtained in 33% yield (Figure 5c).

**Figure 6:**
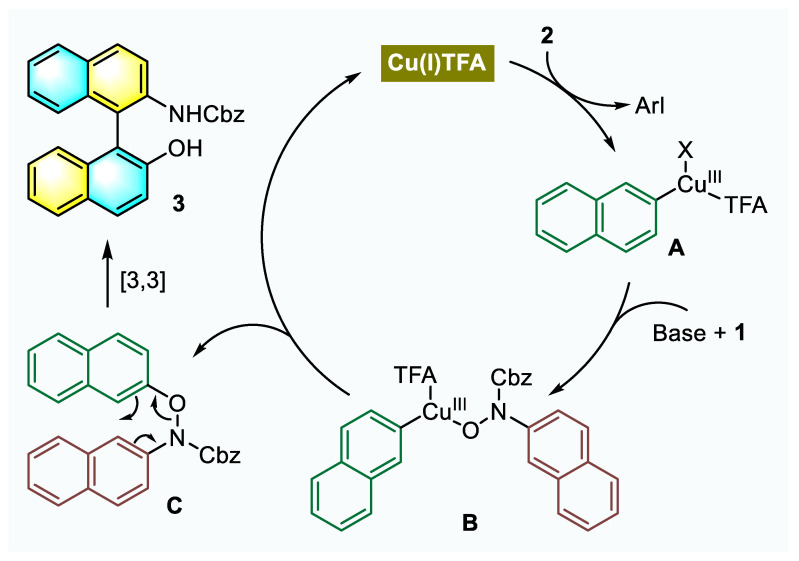
Proposed reaction pathway.

According to the results of the control experiments and previous reports on copper-catalyzed arylation reactions with diaryliodonium salts [36,39,45,46,47,48], a plausible reaction pathway involving 2-naphthyl-Cu(III) species was proposed, as shown in Figure 6. At first, the Cu(I) salt initially formed by Cu(II) disproportionation [49,50,51] undergoes oxidative addition into the Ar-I(III) bond to form the highly electrophilic aryl-Cu(III) intermediate **A**. Then, the complexation or nucleophilic substitution of aryl-Cu(III) species **A** with *N*-arylhydroxylamine **1** produces intermediate **B** under basic conditions. Upon reductive elimination, *N*,*O*-dinaphthylhydroxylamine **C** is generated, and active Cu(I) catalyst is released to continue the catalytic cycle. Next, the [3,3]-sigmatropic rearrangement step and subsequent rearomatization proceed rapidly to afford the product NOBIN **3**. As a transient precursor, **C** is quite difficult to be isolated from the reaction system, indicating a strong driving force for the following rearrangement.

## 3. Materials and Methods

Reagents were purchased at the highest commercial quality and used without further purification, unless otherwise stated. Cu(TFA)_2_ was purchased from Energy Chemical (Shanghai, China); Na_2_CO_3_ was purchased from Aladdin (Shanghai, China); Dichloromethane was purchased from TiTan (Shanghai, China). Analytical thin layer chromatography (TLC) was performed on precoated silica gel 60 F254 plates (Qingdao, China). Flash column chromatography was performed using Tsingdao silica gel (60, particle size 0.040–0.063 mm; Qingdao, China). Visualization on TLC was achieved by use of UV light (254 nm). NMR spectra were recorded on a Bruker DPX 400 spectrometer (Bruker BioSpin GmbH, Rheinstetten, Germany) at 400 MHz for ^1^H-NMR, 100 MHz for ^13^C-NMR and 376 MHz for ^19^F-NMR in CDCl_3_ or Acetone-*d*_6_ with tetramethylsilane (TMS) as internal standard. Chemical shifts are reported in ppm and coupling constants are given in Hz. Data for ^1^H-NMR are recorded as follows: chemical shift (ppm), multiplicity (s, singlet; d, doublet; t, triplet; m, multiplet), coupling constant (Hz), integration. Data for ^13^C-NMR are reported in terms of chemical shift (δ, ppm). High resolution mass spectra (HRMS) were recorded on a LC-TOF spectrometer (Thermo Fisher Scientific, Waltham, MA, USA).

General procedures for synthesis of NOBIN and BINAM derivatives: **1** or **4** (0.20 mmol), **2** (0.24 mmol), Na_2_CO_3_ (27.6 mg, 0.26 mmol), and Cu(TFA)_2_ (5.8 mg, 10 mol%) were added to a bottle with a magnetic stirring bar. DCM (4.0 mL) was added, and the reaction mixture was stirred at room temperature until **1** or **4** was completely consumed (monitored by TLC). After the solvent evaporated, the residue was purified by flash chromatography eluted with DCM to afford the corresponding product **3** or **5**.

Benzyl-(2′-hydroxy-[1,1′-binaphthalen]-2-yl)carbamate (**3a**) White solid. Yield: 98%. ^1^H-NMR (400 MHz, CDCl_3_) δ 8.56 (d, *J* = 9.6 Hz, 1H), 8.08 (d, *J* = 9.2 Hz, 1H), 8.00 (d, *J* = 9.2 Hz, 1H), 7.94 (t, *J* = 7.2 Hz, 2H), 7.47–7.27 (m, 10H), 7.15 (d, *J* = 8.8 Hz, 1H), 7.05 (d, *J* = 8.8 Hz, 1H), 6.54 (s, 1H), 5.21 (s, 1H), 5.07 (s, 2H). ^13^C-NMR (100 MHz, CDCl_3_) δ 153.6, 152.0, 135.9, 135.7, 133.2, 132.9, 131.3, 130.8, 130.4, 129.4, 128.6, 128.5, 128.4, 128.3, 128.3, 127.5, 127.4, 125.3, 125.1, 124.1, 124.0, 119.7, 117.9, 116.7, 112.7, 67.1. HRMS (ESI) calcd for [M + H] C_28_H_22_NO_3_, *m*/*z*: 420.1594, found: 420.1595.

Methyl-(2′-hydroxy-[1,1′-binaphthalen]-2-yl)carbamate (**3b**) Yellowish solid. Yield: 91%. ^1^H-NMR (400 MHz, CDCl_3_) δ 8.37 (d, *J* = 9.2 Hz, 1H), 7.89 (d, *J* = 8.8 Hz, 1H), 7.84 (d, *J* = 9.2 Hz, 1H), 7.77 (d, *J* = 8.0 Hz, 2H), 7.30–7.22 (m, 3H), 7.16–7.12 (m, 2H), 7.00 (d, *J* = 8.4 Hz, 1H), 6.89 (d, *J* = 8.0 Hz, 1H), 6.34 (s, 1H), 5.30 (s, 1H), 3.39 (s, 3H). ^13^C-NMR (100 MHz, CDCl_3_) δ 154.0, 152.1, 135.9, 133.2, 133.0, 131.2, 130.7, 130.3, 129.4, 128.4, 128.3, 127.5, 127.3, 125.1, 125.1, 124.1, 124.0, 119.3, 118.0, 116.5, 112.7, 52.3. HRMS (ESI) calcd for [M + H] C_22_H_18_NO_3_, *m*/*z*: 344.1281, found: 344.1281.

Benzyl-(2′-hydroxy-6-methoxy-[1,1′-binaphthalen]-2-yl)carbamate (**3c**) White solid. Yield: 94%. ^1^H-NMR (400 MHz, CDCl_3_) δ 8.45 (d, *J* = 8.8 Hz, 1H), 7.98 (d, *J* = 8.8 Hz, 1H), 7.96 (d, *J* = 9.6 Hz, 1H), 7.92 (dd, *J* = 8.4, 1.2 Hz, 1H), 7.42–7.26 (m, 8H), 7.24 (d, *J* = 2.4 Hz, 1H), 7.08 (d, *J* = 9.2 Hz, 1H), 7.06 (d, *J* = 8.4 Hz, 1H), 6.99 (dd, *J* = 9.2, 2.4 Hz, 1H), 6.48 (s, 1H), 5.45 (s, 1H), 5.07 (d, *J* = 12.4 Hz, 1H), 5.03 (d, *J* = 12.4 Hz, 1H), 3.92 (s, 3H). ^13^C-NMR (100 MHz, CDCl_3_) δ 157.3, 153.8, 152.0, 135.8, 133.8, 133.3, 132.1, 131.2, 129.4, 129.0, 128.6, 128.4, 128.3, 128.3, 128.3, 127.4, 126.8, 124.2, 123.9, 120.8, 119.9, 118.0, 117.9, 113.0, 106.5, 67.1, 55.4. HRMS (ESI) calcd for [M + H] C_29_H_24_NO_4_, *m*/*z*: 450.1700, found: 450.1697.

Benzyl-(2′-hydroxy-6-phenyl-[1,1′-binaphthalen]-2-yl)carbamate (**3d**) Yellowish solid. Yield: 93%. ^1^H-NMR (400 MHz, CDCl_3_) δ 8.48 (d, *J* = 9.2 Hz, 1H), 8.05 (d, *J* = 2.0 Hz, 1H), 8.02 (d, *J* = 9.2 Hz, 1H), 7.92 (d, *J* = 8.8 Hz, 1H), 7.86 (d, *J* = 7.6 Hz, 1H), 7.61 (dd, *J* = 7.6, 2.4 Hz, 2H), 7.49 (dd, *J* = 8.8, 2.0 Hz, 1H), 7.41 (t, *J* = 7.6 Hz, 2H), 7.35–7.30 (m, 3H), 7.27–7.21 (m, 4H), 7.19–7.14 (m, 3H), 7.02 (d, *J* = 8.4 Hz, 1H), 6.52 (s, 1H), 5.46 (s, 1H), 4.98 (d, *J* = 12.4 Hz, 1H), 4.93 (d, *J* = 12.4 Hz, 1H). ^13^C-NMR (100 MHz, CDCl_3_) δ 153.7, 152.2, 140.7, 138.0, 135.9, 135.7, 133.3, 132.2, 131.3, 131.1, 130.6, 129.5, 129.0, 128.6, 128.5, 128.4, 128.3, 127.5, 127.5, 127.3, 127.0, 126.1, 125.8, 124.2, 124.1, 120.2, 118.1, 117.1, 112.7, 67.2. HRMS (ESI) calcd for [M + H] C_34_H_26_NO_3_, *m*/*z*: 496.1907, found: 496.1909.

Benzyl-(6-bromo-2′-hydroxy-[1,1′-binaphthalen]-2-yl)carbamate (**3e**) Yellowish solid. Yield: 96%. ^1^H-NMR (400 MHz, CDCl_3_) δ 8.54 (d, *J* = 9.2 Hz, 1H), 8.09 (d, *J* = 2.0 Hz, 1H), 7.99 (d, *J* = 9.2 Hz, 1H), 7.94 (d, *J* = 8.8 Hz, 1H), 7.94 (d, *J* = 8.0 Hz, 1H), 7.43–7.25 (m, 9H), 7.02 (d, *J* = 9.2 Hz, 1H), 7.01 (d, *J* = 8.4 Hz, 1H), 6.57 (s, 1H), 5.60 (s, 1H), 5.05 (d, *J* = 12.0 Hz, 1H), 5.01 (d, *J* = 12.0 Hz, 1H). ^13^C-NMR (100 MHz, CDCl_3_) δ 153.6, 152.1, 136.1, 135.6, 133.2, 131.9, 131.6, 131.5, 130.6, 130.2, 129.4, 129.2, 128.6, 128.5, 128.4, 128.3, 127.6, 127.1, 124.1, 124.0, 120.9, 119.3, 118.1, 117.6, 112.2, 67.3. HRMS (ESI) calcd for [M + H] C_28_H_21_BrNO_3_, *m*/*z*: 498.0700, found: 498.0700.

Benzyl-(6-fluoro-2′-hydroxy-[1,1′-binaphthalen]-2-yl)carbamate (**3f**) White solid. Yield: 92%. ^1^H-NMR (400 MHz, CDCl_3_) δ 8.44 (d, *J* = 9.2 Hz, 1H), 7.91 (dd, *J* = 9.2, 2.8 Hz, 2H), 7.85 (d, *J* = 8.0 Hz, 1H), 7.47 (dd, *J* = 9.2, 2.8 Hz, 1H), 7.35–7.18 (m, 8H), 7.07 (dd, *J* = 9.3, 5.6 Hz, 1H), 7.00 (td, *J* = 8.8, 2.8 Hz, 1H), 6.94 (d, *J* = 8.4 Hz, 1H), 6.45 (s, 1H), 5.46 (s, 1H), 4.99 (d, *J* = 12.4 Hz, 1H), 4.94 (d, *J* = 12.4 Hz, 1H). ^13^C-NMR (100 MHz, CDCl_3_) δ 160.3 (d, *J* = 245.0 Hz), 153.8, 152.1, 135.7, 135.1 (d, *J* = 2.0 Hz), 133.2, 131.6 (d, *J* = 9.0 Hz), 131.4, 130.0, 129.4, 129.4, 129.4, 128.6, 128.5, 128.4, 128.3, 127.8 (d, *J* = 9.0 Hz), 127.6, 124.0 (d, *J* = 6.0 Hz), 121.2, 118.1, 117.9, 117.5 (d, *J* = 25.0 Hz) 112.5, 111.4 (d, *J* = 21.0 Hz), 67.2. ^19^F-NMR (376 MHz, CDCl_3_) δ −115.88. HRMS (ESI) calcd for [M + H] C_28_H_21_FNO_3_, *m*/*z*: 438.1500, found: 438.1500

Methyl-2-(((benzyloxy)carbonyl)amino)-2′-hydroxy-[1,1′-binaphthalene]-6-carboxylate (**3g**) Yellowish solid. Yield: 93%. ^1^H-NMR (400 MHz, CDCl_3_) δ 8.62 (d, *J* = 9.2 Hz, 1H), 8.41 (d, *J* = 2.0 Hz, 1H), 8.03 (d, *J* = 9.2 Hz, 1H), 8.01 (d, *J* = 8.8 Hz, 1H), 7.93 (d, *J* = 7.6 Hz, 1H), 7.75 (dd, *J* = 9.2, 2.0 Hz, 1H), 7.44 (d, *J* = 9.2 Hz, 1H), 7.40 (t, *J* = 8.0 Hz, 1H), 7.35–7.26 (m, 6H), 7.16 (d, *J* = 8.8 Hz, 1H), 6.98 (d, *J* = 8.0 Hz, 1H), 6.70 (s, 1H), 6.27 (s, 1H), 5.07 (d, *J* = 12.4 Hz, 1H), 5.03 (d, *J* = 12.4 Hz, 1H), 3.86 (s, 3H). ^13^C-NMR (100 MHz, CDCl_3_) δ 167.1, 153.4, 152.6, 138.0, 135.6, 135.5, 133.2, 131.5, 131.5, 131.2, 129.5, 129.4, 128.6, 128.5, 128.4, 128.4, 127.5, 126.5, 126.1, 125.4, 124.0, 123.9, 119.9, 118.5, 117.1, 112.0, 67.3, 52.3. HRMS (ESI) calcd for [M + H] C_30_H_24_NO_5_, *m*/*z*: 478.1649, found: 478.1649.

Benzyl-(2′-hydroxy-7-methoxy-[1,1′-binaphthalen]-2-yl)carbamate (**3h**) Yellowish solid. Yield: 97%. ^1^H-NMR (400 MHz, CDCl_3_) δ 8.39 (d, *J* = 8.8 Hz, 1H), 7.99 (d, *J* = 9.2 Hz, 1H), 7.98 (d, *J* = 9.2 Hz, 1H), 7.93 (d, *J* = 8.0 Hz, 1H), 7.83 (d, *J* = 8.8 Hz, 1H), 7.42–7.39 (m, 2H), 7.37–7.26 (m, 6H), 7.14–7.10 (m, 2H), 6.54 (s, 1H), 6.45 (d, *J* = 2.8 Hz, 1H), 5.48 (s, 1H), 5.08 (d, *J* = 12.4 Hz, 1H), 5.04 (d, *J* = 12.0 Hz, 1H), 3.51 (s, 3H). ^13^C-NMR (100 MHz, CDCl_3_) δ 158.9, 153.6, 152.1, 136.4, 135.8, 134.4, 133.0, 131.3, 130.0, 129.9, 129.4, 128.6, 128.5, 128.3, 128.3, 127.4, 126.3, 124.2, 124.0, 118.0, 117.5, 117.3, 115.9, 112.8, 103.9, 67.1, 55.1. HRMS (ESI) calcd for [M + H] C_29_H_24_NO_4_, *m*/*z*: 450.1700, found: 450.1699.

Benzyl-(2′-hydroxy-7-phenyl-[1,1′-binaphthalen]-2-yl)carbamate (**3i**) White solid. Yield: 98%. ^1^H-NMR (400 MHz, CDCl_3_) δ 8.60 (d, *J* = 8.8 Hz, 1H), 8.12 (d, *J* = 9.2 Hz, 1H), 8.04 (d, *J* = 8.4 Hz, 1H), 8.03 (d, *J* = 8.8 Hz, 1H), 7.97 (d, *J* = 8.0 Hz, 1H), 7.76 (d, *J* = 8.4 Hz, 1H), 7.48–7.30 (m, 14H), 7.19 (dd, *J* = 8.4, 2.0 Hz, 1H), 6.65 (s, 1H), 5.67 (s, 1H), 5.12 (d, *J* = 12.0 Hz, 1H), 5.08 (d, *J* = 12.4 Hz, 1H). ^13^C-NMR (100 MHz, CDCl_3_) δ 153.7, 152.3, 141.0, 140.2, 136.3, 135.8, 133.4, 133.3, 131.4, 130.1, 130.0, 129.5, 128.9, 128.8, 128.6, 128.6, 128.4, 128.3, 127.5, 127.5, 127.5, 125.2, 124.2, 124.1, 123.1, 119.9, 118.1, 117.6, 112.7, 67.2. HRMS (ESI) calcd for [M + H] C_34_H_26_NO_3_, *m*/*z*: 496.1907, found: 496.1907.

Benzyl-(7-bromo-2′-hydroxy-[1,1′-binaphthalen]-2-yl)carbamate (**3j**) White solid. Yield: 95%. ^1^H-NMR (400 MHz, CDCl_3_) δ 8.54 (d, *J* = 9.2 Hz, 1H), 8.00 (dd, *J* = 8.8, 3.2 Hz, 2H), 7.94 (d, *J* = 8.0 Hz, 1H), 7.78 (d, *J* = 8.4 Hz, 1H), 7.52 (dd, *J* = 8.4, 2.0 Hz, 1H), 7.44–7.25 (m, 9H), 7.02 (d, *J* = 8.4 Hz, 1H), 6.54 (s, 1H), 5.47 (s, 1H), 5.06 (d, *J* = 12.4 Hz, 1H), 5.02 (d, *J* = 12.4 Hz, 1H). ^13^C-NMR (100 MHz, CDCl_3_) δ 153.5, 152.1, 136.7, 135.6, 134.3, 133.0, 131.6, 130.2, 130.0, 129.5, 129.2, 128.7, 128.6, 128.6, 128.4, 128.3, 127.7, 127.1, 124.1, 123.9, 122.0, 120.0, 118.1, 116.4, 111.9, 67.3. HRMS (ESI) calcd for [M + H] C_28_H_21_BrNO_3_, *m*/*z*: 498.0700, found: 498.0699.

Benzyl-(7′-bromo-2′-hydroxy-[1,1′-binaphthalen]-2-yl)carbamate (**3k**) Yellowish solid. Yield: 94%. ^1^H-NMR (400 MHz, CDCl_3_) δ 8.52 (d, *J* = 8.8 Hz, 1H), 8.08 (d, *J* = 9.2 Hz, 1H), 7.95 (d, *J* = 8.0 Hz, 1H), 7.94 (d, *J* = 8.8 Hz, 1H), 7.77 (d, *J* = 8.8 Hz, 1H), 7.49–7.45 (m, 2H), 7.39 (d, *J* = 8.8 Hz, 1H), 7.36–7.28 (m, 6H), 7.19 (d, *J* = 2.4 Hz, 1H), 7.11 (d, *J* = 8.8 Hz, 1H), 6.48 (s, 1H), 5.49 (s, 1H), 5.09 (d, *J* = 12.4 Hz, 1H), 5.05 (d, *J* = 12.0 Hz, 1H). ^13^C-NMR (100 MHz, CDCl_3_) δ 153.6, 153.0, 135.9, 135.6, 134.6, 132.8, 131.2, 130.9, 130.7, 130.1, 128.6, 128.4, 128.4, 128.4, 127.8, 127.6, 127.5, 126.1, 125.4, 124.9, 122.1, 119.9, 118.5, 116.2, 112.2, 67.3. HRMS (ESI) calcd for [M + H] C_28_H_21_BrNO_3_, *m*/*z*: 498.0700, found: 498.0701.

Methyl-2′-(((benzyloxy)carbonyl)amino)-2-hydroxy-[1,1′-binaphthalene]-6-carboxyl- ate (**3l**) Yellowish solid. Yield: 90%. ^1^H-NMR (400 MHz, Acetone-*d*_6_) δ 8.96 (s, 1H), 8.62 (d, *J* = 2.0 Hz, 1H), 8.44 (d, *J* = 9.2 Hz, 1H), 8.15 (d, *J* = 8.0 Hz, 1H), 8.06 (d, *J* = 8.8 Hz, 1H), 7.97 (d, *J* = 8.4 Hz, 1H), 7.78 (dd, *J* = 8.8, 2.0 Hz, 1H), 7.50 (d, *J* = 9.2 Hz, 1H), 7.43–7.39 (m, 2H), 7.29–7.20 (m, 6H), 7.08 (d, *J* = 8.4 Hz, 1H), 7.04 (d, *J* = 8.8 Hz, 1H), 5.04 (s, 2H), 3.89 (s, 3H). ^13^C-NMR (100 MHz, Acetone-*d*_6_) δ 171.7, 161.1, 158.9, 141.9, 141.7, 140.8, 138.4, 137.3, 136.4, 136.1, 134.1, 134.1, 133.5, 133.3, 133.3, 133.1, 133.1, 131.7, 131.2, 130.4, 130.1, 129.9, 129.5, 125.8, 124.9, 119.2, 71.3, 56.6. HRMS (ESI) calcd for [M + H] C_30_H_24_NO_5_, *m*/*z*: 478.1649, found: 478.1651.

Dimethyl-2-(((benzyloxy)carbonyl)amino)-2′-hydroxy-[1,1′-binaphthalene]-6,6′-dicarboxylate (**3m**) Yellowish solid. Yield: 81%. ^1^H-NMR (400 MHz, CDCl_3_) δ 8.58 (d, *J* = 1.6 Hz, 1H), 8.56 (d, *J* = 8.8 Hz, 1H), 8.39 (d, *J* = 2.0 Hz, 1H), 8.05 (d, *J* = 8.8 Hz, 1H), 8.01 (d, *J* = 9.2 Hz, 1H), 7.78 (dd, *J* = 8.8, 1.6 Hz, 1H), 7.72 (dd, *J* = 8.8, 1.6 Hz, 1H), 7.43 (d, *J* = 9.2 Hz, 1H), 7.31–7.26 (m, 3H), 7.24–7.21 (m, 2H), 7.05 (d, *J* = 8.8 Hz, 1H), 6.94 (d, *J* = 8.8 Hz, 1H), 6.60 (s, 1H), 6.34 (s, 1H), 5.02 (s, 2H), 3.91 (s, 3H), 3.84 (s, 3H). ^13^C-NMR (100 MHz, CDCl_3_) δ 167.1, 167.0, 154.6, 153.3, 138.1, 135.7, 135.4, 135.3, 133.0, 131.8, 131.6, 131.2, 129.6, 128.6, 128.5, 128.4, 128.4, 127.0, 126.6, 126.3, 125.6, 125.1, 124.0, 120.0, 119.3, 116.3, 112.3, 67.4, 52.3, 52.2. HRMS (ESI) calcd for [M + H] C_32_H_26_NO_7_, *m*/*z*: 536.1704, found: 536.1706.

Benzyl-(6,6′-dibromo-2′-hydroxy-[1,1′-binaphthalen]-2-yl)carbamate (**3n**) White solid. Yield: 90%. ^1^H-NMR (400 MHz, Acetone-*d*_6_) δ 8.74 (s, 1H), 8.48 (d, *J* = 9.2 Hz, 1H), 8.19 (d, *J* = 2.4 Hz, 1H), 8.12 (d, *J* = 2.0 Hz, 1H), 8.04 (d, *J* = 9.2 Hz, 1H), 7.97 (d, *J* = 8.8 Hz, 1H), 7.44 (d, *J* = 8.8 Hz, 1H), 7.43 (s, 1H), 7.38 (dd, *J* = 9.2, 2.4 Hz, 1H), 7.34 (dd, *J* = 8.8, 2.0 Hz, 1H), 7.32–7.26 (m, 3H), 7.23–7.21 (m, 2H), 6.99 (d, *J* = 8.8 Hz, 1H), 6.86 (d, *J* = 8.8 Hz, 1H), 5.06 (d, *J* = 12.4 Hz, 1H), 5.03 (d, *J* = 12.4 Hz, 1H). ^13^C-NMR (100 MHz, Acetone-*d*_6_) δ 154.1, 153.6, 136.6, 136.2, 132.5, 132.0, 131.8, 130.3, 130.2, 130.0, 130.0, 129.9, 129.6, 128.3, 128.1, 127.9, 127.9, 127.5, 126.1, 121.7, 120.5, 120.0, 118.0, 116.5, 113.4, 66.2. HRMS (ESI) calcd for [M + H] C_28_H_20_Br_2_NO_3_, *m*/*z*: 575.9805, found: 575.9808.

Benzyl-(7,7′-dibromo-2′-hydroxy-[1,1′-binaphthalen]-2-yl)carbamate (**3o**) Yellowish solid. Yield: 85%. ^1^H-NMR (400 MHz, CDCl_3_) δ 8.53 (dd, *J* = 9.2, 2.0 Hz, 1H), 8.02 (d, *J* = 9.2 Hz, 1H), 7.94 (d, *J* = 8.8 Hz, 1H), 7.79 (d, *J* = 8.8 Hz, 1H), 7.78 (d, *J* = 8.4 Hz, 1H), 7.53 (dd, *J* = 8.8, 2.0 Hz, 1H), 7.48 (dd, *J* = 8.8, 2.0 Hz, 1H), 7.38–7.32 (m, 4H), 7.29–7.26 (m, 2H), 7.22 (d, *J* = 2.0 Hz, 1H), 7.13 (d, *J* = 1.6 Hz, 1H), 6.44 (s, 1H), 5.49 (s, 1H), 5.08 (d, *J* = 12.4 Hz, 1H), 5.04 (d, *J* = 12.0 Hz, 1H). ^13^C-NMR (100 MHz, CDCl_3_) δ 153.4, 153.0, 136.8, 135.5, 134.4, 134.1, 131.6, 130.5, 130.3, 130.1, 129.2, 128.9, 128.6, 128.5, 128.4, 127.9, 127.7, 126.8, 125.8, 122.4, 122.2, 120.1, 118.6, 115.4, 111.3, 67.4. HRMS (ESI) calcd for [M + H] C_28_H_20_Br_2_NO_3_, *m*/*z*: 575.9805, found: 575.9804.

Benzyl-(2′-hydroxy-7,7′-diphenyl-[1,1′-binaphthalen]-2-yl)carbamate (**3p**) White solid. Yield: 83%. ^1^H-NMR (400 MHz, CDCl_3_) δ 8.58 (d, *J* = 8.8 Hz, 1H), 8.12 (d, *J* = 8.8 Hz, 1H), 8.05–8.01 (m, 3H), 7.74 (dd, *J* = 8.4, 2.0 Hz, 1H), 7.69 (dd, *J* = 8.4, 2.0 Hz, 1H), 7.47–7.41 (m, 7H), 7.39–7.27 (m, 11H), 6.63 (s, 1H), 5.38 (s, 1H), 5.12 (d, *J* = 12.4 Hz, 1H), 5.09 (d, *J* = 12.0 Hz, 1H). ^13^C-NMR (100 MHz, CDCl_3_) δ153.7, 152.5, 141.0, 141.0, 140.4, 140.3, 136.3, 135.7, 133.5, 133.2, 131.2, 130.2, 130.1, 129.1, 129.0, 128.8, 128.8, 128.7, 128.6, 128.4, 128.3, 127.5, 127.5, 127.4, 127.4, 125.2, 123.9, 122.9, 121.9, 120.0, 118.0, 117.2, 112.9, 67.2. HRMS (ESI) calcd for [M + H] C_40_H_30_NO_3_, *m*/*z*: 572.2220, found: 572.2223.

Methyl-2′-((benzyl carbonyl)amino)-2-hydroxy-6′-methoxy-[1,1′-binaphthalene]-6-carboxylate (**3q**) Yellowish solid. Yield: 88%. ^1^H-NMR (400 MHz, CDCl_3_) δ 8.62 (d, *J* = 2.0 Hz, 1H), 8.37 (d, *J* = 8.8 Hz, 1H), 8.04 (d, *J* = 8.8 Hz, 1H), 7.93 (d, *J* = 9.2 Hz, 1H), 7.81 (dd, *J* = 9.2, 2.0 Hz, 1H), 7.42 (d, *J* = 8.8 Hz, 1H), 7.34–7.20 (m, 6H), 7.05 (d, *J* = 8.8 Hz, 1H), 7.00 (d, *J* = 9.2 Hz, 1H), 6.95 (dd, *J* = 9.2, 2.8 Hz, 1H), 6.45 (s, 1H), 5.99 (s, 1H), 5.05 (d, *J* = 12.0 Hz, 1H), 5.00 (d, *J* = 12.0 Hz, 1H), 3.94 (s, 3H), 3.88 (s, 3H). ^13^C-NMR (100 MHz, CDCl_3_) δ 167.2, 157.3, 154.3, 153.9, 135.8, 135.7, 133.7, 132.5, 132.2, 131.5, 129.1, 128.5, 128.3, 128.3, 128.2, 128.1, 126.8, 126.6, 125.4, 124.3, 121.0, 120.0, 119.1, 117.7, 113.4, 106.5, 67.1, 55.3, 52.2. HRMS (ESI) calcd for [M + H] C_31_H_26_NO_6_, *m*/*z*: 508.1755, found: 508.1759.

Benzyl-(6′-fluoro-2′-hydroxy-6-methoxy-[1,1′-binaphthalen]-2-yl)carbamate (**3r**) White solid. Yield: 85%. ^1^H-NMR (400 MHz, CDCl_3_) δ 8.40 (d, *J* = 8.8 Hz, 1H), 7.94 (d, *J* = 8.8 Hz, 1H), 7.89 (d, *J* = 8.8 Hz, 1H), 7.54 (dd, *J* = 9.5, 2.4 Hz, 1H), 7.40 (d, *J* = 8.8 Hz, 1H), 7.37-7.31 (m, 3H), 7.28–7.23 (m, 3H), 7.10–6.98 (m, 4H), 6.46 (s, 1H), 5.60 (s, 1H), 5.07 (d, *J* = 12.4 Hz, 1H), 5.01 (d, *J* = 12.4 Hz, 1H), 3.91 (s, 3H). ^13^C-NMR (100 MHz, CDCl_3_) δ 159.5 (d, *J* = 243.0 Hz), 157.3, 153.9, 151.5 (d, *J* = 2.0 Hz), 135.8, 133.6, 132.2, 130.2 (d, *J* = 5.0 Hz), 130.2, 129.9 (d, *J* = 9.0 Hz), 129.1, 128.6, 128.4, 128.3, 128.1, 120.9, 126.7, 126.5 (d, *J* = 8.0 Hz), 120.0, 119.3, 118.0, 117.4 (d, *J* = 25.0 Hz), 113.5, 111.6 (d, *J* = 21.0 Hz), 106.5, 67.2, 55.4. ^19^F-NMR (376 MHz, CDCl_3_) δ −118.10. HRMS (ESI) calcd for [M + H] C_29_H_23_FNO_4_, *m*/*z*: 468.1606, found: 468.1607.

Benzyl-(6′-fluoro-2′-hydroxy-7-phenyl-[1,1′-binaphthalen]-2-yl)carbamate (**3s**) Yellowish solid. Yield: 84%. ^1^H-NMR (400 MHz, CDCl_3_) δ 8.55 (d, *J* = 9.2 Hz, 1H), 8.10 (d, *J* = 9.2 Hz, 1H), 8.02 (d, *J* = 8.8 Hz, 1H), 7.93 (d, *J* = 8.8 Hz, 1H), 7.75 (dd, *J* = 8.4, 2.0 Hz, 1H), 7.57 (dd, *J* = 9.6, 2.4 Hz, 1H), 7.47–7.44 (m, 3H), 7.41–7.28 (m, 9H), 7.16–7.08 (m, 2H), 6.57 (s, 1H), 5.55 (s, 1H), 5.12 (d, *J* = 12.0 Hz, 1H), 5.06 (d, *J* = 12.4 Hz, 1H). ^13^C-NMR (100 MHz, CDCl_3_) δ 159.6 (d, *J* = 242.0 Hz), 153.7, 151.7 (d, *J* = 2.0 Hz), 140.9, 140.3, 136.2, 135.7, 133.2, 130.5 (d, *J* = 5.0 Hz), 130.2, 130.1, 130.1, 130.0 (d, *J* = 10.0 Hz), 129.0, 128.8, 128.6, 128.5, 128.4, 127.5, 127.5, 126.5 (d, *J* = 8.0 Hz), 125.3, 122.9, 120.0, 119.4, 117.5 (d, *J* = 25.0 Hz), 117.3, 113.0, 111.8 (d, *J* = 21.0 Hz), 67.3. ^19^F-NMR (376 MHz, CDCl_3_) δ −117.88. HRMS (ESI) calcd for [M + H] C_34_H_25_FNO_3_, *m*/*z*: 514.1813, found: 514.1814.

Methyl-2-(((benzyloxy)carbonyl)amino)-7′-bromo-2′-hydroxy-[1,1′-binaphthalene]- 6-carboxylate (**3t**) Yellowish solid. Yield: 86%. ^1^H-NMR (400 MHz, CDCl_3_) δ 8.61 (d, *J* = 9.2 Hz, 1H), 8.41 (d, *J* = 2.0 Hz, 1H), 8.04 (d, *J* = 9.2 Hz, 1H), 7.95 (d, *J* = 8.8 Hz, 1H), 7.77 (d, *J* = 8.8 Hz, 1H), 7.76 (dd, *J* = 8.8, 2.0 Hz, 1H), 7.46 (dd, *J* = 8.4, 2.0 Hz, 1H), 7.42 (d, *J* = 8.8 Hz, 1H), 7.37–7.27 (m, 5H), 7.09 (d, *J* = 8.8 Hz, 1H), 7.09 (d, *J* = 2.0 Hz, 1H), 6.60 (s, 1H), 6.29 (s, 1H), 5.09 (d, *J* = 12.0 Hz, 1H), 5.05 (d, *J* = 12.0 Hz, 1H) 3.87 (s, 3H). ^13^C-NMR (100 MHz, CDCl_3_) δ 167.1, 153.4, 153.3, 138.0, 135.4, 135.3, 134.5, 131.9, 131.5, 131.3, 130.2, 129.6, 128.6, 128.5, 128.4, 127.8, 127.5, 126.7, 126.3, 125.8, 125.1, 122.2, 119.9, 118.9, 116.1, 111.4, 67.4, 52.3. HRMS (ESI) calcd for [M + H] C_30_H_23_BrNO_5_, *m*/*z*: 556.0754, found: 556.0756.

Benzyl-(7-bromo-2′-hydroxy-7′-phenyl-[1,1′-binaphthalen]-2-yl)carbamate (**3u**) Yellowish solid. Yield: 88%. ^1^H-NMR (400 MHz, CDCl_3_) δ 8.50 (d, *J* = 9.2 Hz, 1H), 7.96 (d, *J* = 9.2 Hz, 2H), 7.95 (d, *J* = 8.4 Hz, 1H), 7.73 (d, *J* = 8.4 Hz, 1H), 7.62 (dd, *J* = 8.4, 1.6 Hz, 1H), 7.47 (dd, *J* = 8.8, 2.0 Hz, 1H), 7.38–7.18 (m, 12H), 7.13 (d, *J* = 2.0 Hz, 1H), 6.52 (s, 1H), 5.35 (s, 1H), 5.02 (d, *J* = 12.0 Hz, 1H), 4.99 (d, *J* = 12.4 Hz, 1H). ^13^C-NMR (100 MHz, CDCl_3_) δ 153.6, 152.5, 140.9, 140.5, 136.8, 135.6, 134.3, 133.3, 131.4, 130.3, 130.0, 129.3, 129.2, 128.8, 128.8, 128.7, 128.6, 128.4, 128.3, 127.5, 127.5, 127.1, 124.0, 122.1, 121.6, 120.1, 118.1, 116.2, 112.2, 67.3. HRMS (ESI) calcd for [M + H] C_34_H_25_BrNO_3_, *m*/*z*: 574.1013, found: 574.1016.

Benzyl-(1-(10-hydroxyphenanthren-9-yl)naphthalen-2-yl)carbamate (**3v**) Yellowish solid. Yield: 91%. ^1^H-NMR (400 MHz, CDCl_3_) δ 8.85 (d, *J* = 8.0 Hz, 1H), 8.79 (d, *J* = 8.0 Hz, 1H), 8.63 (d, *J* = 8.8 Hz, 1H), 8.51 (dd, *J* = 8.0, 1.6 Hz, 1H), 8.13 (d, *J* = 9.2 Hz, 1H), 7.98 (d, *J* = 8.0 Hz, 1H), 7.87–7.83 (m, 1H), 7.79–7.75 (m, 1H), 7.59–7.55 (m, 1H), 7.49–7.45 (m, 1H), 7.41–7.37 (m, 1H), 7.33–7.28 (m, 5H), 7.27–7.22 (m, 2H), 7.10 (dd, *J* = 8.4, 1.6 Hz, 1H), 6.68 (s, 1H), 5.61 (s, 1H), 5.06 (d, *J* = 12.4 Hz, 1H), 5.02 (d, *J* = 12.0 Hz, 1H). ^13^C-NMR (100 MHz, CDCl_3_) δ 153.6, 148.4, 136.3, 135.7, 133.0, 132.0, 131.6, 130.9, 130.5, 128.5, 128.3, 128.3, 128.3, 128.0, 127.7, 127.5, 127.0, 126.9, 125.3, 125.2, 125.1, 124.8, 124.8, 123.6, 123.0, 122.8, 119.7, 116.7, 108.9, 67.1. HRMS (ESI) calcd for [M + H] C_32_H_24_NO_3_, *m*/*z*: 470.1751, found: 470.1753.

Benzyl-(1-(10-hydroxyphenanthren-9-yl)-7-methoxynaphthalen-2-yl)carbamate (**3w**) White solid. Yield: 90%. ^1^H-NMR (400 MHz, CDCl_3_) δ 8.79 (dd, *J* = 8.4, 1.2 Hz, 1H), 8.72 (dd, *J* = 8.4, 1.6 Hz, 1H), 8.44 (dd, *J* = 8.4, 1.6 Hz, 1H), 8.41 (d, *J* = 9.2 Hz, 1H), 7.99 (d, *J* = 9.2 Hz, 1H), 7.83 (d, *J* = 9.2 Hz, 1H), 7.82–7.78 (m, 1H), 7.73–7.69 (m, 1H), 7.54–7.50 (m, 1H), 7.36–7.32 (m, 1H), 7.27–7.23 (m, 3H), 7.20–7.17 (m, 2H), 7.10–7.06 (m, 2H), 6.54 (s, 1H), 6.48 (d, *J* = 2.4 Hz, 1H), 5.47 (s, 1H), 5.03 (d, *J* = 12.4 Hz, 1H), 5.00 (d, *J* = 12.4 Hz, 1H), 3.43 (s, 3H). ^13^C-NMR (100 MHz, CDCl_3_) δ 159.0, 153.6, 148.2, 136.8, 135.7, 134.4, 131.9, 131.3, 130.3, 129.9, 128.5, 128.3, 128.3, 128.0, 127.7, 127.0, 126.8, 126.3, 125.0, 124.8, 124.7, 123.6, 123.0, 122.7, 117.5, 117.2, 115.3, 109.0, 103.8, 67.1, 55.1. HRMS (ESI) calcd for [M + H] C_33_H_26_NO_4_, *m*/*z*: 500.1857, found: 500.1859.

Methyl-6-(((benzyloxy)carbonyl)amino)-5-(10-hydroxyphenanthren-9-yl)-2-naphth- oate (**3x**) Yellowish solid. Yield: 92%. ^1^H-NMR (400 MHz, CDCl_3_) δ 8.79 (d, *J* = 7.2 Hz, 1H), 8.73 (dd, *J* = 8.4, 1.2 Hz, 1H), 8.68 (d, *J* = 8.8 Hz, 1H), 8.57 (d, *J* = 2.0 Hz, 1H), 8.46 (dd, *J* = 8.0, 1.6 Hz, 1H), 8.13 (d, *J* = 9.6 Hz, 1H), 7.84–7.79 (m, 1H), 7.77 (dd, *J* = 8.8, 1.6 Hz, 1H), 7.74–7.70 (m, 1H), 7.55–7.50 (m, 1H), 7.35–7.30 (m, 1H), 7.28–7.24 (m, 3H), 7.23–7.19 (m, 3H), 6.96 (dd, *J* = 8.4, 1.6 Hz, 1H), 6.70 (s, 1H), 5.65 (s, 1H), 5.04 (d, *J* = 12.4 Hz, 1H), 5.01 (d, *J* = 12.4 Hz, 1H), 3.90 (s, 3H). ^13^C-NMR (100 MHz, CDCl_3_) δ 167.0, 153.3, 148.5, 138.5, 135.4, 135.4, 132.0, 131.9, 131.3, 131.3, 129.7, 128.5, 128.4, 128.4, 128.2, 127.8, 127.1, 126.9, 126.8, 126.6, 125.4, 125.0, 124.9, 124.6, 123.6, 123.1, 122.7, 119.9, 116.3, 108.1, 67.3, 52.3. HRMS (ESI) calcd for [M + H] C_34_H_26_NO_5_, *m*/*z*: 528.1806, found: 528.1807.

Benzyl-(1-(2-hydroxyphenyl)naphthalen-2-yl)carbamate (**3y**) Yellowish solid. Yield: 75%. ^1^H-NMR (400 MHz, CDCl_3_) δ 8.36 (d, *J* = 8.4 Hz, 1H), 7.90–7.86 (m, 2H), 7.59–7.53 (m, 1H), 7.42–7.38 (m, 2H), 7.33–7.24 (m, 9H), 6.42 (s, 1H), 5.36 (s, 1H), 5.08 (s, 2H). ^13^C-NMR (100 MHz, CDCl_3_) δ 153.5, 151.3, 137.4, 135.8, 133.0, 132.0, 130.8, 130.1, 129.2, 128.5, 128.3, 128.3, 128.3, 127.3, 124.4, 124.1, 123.8, 122.7, 120.5, 117.8, 115.3, 67.0. HRMS (ESI) calcd for [M + H] C_24_H_20_NO_3_, *m*/*z*: 370.1438, found: 370.1435.

Benzyl-(1-(5-bromo-2-hydroxyphenyl)naphthalen-2-yl)carbamate (**3z**) White solid. Yield: 72%. ^1^H-NMR (400 MHz, CDCl_3_) δ 8.20 (d, *J* = 8.8 Hz, 1H), 7.87 (d, *J* = 9.2 Hz, 1H), 7.80 (d, *J* = 7.6 Hz, 1H), 7.45–7.29 (m, 8H), 7.28–7.22 (m, 2H), 6.92 (d, *J* = 8.4 Hz, 1H), 6.58 (s, 1H), 5.47 (s, 1H), 5.05 (d, *J* = 12.4 Hz, 1H), 5.00 (d, *J* = 12.0 Hz, 1H). ^13^C-NMR (100 MHz, CDCl_3_) δ 153.8, 153.2, 135.7, 134.4, 134.0, 133.5, 132.5, 130.8, 130.2, 128.7, 128.5, 128.4, 128.2, 127.4, 125.4, 124.9, 123.2, 120.3, 119.7, 118.5, 113.2, 67.4. HRMS (ESI) calcd for [M + H] C_24_H_19_BrNO_3_, *m*/*z*: 448.0543, found: 448.0540.

Benzyl-(1-(3-fluoro-6-hydroxy-2,4-dimethylphenyl)naphthalen-2-yl)carbamate (**3aa**) Yellowish solid. Yield: 72%. ^1^H-NMR (400 MHz, CDCl_3_) δ 7.96 (d, *J* = 7.6 Hz, 1H), 7.91-7.87 (m, 2H), 7.42–7.14 (m, 9H), 6.16 (s, 1H), 5.45 (s, 1H), 5.05 (d, *J* = 12.0 Hz, 1H), 5.01 (d, *J* = 12.0 Hz, 1H), 2.41 (d, *J* = 2.4 Hz, 3H), 1.86 (d, *J* = 2.8 Hz, 3H). ^13^C-NMR (100 MHz, CDCl_3_) δ 156.8 (d, *J* = 240.3 Hz), 153.8, 151.4, 135.8, 132.6, 131.0, 129.3, 128.5, 128.5, 128.3, 128.2, 127.5, 126.1 (d, *J* = 19.0 Hz), 125.8 (d, *J* = 17.9 Hz), 123.9, 123.5, 122.1, 121.0, 117.9, 113.5, 113.4, 67.0, 15.1 (d, *J* = 3.7 Hz), 11.9 (d, *J* = 4.4 Hz). ^19^F-NMR (375 MHz, CDCl_3_) δ −123.4. HRMS (ESI) calcd for [M + H] C_26_H_23_FNO_3_, *m*/*z*: 416.1657, found: 416.1658.

Benzyl-(2-(2-hydroxynaphthalen-1-yl)-3,5-dimethylphenyl)carbamate (**3ab**) White solid. Yield: 62%. ^1^H-NMR (400 MHz, CDCl_3_) δ 8.03 (s, 1H), 7.90 (d, *J* = 8.8 Hz, 1H), 7.88–7.85 (m, 1H), 7.41–7.30 (m, 6H), 7.27–7.15 (m, 3H), 7.02 (d, *J* = 1.6 Hz, 1H), 7.02 (s, 1H), 6.25 (s, 1H), 5.04 (s, 2H), 2.47 (s, 3H), 1.90 (s, 3H). ^13^C-NMR (100 MHz, CDCl_3_) δ 153.5, 151.4, 139.9, 139.3, 137.3, 135.9, 132.7, 130.8, 129.4, 128.5, 128.5, 128.3, 128.2, 127.3, 126.7, 123.8, 123.7, 118.8, 118.3, 117.7, 114.1, 66.9, 21.7, 19.8. HRMS (ESI) calcd for [M + H] C_26_H_24_NO_3_, *m*/*z*: 398.1751, found: 398.1750.

Benzyl-(2-(2-hydroxynaphthalen-1-yl)phenyl)carbamate (**3ac**) Yellowish solid. Yield: 68%. ^1^H-NMR (400 MHz, CDCl_3_) δ 8.28 (d, *J* = 9.2 Hz, 1H), 7.84 (d, *J* = 9.2 Hz, 1H), 7.76 (d, *J* = 8.0 Hz, 1H), 7.35–7.21 (m, 9H), 7.08–6.99 (m, 3H), 6.99 (s, 1H), 5.05 (s, 1H), 5.09 (d, *J* = 12.0 Hz, 1H), 5.04 (d, *J* = 12.0 Hz, 1H). ^13^C-NMR (100 MHz, CDCl_3_) δ 153.9, 153.7, 135.9, 134.6, 132.7, 131.7, 130.7, 130.6, 129.8, 128.6, 128.4, 128.4, 128.2, 127.1, 125.1, 125.1, 121.5, 120.7, 120.0, 119.8, 116.6, 67.2. HRMS (ESI) calcd for [M + H] C_24_H_20_NO_3_, *m*/*z*: 370.1438, found: 370.1438.

Benzyl-(1-(2-hydroxy-2′-iodo-6,6′-dimethyl-[1,1′-biphenyl]-3-yl)naphthalen-2-yl)- carbamate (**3ad**) Yellowish solid. 72% yield, dr = 1.2/1. First diastereomer: ^1^H-NMR (400 MHz, CDCl_3_) δ 8.48 (d, *J* = 9.2 Hz, 1H), 7.96 (d, *J* = 9.2 Hz, 1H), 7.90–7.88 (m, 1H), 7.80 (d, *J* = 8.0 Hz, 1H), 7.50–7.30 (m, 9H), 7.19–7.10 (m, 2H), 7.02 (t, *J* = 8.0 Hz, 1H), 6.94 (s, 1H), 5.16–5.09 (m, 2H), 4.62 (s, 1H), 2.20 (s, 3H), 2.06 (s, 3H). ^13^C-NMR (100 MHz, CDCl_3_) δ 153.5, 149.9, 140.2, 138.8, 138.2, 136.9, 135.8, 134.6, 133.1, 131.6, 131.6, 130.5, 130.1, 129.8, 129.4, 128.6, 128.5, 128.3, 128.1, 126.7, 125.1, 124.7, 123.2, 120.2, 119.3, 118.6, 102.2, 67.1, 21.4, 19.6. HRMS (ESI) calcd for [M + H] C_32_H_27_INO_3_, *m*/*z*: 600.1030, found: 600.1031. Second diastereomer: ^1^H-NMR (400 MHz, CDCl_3_) δ 8.37 (d, *J* = 8.4 Hz, 1H), 7.95 (d, *J* = 9.2 Hz, 1H), 7.89–7.87 (m, 1H), 7.84 (d, *J* = 8.0 Hz, 1H), 7.66–7.62 (m, 1H), 7.48–7.31 (m, 8H), 7.19 (d, *J* = 7.6 Hz, 1H), 7.11 (d, *J* = 7.6 Hz, 1H), 7.02 (t, *J* = 8.0 Hz, 1H), 6.79 (s, 1H), 5.21–5.14 (m, 2H), 4.65 (s, 1H), 2.08 (s, 6H). ^13^C-NMR (100 MHz, CDCl_3_) δ 153.5, 149.9, 140.3, 138.8, 138.3, 137.0, 136.0, 134.1, 132.8, 131.6, 131.4, 130.7, 130.2, 129.8, 129.5, 128.6, 128.3, 128.2, 126.9, 125.7, 124.9, 123.2, 121.0, 119.6, 118.7, 115.6, 101.9, 67.1, 21.3, 19.6. HRMS (ESI) calcd for [M + H] C_32_H_27_INO_3_, *m*/*z*: 600.1030, found: 600.1030.

*N*^2^-methyl-[1,1′-binaphthalene]-2,2′-diamine (**5a**) Yellowish solid. Yield: 52%. ^1^H-NMR (400 MHz, CDCl_3_) δ 7.92 (d, *J* = 8.8 Hz, 1H), 7.83–7.79 (m, 3H), 7.27–7.24 (m, 2H), 7.22–7.17 (m, 3H), 7.15 (d, *J* = 8.4 Hz, 1H), 7.04 (d, *J* = 8.4 Hz, 1H), 7.02 (dd, *J* = 9.2, 2.8 Hz, 1H), 3.85 (brs, 1H), 2.85 (s, 3H), 2.55 (brs, 2H). ^13^C-NMR (100 MHz, CDCl_3_) δ 144.9, 142.5, 133.5, 133.0, 129.3, 129.1, 128.1, 127.8, 127.7, 127.2, 126.4, 126.3, 123.6, 123.2, 122.0, 121.4, 117.9, 113.0, 112.1, 111.5, 30.7. HRMS (ESI) calcd for [M + H] C_21_H_19_N_2_, *m*/*z*: 299.1543, found: 299.1544.

*N*^2^-phenyl-[1,1′-binaphthalene]-2,2′-diamine (**5b**) White solid. Yield: 50%. ^1^H-NMR (400 MHz, CDCl_3_) δ 7.93–7.84 (m, 4H), 7.76 (d, *J* = 9.2 Hz, 1H), 7.48–7.06 (m, 11H), 6.98 (t, *J* = 7.6 Hz, 1H), 5.67 (brs, 1H), 3.22 (brs, 2H). ^13^C-NMR (100 MHz, CDCl_3_) δ 142.9, 142.8, 140.2, 134.0, 133.8, 129.8, 129.5, 129.3, 129.2, 128.5, 128.3, 128.2, 127.1, 126.9, 124.6, 123.9, 123.4, 122.6, 122.0, 119.8, 118.4, 118.0, 116.9, 112.1. HRMS (ESI) calcd for [M + H] C_26_H_21_N_2_, *m*/*z*: 361.1699, found: 361.1699.

*N*^2^-benzyl-[1,1′-binaphthalene]-2,2′-diamine (**5c**) Yellow solid. Yield: 50%. ^1^H-NMR (400 MHz, CDCl_3_) δ 7.86–7.78 (m, 4H), 7.31–7.18 (m, 11H), 7.15–7.13 (m, 1H), 7.08–7.05 (m, 1H), 4.44 (s, 2H), 3.92 (brs, 3H). ^13^C-NMR (100 MHz, CDCl_3_) δ 143.9, 143.0, 139.8, 134.0, 133.6, 129.7, 129.6, 128.6, 128.5, 128.2, 128.2, 127.8, 126.9, 126.9, 126.9, 126.8, 124.2, 123.8, 122.5, 122.0, 118.4, 114.4, 112.5, 112.3, 47.7. HRMS (ESI) calcd for [M + H] C_27_H_23_N_2_, *m*/*z*: 375.1856, found: 375.1855.

*N*^2^-benzyl-6′-fluoro-[1,1′-binaphthalene]-2,2′-diamine (**5d**) Light brown solid. Yield: 56%. ^1^H-NMR (400 MHz, CDCl_3_) δ 7.84 (d, *J* = 9.2 Hz, 1H), 7.82–7.79 (m, 1H), 7.78 (d, *J* = 8.8 Hz, 1H), 7.46 (dd, *J* = 10.0, 2.8 Hz, 1H), 7.28–7.20 (m, 9H), 7.12–7.09 (m, 1H), 7.05–7.00 (m, 2H), 4.44 (s, 2H), 4.19 (brs, 1H), 3.72 (brs, 2H). ^13^C-NMR (100 MHz, CDCl_3_) δ 158.9 (d, *J* = 241.0 Hz), 143.9, 142.4 (d, *J* = 2.0 Hz), 139.8, 133.5, 130.9, 129.7, 128.9 (d, *J* = 8.0 Hz), 128.8 (d, *J* = 5.0 Hz), 128.5, 128.2, 127.7, 127.0, 126.9, 126.8, 126.5 (d, *J* = 8.0 Hz), 123.6, 122.1, 119.6, 116.7 (d, *J* = 24.0 Hz), 114.3, 112.7, 112.1, 111.3 (d, *J* = 20.0 Hz), 47.7. ^19^F-NMR (376 MHz, CDCl_3_) δ −120.50. HRMS (ESI) calcd for [M + H] C_27_H_22_FN_2_, *m*/*z*: 393.1762, found: 393.1762

## 4. Conclusions

In summary, a copper-catalyzed domino reaction toward NOBIN and BINAM derivatives has been established employing diaryliodonium salts as arylation reagents. The results from the control experiments substantiated that the copper catalyst played a key role in improving the yield during the arylation process. This reaction consisting of facile *O-*/*N*-arylation and [3,3]-sigmatropic rearrangement sequence proceeds under mild conditions and displayed good substrate generality and excellent efficiency. In addition, a group of biaryl amino alcohols (including a diaxial structure) and BINAM derivatives were synthesized in moderate or good yields under identical conditions.

## Figures and Tables

**Table 1:**
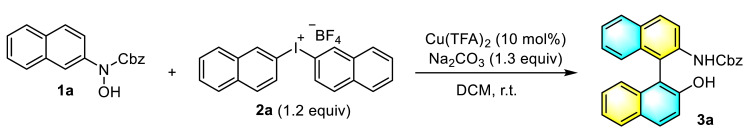
Optimization of the reaction conditions involving *N*-naphthylhydroxylamine *^a^*.

Entry	Variation from the Optimized Conditions	Yield (%) *^b^*
1	none	98
2	toluene instead of DCM	80
3	THF instead of DCM	90
4	MeCN instead of DCM	88
5	EA instead of DCM	86
6	Cu(OAc)_2_ instead of Cu(TFA)_2_	89
7	Cu(OTf)_2_ instead of Cu(TFA)_2_	82
8	CuOTf instead of Cu(TFA)_2_	89
9	CuI instead of Cu(TFA)_2_	86
10	K_2_CO_3_ instead of Na_2_CO_3_	93
11	Cs_2_CO_3_ instead of Na_2_CO_3_	90
12	NaOH instead of Na_2_CO_3_	75
13	NaO*^t^*Bu instead of Na_2_CO_3_	66
14	Et_3_N instead of Na_2_CO_3_	73

*^a^* All reactions were performed with Cu(TFA)_2_ (10 mol%), **1a** (0.10 mmol), **2a** (0.12 mmol), and base (0.13 mmol) in DCM (2.0 mL) at room temperature; *^b^* Yield was determined by ^1^H-NMR analysis of the crude reaction mixture using 1,3,5-trimethoxybenzene as the internal standard.

## Data Availability

Data is contained within the article and Supplementary Materials.

## Supplementary Materials

The following are available online: Table S1. Solvent and catalysts screenings for the reaction with *N*-naphthylhydroxylamine; Table S2. Base and loading screenings for the reaction with *N*-naphthylhydroxylamine; III. Supplementary Experimental Procedures; V. Copies of NMR spectra.

## Sample Availability

Samples of the compounds are not available from the authors.

## References

[1] Kočovský P., Vyskočil Š., Smrčina M. (2003). Non-Symmetrically Substituted 1,1‘-Binaphthyls in Enantioselective Catalysis. Chem. Rev..

[2] Ding K., Li X., Ji B., Guo H., Kitamura M. (2005). Ten Years of Research on NOBIN Chemistry. Curr. Org. Synth..

[3] Ding K., Guo H., Li X., Yuan Y., Wang Y. (2005). Synthesis of NOBIN Derivatives for Asymmetric Catalysis. Top. Catal..

[4] Zhou Q.-L. (2011). Privileged Chiral Ligands and Catalysts.

[5] Carreira E.M., Lee W., Singer R.A. (1995). Catalytic, Enantioselective Acetone Aldol Additions with 2-Methoxypropene. J. Am. Chem. Soc..

[6] Van Veldhuizen J.J., Garber S.B., Kingsbury J.S., Hoveyda A.H. (2002). A Recyclable Chiral Ru Catalyst for Enantioselective Olefin Metathesis. Efficient Catalytic Asymmetric Ring-Opening/Cross Metathesis in Air. J. Am. Chem. Soc..

[7] Yan Y., Zhang X. (2006). A Hybrid Phosphorus Ligand for Highly Enantioselective Asymmetric Hydroformylation. J. Am. Chem. Soc..

[8] Uraguchi D., Kinoshita N., Ooi T. (2010). Catalytic Asymmetric Protonation of α-Amino Acid-Derived Ketene Disilyl Acetals UsingP-Spiro Diaminodioxaphosphonium Barfates as Chiral Proton. J. Am. Chem. Soc..

[9] Tan B., Candeias N.R., Barbas C.F. (2011). Construction of Bispirooxindoles Containing Three Quaternary Stereocentres in a Cascade Using a Single Multifunctional Organocatalyst. Nat. Chem..

[10] Vallavoju N., Selvakumar S., Jockusch S., Sibi M.P., Sivaguru J. (2014). Enantioselective Organo-Photocatalysis Mediated by Atropisomeric Thiourea Derivatives. Angew. Chem. Int. Ed..

[11] Lipshutz B.H., Buzard D., Olsson C., Noson K. (2004). A modular route to nonracemic cyclo-NOBINs. Preparation of the parent ligand for homo- and heterogeneous catalysis. Tetrahedron.

[12] Li Q., Green L., Venkataraman N., Shiyanovskaya I., Khan A., Urbas A., Doane J.W. (2007). Reversible Photoswitchable Axially Chiral Dopants with High Helical Twisting Power. J. Am. Chem. Soc..

[13] Ritter N., Senkovska I., Kaskel S., Weber J. (2011). Towards Chiral Microporous Soluble Polymers-Binaphthalene-Based Polyi-mides. Macromol. Rapid Commun..

[14] Singer R.A., Buchwald S.L. (1999). Preparation of 2-Amino-2′-hydroxy-1,1’-binaphthyl and *N*-arylated 2-Amino-1,1’-binaphthyl Derivatives via Palladium-Catalyzed Amination. Tetrahedron Lett..

[15] Patel D., Breitbach Z.S., Woods R.M., Lim Y., Wang A., Foss F.W., Armstrong D.W. (2016). Gram Scale Conversion ofR-BINAM toR-NOBIN. J. Org. Chem..

[16] Chang X., Zhang Q., Guo C. (2019). Switchable Smiles Rearrangement for Enantioselective O-Aryl Amination. Org. Lett..

[17] Körber K., Tang W., Hu X., Zhang X. (2002). A Practical Synthesis of 2-Amino-2′-hydroxy-1,1′-binaphthyl (NOBIN). Tetrahedron Lett..

[18] Smrcina M., Lorenc M., Hanuš V., Kocovsky P. (1991). A Facile Synthesis of 2-Amino-2?-hydroxy-1,1?-binaphthyl and 2,2?-Diamino-1,1?-binaphthyl by Oxidative Coupling Using Copper(II) Chloride. Synlett.

[19] Smrcina M., Lorenc M., Hanus V., Sedmera P., Kocovsky P. (1992). Synthesis of enantiomerically pure 2,2’-dihydroxy-1,1’-binaphthyl, 2,2’-diamino-1,1’-binaphthyl, and 2-amino-2’-hydroxy-1,1’-binaphthyl. Comparison of processes operating as diastereoselective crystallization and as second order asymmetric transformation. J. Org. Chem..

[20] Smrcina M., Polakova J., Vyskocil S., Kocovsky P. (1993). Synthesis of enantiomerically pure binaphthyl derivatives. Mechanism of the enantioselective, oxidative coupling of naphthols and designing a catalytic cycle. J. Org. Chem..

[21] Smrcina M., Vyskocil S., Maca B., Polasek M., Claxton T.A., Abbott A.P., Kocovsky P. (1994). Selective Cross-Coupling of 2-Naphthol and 2-Naphthylamine Derivatives. A Facile Synthesis of 2,2’,3-Trisubstituted and 2,2’,3,3’-Tetrasubstituted 1,1’-Binaphthyls. J. Org. Chem..

[22] Ding K., Xu Q., Wang Y., Liu J., Yu Z., Du B., Wu Y., Koshima H., Matsuura T. (1997). Novel two-phase oxidative cross-coupling of the two-component molecular crystal of 2-naphthol and 2-naphthylamine. Chem. Commun..

[23] Singer R.A., Brock J.R., Carreira E.M. (2003). Synthesis of A Tridentate Ligand for Use in TiIV-Catalyzed Acetate Aldol Addition Reactions. Helv. Chim. Acta.

[24] Zhao X.-J., Li Z.-H., Ding T.-M., Tian J.-M., Tu Y.-Q., Wang A.-F., Xie Y.-Y. (2021). Enantioselective Synthesis of 3,3′-Disubstituted 2-Amino-2′-hydroxy-1,1′-binaphthyls by Copper-Catalyzed Aerobic Oxidative Cross-Coupling. Angew. Chem. Int. Ed..

[25] Zhang J.-W., Jiang F., Chen Y.-H., Xiang S.-H., Tan B. (2021). Synthesis of Structurally Diversified BINOLs and NOBINs via Palladium-Catalyzed C-H Arylation with Diazoquinones. Sci. China Chem..

[26] Sheradsky T., Avramovici-Grisaru S. (1980). Hydrolytic cleavages of 7-nitro-2-phenyl-1,2-benzisoxazol-3-one. Evidence for the occurrence of a benzidine-like rearrangement of N,O-diphenylhydroxylamines. J. Heterocycl. Chem..

[27] Gao H., Ess D.H., Yousufuddin M., Kürti L. (2013). Transition-Metal-Free Direct Arylation: Synthesis of Halogenated 2-Amino-2′-hydroxy-1,1′-biaryls and Mechanism by DFT Calculations. J. Am. Chem. Soc..

[28] De C.K., Pesciaioli F., List B. (2013). Catalytic Asymmetric Benzidine Rearrangement. Angew. Chem. Int. Ed..

[29] Li G.-Q., Gao H., Keene C., Devonas M., Ess D.H., Kürti L. (2013). Organocatalytic Aryl-Aryl Bond Formation: An Atro-poselective [3,3]-Rearrangement Approach to BINAM Derivatives. J. Am. Chem. Soc..

[30] Yanagi T., Otsuka S., Kasuga Y., Fujimoto K., Murakami K., Nogi K., Yorimitsu H., Osuka A. (2016). Metal-Free Approach to Biaryls from Phenols and Aryl Sulfoxides by Temporarily Sulfur-Tethered Regioselective C–H/C–H Coupling. J. Am. Chem. Soc..

[31] Hori M., Guo J.-D., Yanagi T., Nogi K., Sasamori T., Yorimitsu H. (2018). Sigmatropic Rearrangements of Hyperva-lent-Iodine-Tethered Intermediates for the Synthesis of Biaryls. Angew. Chem. Int. Ed..

[32] Guo L., Liu F., Wang L., Yuan H., Feng L., Kürti L., Gao H. (2019). Cascade Approach to Highly Functionalized Biaryls by a Nucleophilic Aromatic Substitution with Arylhydroxylamines. Org. Lett..

[33] Yuan H., Du Y., Liu F., Guo L., Sun Q., Feng L., Gao H. (2020). Tandem Approach to NOBIN Analogues from Arylhydrox-ylamines and Diaryliodonium Salts via [3,3]-Sigmatropic Rearrangement. Chem. Commun..

[34] Zhang J., Qi L., Li S., Xiang S., Tan B. (2020). Direct Construction of NOBINs via Domino Arylation and Sigmatropic Rearrangement Reactions. Chin. J. Chem..

[35] Deprez N.R., Sanford M.S. (2007). Reactions of Hypervalent Iodine Reagents with Palladium: Mechanisms and Applications in Organic Synthesis. Inorg. Chem..

[36] Phipps R.J., Gaunt M. (2009). A Meta-Selective Copper-Catalyzed C-H Bond Arylation. Science.

[37] Duong H.A., Gilligan R.E., Cooke M.L., Phipps R.J., Gaunt M. (2010). Copper(II)-Catalyzed meta-Selective Direct Arylation of α-Aryl Carbonyl Compounds. Angew. Chem. Int. Ed..

[38] Ghosh M.K., Rzymkowski J., Kalek M. (2019). Transition-Metal-Free Aryl-Aryl Cross-Coupling: C-H Arylation of 2-Naphthols with Diaryliodonium Salts. Chem. Eur. J..

[39] Phipps R.J., Grimster N.P., Gaunt M. (2008). Cu(II)-Catalyzed Direct and Site-Selective Arylation of Indoles Under Mild Conditions. J. Am. Chem. Soc..

[40] Aradi K., Tóth B.L., Tolnai G.L., Novák Z. (2016). Diaryliodonium Salts in Organic Syntheses: A Useful Compound Class for Novel Arylation Strategies. Synlett.

[41] Qi L.-W., Mao J.-H., Zhang J., Tan B. (2017). Organocatalytic asymmetric arylation of indoles enabled by azo groups. Nat. Chem..

[42] Qi L.-W., Li S., Xiang S.-H., Wang J.J., Tan B. (2019). Asymmetric construction of atropisomeric biaryls via a redox neutral cross-coupling strategy. Nat. Catal..

[43] Ding W.-Y., Yu P., An Q.-J., Bay K.L., Xiang S.-H., Li S., Chen Y., Houk K.N., Tan B. (2020). DFT-Guided Phosphor-ic-Acid-Catalyzed Atroposelective Arene Functionalization of Nitrosonaphthalene. Chemistry.

[44] Yan S., Xia W., Li S., Song Q., Xiang S.-H., Tan B. (2020). Michael Reaction Inspired Atroposelective Construction of Axially Chiral Biaryls. J. Am. Chem. Soc..

[45] Phipps R.J., McMurray L., Ritter S., Duong H.A., Gaunt M.J. (2012). Copper-Catalyzed Alkene Arylation with Diaryliodonium Salts. J. Am. Chem. Soc..

[46] Allen A.E., Macmillan D.W.C. (2011). Enantioselective α-Arylation of Aldehydes via the Productive Merger of Iodonium Salts and Organocatalysis. J. Am. Chem. Soc..

[47] Harvey J.S., Simonovich S.P., Jamison C.R., Macmillan D.W.C. (2011). Enantioselective α-Arylation of Carbonyls via Cu(I)-Bisoxazoline Catalysis. J. Am. Chem. Soc..

[48] Zhu S., MacMillan D.W.C. (2012). Enantioselective Copper-Catalyzed Construction of Aryl Pyrroloindolines via an Aryla-tion-Cyclization Cascade. J. Am. Chem. Soc..

[49] Ribas X., Jackson D.A., Donnadieu B., Mahía J., Parella T., Xifra R., Hedman B., Hodgson K.O., Llobet A., Stack T.D.P. (2002). Aryl C-H Activation by CuII To Form an Organometallic Aryl-CuIII Species: A Novel Twist on Copper Disproportionation. Angew. Chem. Int. Ed..

[50] Yao B., Wang D.-X., Huang Z.-T., Wang M.-X. (2009). Room-temperature aerobic formation of a stable aryl-Cu(III) complex and its reactions with nucleophiles: Highly efficient and diverse arene C-H functionalizations of azacalix[1]arene[3]pyridine. Chem. Commun..

[51] Ribas X., Calle C., Poater A., Casitas A., Gómez L., Xifra R., Parella T., Benet-Buchholz J., Schweiger A., Mitrikas G. (2010). Facile C-H Bond Cleavage via a Proton-Coupled Electron Transfer Involving a C-H···CuII Interaction. J. Am. Chem. Soc..

