# Supplementary material for: Copper-Catalyzed Synthesis of Axially Chiral Biaryls with Diaryliodonium Salts as Arylation Reagents
Source: Molecules. 2021 May 27;26(11):3223. doi: 10.3390/molecules26113223 (PMC8199266; doi:10.3390/molecules26113223)
Supplement: Supplementary file 1 [file molecules-26-03223-s001.zip › molecules-1234673-supplementary.pdf]

Supplementary Materials

Copper-Catalyzed Synthesis of Axially Chiral Biaryls with Diaryliodonium Salts as Arylation Reagents

Ji-Wei Zhang[1], Shao-Hua Xiang[1,2]*, Shaoyu Li[1,2] and Bin Tan[1]*

1. Shenzhen Key Laboratory of Small Molecule Drug Discovery and Synthesis, Department of Chemistry, Southern University of Science and Technology, Shenzhen, Guangdong 518055, China;
2. Academy for Advanced Interdisciplinary Studies, Southern University of Science and Technology, Shenzhen, Guangdong 518055, China.

**email:* *tanb@sustech.edu.cn; xiangsh@sustech.edu.cn*

Table of Contents

I. General Information--2

II. Supplementary Tables for the Optimization of Reaction Conditions--------------------------------3

III. Supplementary Experimental Procedures--5

1. General procedures for synthesis of NOBIN derivatives **3**--------------------------------------5

2. General procedures for synthesis of BINAM derivatives **5**-----------------------------------14

IV. Control Experiments --16

V. Copies of NMR Spectra---17

I. General information

Reagents were purchased at the highest commercial quality and used without further purification, unless otherwise stated. Analytical thin layer chromatography (TLC) was performed on precoated silica gel 60 F254 plates. Flash column chromatography was performed using Tsingdao silica gel (60, particle size 0.040-0.063 mm). Visualization on TLC was achieved by use of UV light (254 nm). NMR spectra were recorded on a Bruker DPX 400 spectrometer at 400 MHz for ^{1}H NMR, 100 MHz for ^{13}C NMR and 376 MHz for ^{19}F NMR in $CDCl_3$ or Acetone-d_6 or DMSO-d_6 with tetramethylsilane (TMS) as internal standard. Chemical shifts are reported in ppm and coupling constants are given in Hz. Data for ^{1}H NMR are recorded as follows: chemical shift (ppm), multiplicity (s, singlet; d, doublet; t, triplet; q, quartet; m, multiplet), coupling constant (Hz), integration. Data for ^{13}C NMR are reported in terms of chemical shift (δ, ppm). High resolution mass spectra (HRMS) were recorded on a LC-TOF spectrometer (Micromass).

II. Supplementary Tables for the Reaction Condition Optimizations

Table S1. Solvent and catalysts screenings for the reaction with *N*-naphthylhydroxylamine[a]

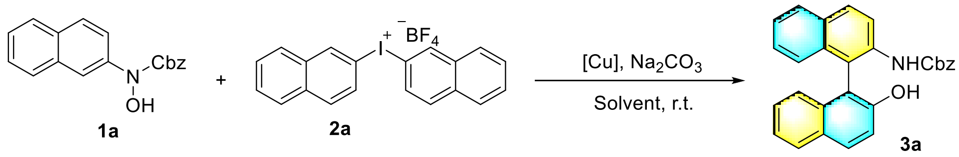

Entry	[Cu]	Solvent	Yield (%)[b]
1[c]	Cu(TFA)$_2$	DCM	96
2	Cu(TFA)$_2$	DCM	98
3	Cu(TFA)$_2$	DCE	92
4	Cu(TFA)$_2$	toluene	80
5	Cu(TFA)$_2$	EA	86
6	Cu(TFA)$_2$	THF	90
7	Cu(TFA)$_2$	MeCN	88
8	Cu(OTf)$_2$	DCM	82
9	Cu(OAc)$_2$	DCM	89
10	Cu(OTf)	DCM	89
11	CuI	DCM	86

[a]All reactions were performed with [Cu] (10 mol%), **1a** (0.10 mmol), **2a** (0.12 mmol) and Na_2CO_3 (0.13 mmol) in solvent (2.0 mL) at room temperature; [b]Yield was determined by ^{1}H-NMR analysis of the crude reaction mixture using 1,3,5-trimethoxybenzene as the internal standard. [c]**2a** (1.0 equiv) and base (0.12 mmol) were used.

Table S2. Base and loading screenings for the reaction with *N*-naphthylhydroxylamine[a]

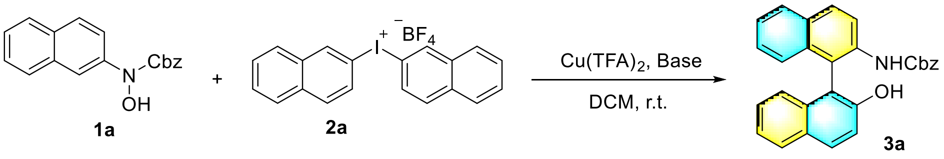

Entry	[M]	Base	Solvent	Yield (%)[b]
1	$Cu(TFA)_2$	K_2CO_3	DCM	93
2	$Cu(TFA)_2$	Cs_2CO_3	DCM	90
3	$Cu(TFA)_2$	NaOH	DCM	75
4	$Cu(TFA)_2$	NaO^{t}Bu	DCM	66
5	$Cu(TFA)_2$	Et_3N	DCM	73
6	$Cu(TFA)_2$	pyridine	DCM	trace
7	$Cu(TFA)_2$	—	DCM	30
8[c]	$Cu(TFA)_2$	Na_2CO_3	DCM	90
9[d]	$Cu(TFA)_2$	Na_2CO_3	DCM	83

[a]Unless otherwise specified, reactions were performed with $Cu(TFA)_2$ (10 mol%), **1a** (0.10 mmol), **2a** (0.12 mmol) and base (0.13 mmol) in DCM (2.0 mL) at room temperature; [b]Yield was determined by ^{1}H-NMR analysis of the crude reaction mixture using 1,3,5-trimethoxybenzene as the internal standard; [c]$Cu(TFA)_2$ (5 mol%) was used; [d]$Cu(TFA)_2$ (3 mol%) was used.

III. Supplementary Experimental Procedures

General procedures for synthesis of NOBIN derivatives 3

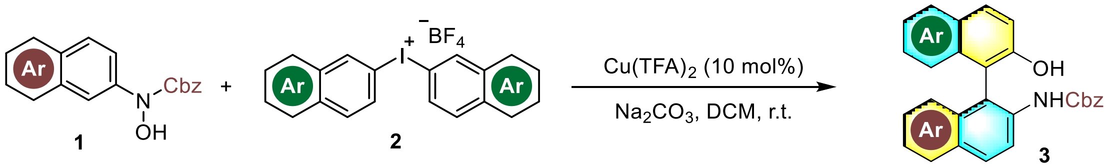

1 (0.20 mmol), **2** (0.24 mmol), Na_2CO_3 (27.6 mg, 0.26 mmol) and $Cu(TFA)_2$ (5.8 mg, 10 mol%) were added to a bottle with a magnetic stirring bar. DCM (4.0 mL) was added and the reaction mixture was stirred at room temperature till **1** was completely consumed (monitored by TLC). After evaporated the solvent, the residue was purified by flash chromatography eluted with DCM to afford the corresponding product **3**.

Benzyl (2'-hydroxy-[1,1'-binaphthalen]-2-yl)carbamate (3a)

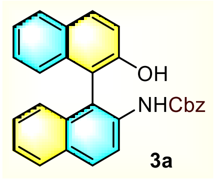

Yield: 98%. **^{1}H NMR (400 MHz, $CDCl_3$)** δ 8.56 (d, *J* = 9.6 Hz, 1H), 8.08 (d, *J* = 9.2 Hz, 1H), 8.00 (d, *J* = 9.2 Hz, 1H), 7.94 (t, *J* = 7.2 Hz, 2H), 7.47-7.27 (m, 10H), 7.15 (d, *J* = 8.8 Hz, 1H), 7.05 (d, *J* = 8.8 Hz, 1H), 6.54 (s, 1H), 5.21 (s, 1H), 5.07 (s, 2H). **^{13}C NMR (100 MHz, $CDCl_3$)** δ 153.6, 152.0, 135.9, 135.7, 133.2, 132.9, 131.3, 130.8, 130.4, 129.4, 128.6, 128.5, 128.4, 128.3, 128.3, 127.5, 127.4, 125.3, 125.1, 124.1, 124.0, 119.7, 117.9, 116.7, 112.7, 67.1. **HRMS (ESI)** calcd for [M+H] $C_{28}H_{22}NO_3$, m/z: 420.1594, found: 420.1595.

methyl (2'-hydroxy-[1,1'-binaphthalen]-2-yl)carbamate (3b)

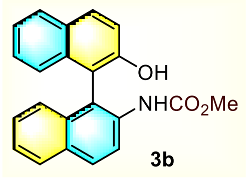

Yield: 91%. **^{1}H NMR (400 MHz, $CDCl_3$)** δ 8.37 (d, *J* = 9.2 Hz, 1H), 7.89 (d, *J* = 8.8 Hz, 1H), 7.84 (d, *J* = 9.2 Hz, 1H), 7.77 (d, *J* = 8.0 Hz, 2H), 7.30-7.22 (m, 3H), 7.16-7.12 (m, 2H), 7.00 (d, *J* = 8.4 Hz, 1H), 6.89 (d, *J* = 8.0 Hz, 1H), 6.34 (s, 1H), 5.30 (s, 1H), 3.39 (s, 3H). **^{13}C NMR (100 MHz, $CDCl_3$)** δ 154.0, 152.1, 135.9, 133.2, 133.0, 131.2, 130.7, 130.3, 129.4, 128.4, 128.3, 127.5, 127.3, 125.1, 125.1, 124.1, 124.0, 119.3, 118.0, 116.5, 112.7, 52.3. **HRMS (ESI)** calcd for [M+H] $C_{22}H_{18}NO_3$, m/z: 344.1281, found: 344.1281.

benzyl (2'-hydroxy-6-methoxy-[1,1'-binaphthalen]-2-yl)carbamate (3c)

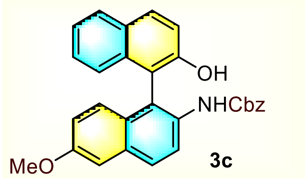

Yield: 94%. **^{1}H NMR (400 MHz, $CDCl_3$)** δ 8.45 (d, *J* = 8.8 Hz, 1H), 7.98 (d, *J* = 8.8 Hz, 1H), 7.96 (d, *J* = 9.6 Hz, 1H), 7.92 (dd, *J* = 8.4, 1.2 Hz, 1H), 7.42-7.26 (m, 8H), 7.24 (d, *J* = 2.4 Hz, 1H), 7.08 (d, *J* = 9.2 Hz, 1H), 7.06 (d, *J* = 8.4 Hz, 1H), 6.99 (dd, *J* = 9.2, 2.4 Hz, 1H), 6.48 (s, 1H), 5.45 (s, 1H), 5.07 (d, *J* = 12.4 Hz, 1H), 5.03 (d, *J* = 12.4 Hz, 1H), 3.92 (s, 3H). **^{13}C NMR (100 MHz, $CDCl_3$)** δ 157.3, 153.8, 152.0, 135.8, 133.8, 133.3, 132.1, 131.2, 129.4,

129.0, 128.6, 128.4, 128.3, 128.3, 128.3, 127.4, 126.8, 124.2, 123.9, 120.8, 119.9, 118.0, 117.9, 113.0, 106.5, 67.1, 55.4. **HRMS (ESI)** calcd for [M+H] $C_{29}H_{24}NO_4$, m/z: 450.1700, found: 450.1697.

benzyl (2'-hydroxy-6-phenyl-[1,1'-binaphthalen]-2-yl)carbamate (3d)

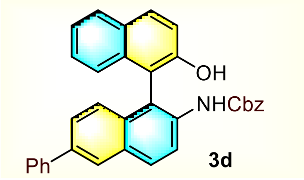

Yield: 93%. **^{1}H NMR (400 MHz, CDCl$_3$)** δ 8.48 (d, *J* = 9.2 Hz, 1H), 8.05 (d, *J* = 2.0 Hz, 1H), 8.02 (d, *J* = 9.2 Hz, 1H), 7.92 (d, *J* = 8.8 Hz, 1H), 7.86 (d, *J* = 7.6 Hz, 1H), 7.61 (dd, *J* = 7.6, 2.4 Hz, 2H), 7.49 (dd, *J* = 8.8, 2.0 Hz, 1H), 7.41 (t, *J* = 7.6 Hz, 2H), 7.35-7.30 (m, 3H), 7.27-7.21 (m, 4H), 7.19-7.14 (m, 3H), 7.02 (d, *J* = 8.4 Hz, 1H), 6.52 (s, 1H), 5.46 (s, 1H), 4.98 (d, *J* = 12.4 Hz, 1H), 4.93 (d, *J* = 12.4 Hz, 1H). **^{13}C NMR (100 MHz, CDCl$_3$)** δ 153.7, 152.2, 140.7, 138.0, 135.9, 135.7, 133.3, 132.2, 131.3, 131.1, 130.6, 129.5, 129.0, 128.6, 128.5, 128.4, 128.3, 127.5, 127.5, 127.3, 127.0, 126.1, 125.8, 124.2, 124.1, 120.2, 118.1, 117.1, 112.7, 67.2. **HRMS (ESI)** calcd for [M+H] $C_{34}H_{26}NO_3$, m/z: 496.1907, found: 496.1909.

benzyl (6-bromo-2'-hydroxy-[1,1'-binaphthalen]-2-yl)carbamate (3e)

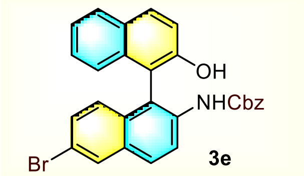

Yield: 96%. **^{1}H NMR (400 MHz, CDCl$_3$)** δ 8.54 (d, *J* = 9.2 Hz, 1H), 8.09 (d, *J* = 2.0 Hz, 1H), 7.99 (d, *J* = 9.2 Hz, 1H), 7.94 (d, *J* = 8.8 Hz, 1H), 7.94 (d, *J* = 8.0 Hz, 1H), 7.43-7.25 (m, 9H), 7.02 (d, *J* = 9.2 Hz, 1H), 7.01 (d, *J* = 8.4 Hz, 1H), 6.57 (s, 1H), 5.60 (s, 1H), 5.05 (d, *J* = 12.0 Hz, 1H), 5.01 (d, *J* = 12.0 Hz, 1H). **^{13}C NMR (100 MHz, CDCl$_3$)** δ 153.6, 152.1, 136.1, 135.6, 133.2, 131.9, 131.6, 131.5, 130.6, 130.2, 129.4, 129.2, 128.6, 128.5, 128.4, 128.3, 127.6, 127.1, 124.1, 124.0, 120.9, 119.3, 118.1, 117.6, 112.2, 67.3. **HRMS (ESI)** calcd for [M+H] $C_{28}H_{21}BrNO_3$, m/z: 498.0700, found: 498.0700.

benzyl (6-fluoro-2'-hydroxy-[1,1'-binaphthalen]-2-yl)carbamate (3f)

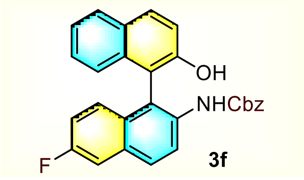

Yield: 92%. **^{1}H NMR (400 MHz, CDCl$_3$)** δ 8.44 (d, *J* = 9.2 Hz, 1H), 7.91 (dd, *J* = 9.2, 2.8 Hz, 2H), 7.85 (d, *J* = 8.0 Hz, 1H), 7.47 (dd, *J* = 9.2, 2.8 Hz, 1H), 7.35-7.18 (m, 8H), 7.07 (dd, *J* = 9.3, 5.6 Hz, 1H), 7.00 (td, *J* = 8.8, 2.8 Hz, 1H), 6.94 (d, *J* = 8.4 Hz, 1H), 6.45 (s, 1H), 5.46 (s, 1H), 4.99 (d, *J* = 12.4 Hz, 1H), 4.94 (d, *J* = 12.4 Hz, 1H). **^{13}C NMR (100 MHz, CDCl$_3$)** δ 160.3 (d, *J* = 245.0 Hz), 153.8, 152.1, 135.7, 135.1 (d, *J* = 2.0 Hz), 133.2, 131.6 (d, *J* = 9.0 Hz), 131.4, 130.0, 129.4, 129.4, 129.4, 128.6, 128.5, 128.4, 128.3, 127.8 (d, *J* = 9.0 Hz), 127.6, 124.0 (d, *J* = 6.0 Hz), 121.2, 118.1, 117.9, 117.5 (d, *J* = 25.0 Hz) 112.5, 111.4 (d, *J* = 21.0 Hz), 67.2. **^{19}F NMR (100 MHz, CDCl$_3$)** δ -115.88. **HRMS (ESI)** calcd for [M+H] $C_{28}H_{21}FNO_3$, m/z: 438.1500, found: 438.1500

methyl 2-(((benzyloxy)carbonyl)amino)-2'-hydroxy-[1,1'-binaphthalene]-6-carboxylate (3g)

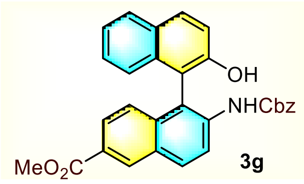

Yield: 93%. **^{1}H NMR (400 MHz, $CDCl_3$)** δ 8.62 (d, *J* = 9.2 Hz, 1H), 8.41 (d, *J* = 2.0 Hz, 1H), 8.03 (d, *J* = 9.2 Hz, 1H), 8.01 (d, *J* = 8.8 Hz, 1H), 7.93 (d, *J* = 7.6 Hz, 1H), 7.75 (dd, *J* = 9.2, 2.0 Hz, 1H), 7.44 (d, *J* = 9.2 Hz, 1H), 7.40 (t, *J* = 8.0 Hz, 1H), 7.35-7.26 (m, 6H), 7.16 (d, *J* = 8.8 Hz, 1H), 6.98 (d, *J* = 8.0 Hz, 1H), 6.70 (s, 1H), 6.27 (s, 1H), 5.07 (d, *J* = 12.4 Hz, 1H), 5.03 (d, *J* = 12.4 Hz, 1H), 3.86 (s, 3H). **^{13}C NMR (100 MHz, $CDCl_3$)** δ 167.1, 153.4, 152.6, 138.0, 135.6, 135.5, 133.2, 131.5, 131.5, 131.2, 129.5, 129.4, 128.6, 128.5, 128.4, 128.4, 127.5, 126.5, 126.1, 125.4, 124.0, 123.9, 119.9, 118.5, 117.1, 112.0, 67.3, 52.3. **HRMS (ESI)** calcd for [M+H] $C_{30}H_{24}NO_5$, m/z: 478.1649, found: 478.1649.

benzyl (2'-hydroxy-7-methoxy-[1,1'-binaphthalen]-2-yl)carbamate (3h)

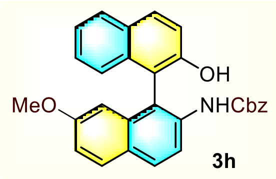

Yield: 97%. **^{1}H NMR (400 MHz, $CDCl_3$)** δ 8.39 (d, *J* = 8.8 Hz, 1H), 7.99 (d, *J* = 9.2 Hz, 1H), 7.98 (d, *J* = 9.2 Hz, 1H), 7.93 (d, *J* = 8.0 Hz, 1H), 7.83 (d, *J* = 8.8 Hz, 1H), 7.42-7.39 (m, 2H), 7.37-7.26 (m, 6H), 7.14-7.10 (m, 2H), 6.54 (s, 1H), 6.45 (d, *J* = 2.8 Hz, 1H), 5.48 (s, 1H), 5.08 (d, *J* = 12.4 Hz, 1H), 5.04 (d, *J* = 12.0 Hz, 1H), 3.51 (s, 3H). **^{13}C NMR (100 MHz, $CDCl_3$)** δ 158.9, 153.6, 152.1, 136.4, 135.8, 134.4, 133.0, 131.3, 130.0, 129.9, 129.4, 128.6, 128.5, 128.3, 128.3, 127.4, 126.3, 124.2, 124.0, 118.0, 117.5, 117.3, 115.9, 112.8, 103.9, 67.1, 55.1. **HRMS (ESI)** calcd for [M+H] $C_{29}H_{24}NO_4$, m/z: 450.1700, found: 450.1699.

benzyl (2'-hydroxy-7-phenyl-[1,1'-binaphthalen]-2-yl)carbamate (3i)

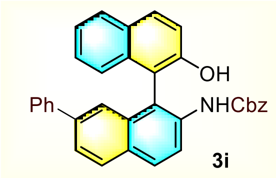

Yield: 98%. **^{1}H NMR (400 MHz, $CDCl_3$)** δ 8.60 (d, *J* = 8.8 Hz, 1H), 8.12 (d, *J* = 9.2 Hz, 1H), 8.04 (d, *J* = 8.4 Hz, 1H), 8.03 (d, *J* = 8.8 Hz, 1H), 7.97 (d, *J* = 8.0 Hz, 1H), 7.76 (d, *J* = 8.4 Hz, 1H), 7.48-7.30 (m, 14H), 7.19 (dd, *J* = 8.4, 2.0 Hz, 1H), 6.65 (s, 1H), 5.67 (s, 1H), 5.12 (d, *J* = 12.0 Hz, 1H), 5.08 (d, *J* = 12.4 Hz, 1H). **^{13}C NMR (100 MHz, $CDCl_3$)** δ 153.7, 152.3, 141.0, 140.2, 136.3, 135.8, 133.4, 133.3, 131.4, 130.1, 130.0, 129.5, 128.9, 128.8, 128.6, 128.6, 128.4, 128.3, 127.5, 127.5, 127.5, 125.2, 124.2, 124.1, 123.1, 119.9, 118.1, 117.6, 112.7, 67.2. **HRMS (ESI)** calcd for [M+H] $C_{34}H_{26}NO_3$, m/z: 496.1907, found: 496.1907.

benzyl (7-bromo-2'-hydroxy-[1,1'-binaphthalen]-2-yl)carbamate (3j)

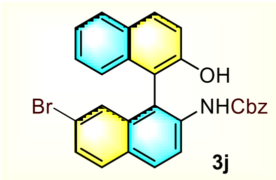

Yield: 95%. **^{1}H NMR (400 MHz, $CDCl_3$)** δ 8.54 (d, *J* = 9.2 Hz, 1H), 8.00 (dd, *J* = 8.8, 3.2 Hz, 2H), 7.94 (d, *J* = 8.0 Hz, 1H), 7.78 (d, *J* = 8.4 Hz, 1H), 7.52 (dd, *J* = 8.4, 2.0 Hz, 1H), 7.44-7.25 (m, 9H), 7.02 (d, *J* = 8.4 Hz, 1H), 6.54 (s, 1H), 5.47 (s, 1H), 5.06 (d, *J* = 12.4 Hz, 1H), 5.02 (d, *J* = 12.4 Hz, 1H). **^{13}C NMR (100 MHz, $CDCl_3$)** δ 153.5, 152.1, 136.7, 135.6, 134.3, 133.0, 131.6, 130.2, 130.0, 129.5, 129.2, 128.7, 128.6, 128.6, 128.4, 128.3, 127.7, 127.1, 124.1, 123.9,

122.0, 120.0, 118.1, 116.4, 111.9, 67.3. **HRMS (ESI)** calcd for [M+H] $C_{28}H_{21}BrNO_3$, m/z: 498.0700, found: 498.0699.

benzyl (7'-bromo-2'-hydroxy-[1,1'-binaphthalen]-2-yl)carbamate (3k)

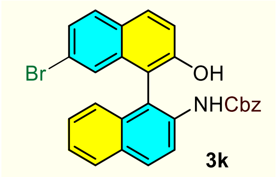

Yield: 94%. **^{1}H NMR (400 MHz, $CDCl_3$)** δ 8.52 (d, *J* = 8.8 Hz, 1H), 8.08 (d, *J* = 9.2 Hz, 1H), 7.95 (d, *J* = 8.0 Hz, 1H), 7.94 (d, *J* = 8.8 Hz, 1H), 7.77 (d, *J* = 8.8 Hz, 1H), 7.49-7.45 (m, 2H), 7.39 (d, *J* = 8.8 Hz, 1H), 7.36-7.28 (m, 6H), 7.19 (d, *J* = 2.4 Hz, 1H), 7.11 (d, *J* = 8.8 Hz, 1H), 6.48 (s, 1H), 5.49 (s, 1H), 5.09 (d, *J* = 12.4 Hz, 1H), 5.05 (d, *J* = 12.0 Hz, 1H). **^{13}C NMR (100 MHz, $CDCl_3$)** δ 153.6, 153.0, 135.9, 135.6, 134.6, 132.8, 131.2, 130.9, 130.7, 130.1, 128.6, 128.4, 128.4, 128.4, 127.8, 127.6, 127.5, 126.1, 125.4, 124.9, 122.1, 119.9, 118.5, 116.2, 112.2, 67.3. **HRMS (ESI)** calcd for [M+H] $C_{28}H_{21}BrNO_3$, m/z: 498.0700, found: 498.0701.

methyl 2'-(((benzyloxy)carbonyl)amino)-2-hydroxy-[1,1'-binaphthalene]-6-carboxylate (3l)

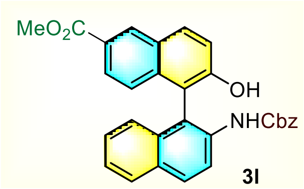

Yield: 90%. **^{1}H NMR (400 MHz, Acetone-*d*$_6$)** δ 8.96 (s, 1H), 8.62 (d, *J* = 2.0 Hz, 1H), 8.44 (d, *J* = 9.2 Hz, 1H), 8.15 (d, *J* = 8.0 Hz, 1H), 8.06 (d, *J* = 8.8 Hz, 1H), 7.97 (d, *J* = 8.4 Hz, 1H), 7.78 (dd, *J* = 8.8, 2.0 Hz, 1H), 7.50 (d, *J* = 9.2 Hz, 1H), 7.43-7.39 (m, 2H), 7.29-7.20 (m, 6H), 7.08 (d, *J* = 8.4 Hz, 1H), 7.04 (d, *J* = 8.8 Hz, 1H), 5.04 (s, 2H), 3.89 (s, 3H). **^{13}C NMR (100 MHz, Acetone-*d*$_6$)** δ 171.7, 161.1, 158.9, 141.9, 141.7, 140.8, 138.4, 137.3, 136.4, 136.1, 134.1, 134.1, 133.5, 133.3, 133.3, 133.1, 133.1, 131.7, 131.2, 130.4, 130.1, 129.9, 129.5, 125.8, 124.9, 119.2, 71.3, 56.6. **HRMS (ESI)** calcd for [M+H] $C_{30}H_{24}NO_5$, m/z: 478.1649, found: 478.1651.

dimethyl 2-(((benzyloxy)carbonyl)amino)-2'-hydroxy-[1,1'-binaphthalene]-6,6'-dicarboxylate (3m)

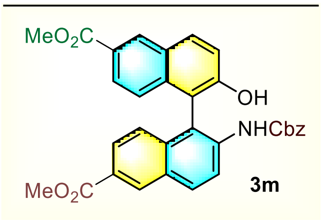

Yield: 81%. **^{1}H NMR (400 MHz, $CDCl_3$)** δ 8.58 (d, *J* = 1.6 Hz, 1H), 8.56 (d, *J* = 8.8 Hz, 1H), 8.39 (d, *J* = 2.0 Hz, 1H), 8.05 (d, *J* = 8.8 Hz, 1H), 8.01 (d, *J* = 9.2 Hz, 1H), 7.78 (dd, *J* = 8.8, 1.6 Hz, 1H), 7.72 (dd, *J* = 8.8, 1.6 Hz, 1H), 7.43 (d, *J* = 9.2 Hz, 1H), 7.31-7.26 (m, 3H), 7.24-7.21 (m, 2H), 7.05 (d, *J* = 8.8 Hz, 1H), 6.94 (d, *J* = 8.8 Hz, 1H), 6.60 (s, 1H), 6.34 (s, 1H), 5.02 (s, 2H), 3.91 (s, 3H), 3.84 (s, 3H). **^{13}C NMR (100 MHz, $CDCl_3$)** δ 167.1, 167.0, 154.6, 153.3, 138.1, 135.7, 135.4, 135.3, 133.0, 131.8, 131.6, 131.2, 129.6, 128.6, 128.5, 128.4, 128.4, 127.0, 126.6, 126.3, 125.6, 125.1, 124.0, 120.0, 119.3, 116.3, 112.3, 67.4, 52.3, 52.2. **HRMS (ESI)** calcd for [M+H] $C_{32}H_{26}NO_7$, m/z: 536.1704, found: 536.1706.

benzyl (6,6'-dibromo-2'-hydroxy-[1,1'-binaphthalen]-2-yl)carbamate (3n)

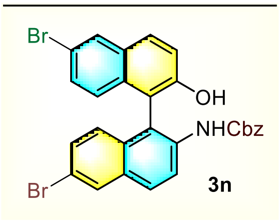

Yield: 90%. ^{1}**H NMR (400 MHz, Acetone-d_6)** δ 8.74 (s, 1H), 8.48 (d, J = 9.2 Hz, 1H), 8.19 (d, J = 2.4 Hz, 1H), 8.12 (d, J = 2.0 Hz, 1H), 8.04 (d, J = 9.2 Hz, 1H), 7.97 (d, J = 8.8 Hz, 1H), 7.44 (d, J = 8.8 Hz, 1H), 7.43 (s, 1H), 7.38 (dd, J = 9.2, 2.4 Hz, 1H), 7.34 (dd, J = 8.8, 2.0 Hz, 1H), 7.32-7.26 (m, 3H), 7.23-7.21 (m, 2H), 6.99 (d, J = 8.8 Hz, 1H), 6.86 (d, J = 8.8 Hz, 1H), 5.06 (d, J = 12.4 Hz, 1H), 5.03 (d, J = 12.4 Hz, 1H). ^{13}**C NMR (100 MHz, Acetone-d_6)** δ 154.1, 153.6, 136.6, 136.2, 132.5, 132.0, 131.8, 130.3, 130.2, 130.0, 130.0, 129.9, 129.6, 128.3, 128.1, 127.9, 127.9, 127.5, 126.1, 121.7, 120.5, 120.0, 118.0, 116.5, 113.4, 66.2. **HRMS (ESI)** calcd for [M+H] $C_{28}H_{20}Br_2NO_3$, m/z: 575.9805, found: 575.9808.

benzyl (7,7'-dibromo-2'-hydroxy-[1,1'-binaphthalen]-2-yl)carbamate (3o)

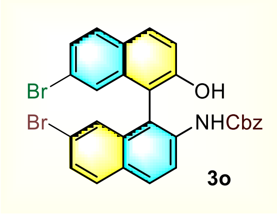

Yield: 85%. ^{1}**H NMR (400 MHz, CDCl$_3$)** δ 8.53 (dd, J = 9.2, 2.0 Hz, 1H), 8.02 (d, J = 9.2 Hz, 1H), 7.94 (d, J = 8.8 Hz, 1H), 7.79 (d, J = 8.8 Hz, 1H), 7.78 (d, J = 8.4 Hz, 1H), 7.53 (dd, J = 8.8, 2.0 Hz, 1H), 7.48 (dd, J = 8.8, 2.0 Hz, 1H), 7.38-7.32 (m, 4H), 7.29-7.26 (m, 2H), 7.22 (d, J = 2.0 Hz, 1H), 7.13 (d, J = 1.6 Hz, 1H), 6.44 (s, 1H), 5.49 (s, 1H), 5.08 (d, J = 12.4 Hz, 1H), 5.04 (d, J = 12.0 Hz, 1H). ^{13}**C NMR (100 MHz, CDCl$_3$)** δ 153.4, 153.0, 136.8, 135.5, 134.4, 134.1, 131.6, 130.5, 130.3, 130.1, 129.2, 128.9, 128.6, 128.5, 128.4, 127.9, 127.7, 126.8, 125.8, 122.4, 122.2, 120.1, 118.6, 115.4, 111.3, 67.4. **HRMS (ESI)** calcd for [M+H] $C_{28}H_{20}Br_2NO_3$, m/z: 575.9805, found: 575.9804.

benzyl (2'-hydroxy-7,7'-diphenyl-[1,1'-binaphthalen]-2-yl)carbamate (3p)

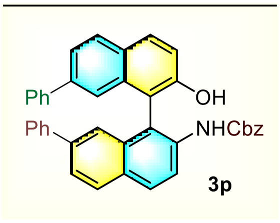

Yield: 83%. ^{1}**H NMR (400 MHz, CDCl$_3$)** δ 8.58 (d, J = 8.8 Hz, 1H), 8.12 (d, J = 8.8 Hz, 1H), 8.05-8.01 (m, 3H), 7.74 (dd, J = 8.4, 2.0 Hz, 1H), 7.69 (dd, J = 8.4, 2.0 Hz, 1H), 7.47-7.41 (m, 7H), 7.39-7.27 (m, 11H), 6.63 (s, 1H), 5.38 (s, 1H), 5.12 (d, J = 12.4 Hz, 1H), 5.09 (d, J = 12.0 Hz, 1H). ^{13}**C NMR (100 MHz, CDCl$_3$)** δ153.7, 152.5, 141.0, 141.0, 140.4, 140.3, 136.3, 135.7, 133.5, 133.2, 131.2, 130.2, 130.1, 129.1, 129.0, 128.8, 128.8, 128.7, 128.6, 128.4, 128.3, 127.5, 127.5, 127.4, 127.4, 125.2, 123.9, 122.9, 121.9, 120.0, 118.0, 117.2, 112.9, 67.2. **HRMS (ESI)** calcd for [M+H] $C_{40}H_{30}NO_3$, m/z: 572.2220, found: 572.2223.

methyl 2'-((benzyl carbonyl)amino)-2-hydroxy-6'-methoxy-[1,1'-binaphthalene]-6-carboxyl-ate (3q)

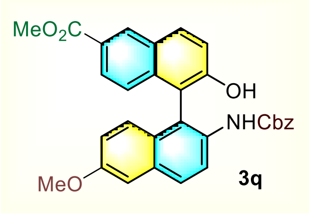

Yield: 88%. ^{1}**H NMR (400 MHz, CDCl$_3$)** δ 8.62 (d, J = 2.0 Hz, 1H), 8.37 (d, J = 8.8 Hz, 1H), 8.04 (d, J = 8.8 Hz, 1H), 7.93 (d, J = 9.2 Hz, 1H), 7.81 (dd, J = 9.2, 2.0 Hz, 1H), 7.42 (d, J = 8.8 Hz, 1H), 7.34-7.20 (m, 6H), 7.05 (d, J = 8.8 Hz, 1H), 7.00 (d, J = 9.2 Hz, 1H),

6.95 (dd, J = 9.2, 2.8 Hz, 1H), 6.45 (s, 1H), 5.99 (s, 1H), 5.05 (d, J = 12.0 Hz, 1H), 5.00 (d, J = 12.0 Hz, 1H), 3.94 (s, 3H), 3.88 (s, 3H). **^{13}C NMR (100 MHz, CDCl$_3$)** δ 167.2, 157.3, 154.3, 153.9, 135.8, 135.7, 133.7, 132.5, 132.2, 131.5, 129.1, 128.5, 128.3, 128.3, 128.2, 128.1, 126.8, 126.6, 125.4, 124.3, 121.0, 120.0, 119.1, 117.7, 113.4, 106.5, 67.1, 55.3, 52.2. **HRMS (ESI)** calcd for [M+H] $C_{31}H_{26}NO_6$, m/z: 508.1755, found: 508.1759.

benzyl (6'-fluoro-2'-hydroxy-6-methoxy-[1,1'-binaphthalen]-2-yl)carbamate (3r)

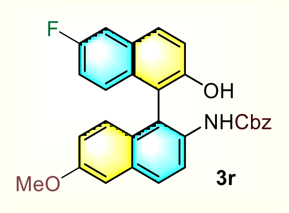

Yield: 85%. **^{1}H NMR (400 MHz, CDCl$_3$)** δ 8.40 (d, J = 8.8 Hz, 1H), 7.94 (d, J = 8.8 Hz, 1H), 7.89 (d, J = 8.8 Hz, 1H), 7.54 (dd, J = 9.5, 2.4 Hz, 1H), 7.40 (d, J = 8.8 Hz, 1H), 7.37-7.31 (m, 3H), 7.28-7.23 (m, 3H), 7.10-6.98 (m, 4H), 6.46 (s, 1H), 5.60 (s, 1H), 5.07 (d, J = 12.4 Hz, 1H), 5.01 (d, J = 12.4 Hz, 1H), 3.91 (s, 3H). **^{13}C NMR (100 MHz, CDCl$_3$)** δ 159.5 (d, J = 243.0 Hz), 157.3, 153.9, 151.5 (d, J = 2.0 Hz), 135.8, 133.6, 132.2, 130.2 (d, J = 5.0 Hz), 130.2, 129.9 (d, J = 9.0 Hz), 129.1, 128.6, 128.4, 128.3, 128.1, 120.9, 126.7, 126.5 (d, J = 8.0 Hz), 120.0, 119.3, 118.0, 117.4 (d, J = 25.0 Hz), 113.5, 111.6 (d, J = 21.0 Hz), 106.5, 67.2, 55.4. **^{19}F NMR (376 MHz, CDCl$_3$)** δ -118.10. **HRMS (ESI)** calcd for [M+H] $C_{29}H_{23}FNO_4$, m/z: 468.1606, found: 468.1607.

benzyl (6'-fluoro-2'-hydroxy-7-phenyl-[1,1'-binaphthalen]-2-yl)carbamate (3s)

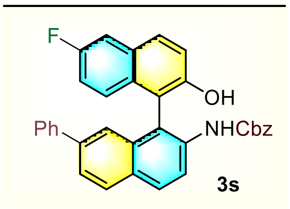

Yield: 84%. **^{1}H NMR (400 MHz, CDCl$_3$)** δ 8.55 (d, J = 9.2 Hz, 1H), 8.10 (d, J = 9.2 Hz, 1H), 8.02 (d, J = 8.8 Hz, 1H), 7.93 (d, J = 8.8 Hz, 1H), 7.75 (dd, J = 8.4, 2.0 Hz, 1H), 7.57 (dd, J = 9.6, 2.4 Hz, 1H), 7.47-7.44 (m, 3H), 7.41-7.28 (m, 9H), 7.16-7.08 (m, 2H), 6.57 (s, 1H), 5.55 (s, 1H), 5.12 (d, J = 12.0 Hz, 1H), 5.06 (d, J = 12.4 Hz, 1H). **^{13}C NMR (100 MHz, CDCl$_3$)** δ 159.6 (d, J = 242.0 Hz), 153.7, 151.7 (d, J = 2.0 Hz), 140.9, 140.3, 136.2, 135.7, 133.2, 130.5 (d, J = 5.0 Hz), 130.2, 130.1, 130.1, 130.0 (d, J = 10.0 Hz), 129.0, 128.8, 128.6, 128.5, 128.4, 127.5, 127.5, 126.5 (d, J = 8.0 Hz), 125.3, 122.9, 120.0, 119.4, 117.5 (d, J = 25.0 Hz), 117.3, 113.0, 111.8 (d, J = 21.0 Hz), 67.3. **^{19}F NMR (376 MHz, CDCl$_3$)** δ -117.88. **HRMS (ESI)** calcd for [M+H] $C_{34}H_{25}FNO_3$, m/z: 514.1813, found: 514.1814.

Methyl 2-(((benzyloxy)carbonyl)amino)-7'-bromo-2'-hydroxy-[1,1'-binaphthalene]-6-carb-oxylate (3t)

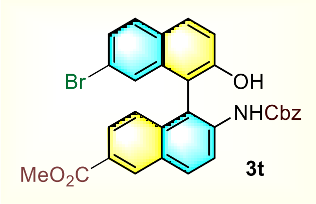

Yield: 86%. **^{1}H NMR (400 MHz, CDCl$_3$)** δ 8.61 (d, J = 9.2 Hz, 1H), 8.41 (d, J = 2.0 Hz, 1H), 8.04 (d, J = 9.2 Hz, 1H), 7.95 (d, J = 8.8 Hz, 1H), 7.77 (d, J = 8.8 Hz, 1H), 7.76 (dd, J = 8.8, 2.0 Hz, 1H), 7.46 (dd, J = 8.4, 2.0 Hz, 1H), 7.42 (d, J = 8.8 Hz, 1H), 7.37-7.27 (m, 5H), 7.09 (d, J = 8.8 Hz, 1H), 7.09 (d, J = 2.0 Hz, 1H), 6.60 (s, 1H), 6.29 (s, 1H), 5.09 (d, J = 12.0 Hz, 1H), 5.05 (d, J = 12.0 Hz, 1H) 3.87 (s, 3H). **^{13}C NMR (100 MHz, CDCl$_3$)** δ 167.1, 153.4, 153.3, 138.0, 135.4, 135.3, 134.5, 131.9, 131.5, 131.3, 130.2,

129.6, 128.6, 128.5, 128.4, 127.8, 127.5, 126.7, 126.3, 125.8, 125.1, 122.2, 119.9, 118.9, 116.1, 111.4, 67.4, 52.3. **HRMS (ESI)** calcd for [M+H] $C_{30}H_{23}BrNO_5$, m/z: 556.0754, found: 556.0756.

benzyl (7-bromo-2'-hydroxy-7'-phenyl-[1,1'-binaphthalen]-2-yl)carbamate (3u)

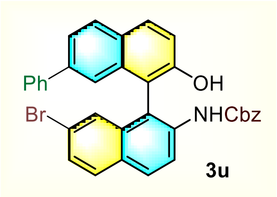

Yield: 88%. **^{1}H NMR (400 MHz, $CDCl_3$)** δ 8.50 (d, *J* = 9.2 Hz, 1H), 7.96 (d, *J* = 9.2 Hz, 2H), 7.95 (d, *J* = 8.4 Hz, 1H), 7.73 (d, *J* = 8.4 Hz, 1H), 7.62 (dd, *J* = 8.4, 1.6 Hz, 1H), 7.47 (dd, *J* = 8.8, 2.0 Hz, 1H), 7.38-7.18 (m, 12H), 7.13 (d, *J* = 2.0 Hz, 1H), 6.52 (s, 1H), 5.35 (s, 1H), 5.02 (d, *J* = 12.0 Hz, 1H), 4.99 (d, *J* = 12.4 Hz, 1H). **^{13}C NMR (100 MHz, $CDCl_3$)** δ 153.6, 152.5, 140.9, 140.5, 136.8, 135.6, 134.3, 133.3, 131.4, 130.3, 130.0, 129.3, 129.2, 128.8, 128.8, 128.7, 128.6, 128.4, 128.3, 127.5, 127.5, 127.1, 124.0, 122.1, 121.6, 120.1, 118.1, 116.2, 112.2, 67.3. **HRMS (ESI)** calcd for [M+H] $C_{34}H_{25}BrNO_3$, m/z: 574.1013, found: 574.1016.

benzyl (1-(10-hydroxyphenanthren-9-yl)naphthalen-2-yl)carbamate (3v)

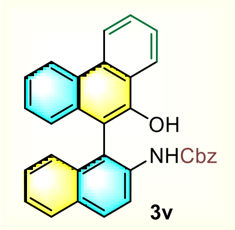

Yield: 91%. **^{1}H NMR (400 MHz, $CDCl_3$)** δ 8.85 (d, *J* = 8.0 Hz, 1H), 8.79 (d, *J* = 8.0 Hz, 1H), 8.63 (d, *J* = 8.8 Hz, 1H), 8.51 (dd, *J* = 8.0, 1.6 Hz, 1H), 8.13 (d, *J* = 9.2 Hz, 1H), 7.98 (d, *J* = 8.0 Hz, 1H), 7.87-7.83 (m, 1H), 7.79-7.75 (m, 1H), 7.59-7.55 (m, 1H), 7.49-7.45 (m, 1H), 7.41-7.37 (m, 1H), 7.33-7.28 (m, 5H), 7.27-7.22 (m, 2H), 7.10 (dd, *J* = 8.4, 1.6 Hz, 1H), 6.68 (s, 1H), 5.61 (s, 1H), 5.06 (d, *J* = 12.4 Hz, 1H), 5.02 (d, *J* = 12.0 Hz, 1H). **^{13}C NMR (100 MHz, $CDCl_3$)** δ 153.6, 148.4, 136.3, 135.7, 133.0, 132.0, 131.6, 130.9, 130.5, 128.5, 128.3, 128.3, 128.3, 128.0, 127.7, 127.5, 127.0, 126.9, 125.3, 125.2, 125.1, 124.8, 124.8, 123.6, 123.0, 122.8, 119.7, 116.7, 108.9, 67.1. **HRMS (ESI)** calcd for [M+H] $C_{32}H_{24}NO_3$, m/z: 470.1751, found: 470.1753.

benzyl (1-(10-hydroxyphenanthren-9-yl)-7-methoxynaphthalen-2-yl)carbamate (3w)

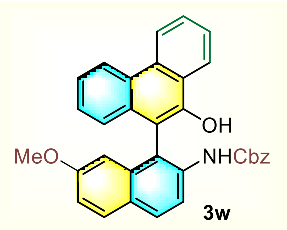

Yield: 90%. **^{1}H NMR (400 MHz, $CDCl_3$)** δ 8.79 (dd, *J* = 8.4, 1.2 Hz, 1H), 8.72 (dd, *J* = 8.4, 1.6 Hz, 1H), 8.44 (dd, *J* = 8.4, 1.6 Hz, 1H), 8.41 (d, *J* = 9.2 Hz, 1H), 7.99 (d, *J* = 9.2 Hz, 1H), 7.83 (d, *J* = 9.2 Hz, 1H), 7.82-7.78 (m, 1H), 7.73-7.69 (m, 1H), 7.54-7.50 (m, 1H), 7.36-7.32 (m, 1H), 7.27-7.23 (m, 3H), 7.20-7.17 (m, 2H), 7.10-7.06 (m, 2H), 6.54 (s, 1H), 6.48 (d, *J* = 2.4 Hz, 1H), 5.47 (s, 1H), 5.03 (d, *J* = 12.4 Hz, 1H), 5.00 (d, *J* = 12.4 Hz, 1H), 3.43 (s, 3H). **^{13}C NMR (100 MHz, $CDCl_3$)** δ 159.0, 153.6, 148.2, 136.8, 135.7, 134.4, 131.9, 131.3, 130.3, 129.9, 128.5, 128.3, 128.3, 128.0, 127.7, 127.0, 126.8, 126.3, 125.0, 124.8, 124.7, 123.6, 123.0, 122.7, 117.5, 117.2, 115.3, 109.0, 103.8, 67.1, 55.1. **HRMS (ESI)** calcd for [M+H] $C_{33}H_{26}NO_4$, m/z: 500.1857, found: 500.1859.

methyl 6-(((benzyloxy)carbonyl)amino)-5-(10-hydroxyphenanthren-9-yl)-2-naphthoate (3x)

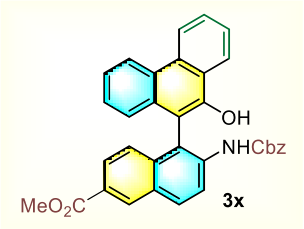

Yield: 92%. **^{1}H NMR (400 MHz, CDCl$_3$)** δ 8.79 (d, *J* = 7.2 Hz, 1H), 8.73 (dd, *J* = 8.4, 1.2 Hz, 1H), 8.68 (d, *J* = 8.8 Hz, 1H), 8.57 (d, *J* = 2.0 Hz, 1H), 8.46 (dd, *J* = 8.0, 1.6 Hz, 1H), 8.13 (d, *J* = 9.6 Hz, 1H), 7.84-7.79 (m, 1H), 7.77 (dd, *J* = 8.8, 1.6 Hz, 1H), 7.74-7.70 (m, 1H), 7.55-7.50 (m, 1H), 7.35-7.30 (m, 1H), 7.28-7.24 (m, 3H), 7.23-7.19 (m, 3H), 6.96 (dd, *J* = 8.4, 1.6 Hz, 1H), 6.70 (s, 1H), 5.65 (s, 1H), 5.04 (d, *J* = 12.4 Hz, 1H), 5.01 (d, *J* = 12.4 Hz, 1H), 3.90 (s, 3H). **^{13}C NMR (100 MHz, CDCl$_3$)** δ 167.0, 153.3, 148.5, 138.5, 135.4, 135.4, 132.0, 131.9, 131.3, 131.3, 129.7, 128.5, 128.4, 128.4, 128.2, 127.8, 127.1, 126.9, 126.8, 126.6, 125.4, 125.0, 124.9, 124.6, 123.6, 123.1, 122.7, 119.9, 116.3, 108.1, 67.3, 52.3. **HRMS (ESI)** calcd for [M+H] $C_{34}H_{26}NO_5$, m/z: 528.1806, found: 528.1807.

benzyl (1-(2-hydroxyphenyl)naphthalen-2-yl)carbamate (3y)

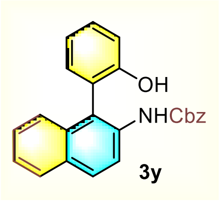

Yield: 75%. **^{1}H NMR (400 MHz, CDCl$_3$)** δ 8.36 (d, *J* = 8.4 Hz, 1H), 7.90-7.86 (m, 2H), 7.59-7.53 (m, 1H), 7.42-7.38 (m, 2H), 7.33-7.24 (m, 9H), 6.42 (s, 1H), 5.36 (s, 1H), 5.08 (s, 2H). **^{13}C NMR (100 MHz, CDCl$_3$)** δ 153.5, 151.3, 137.4, 135.8, 133.0, 132.0, 130.8, 130.1, 129.2, 128.5, 128.3, 128.3, 128.3, 127.3, 124.4, 124.1, 123.8, 122.7, 120.5, 117.8, 115.3, 67.0. **HRMS (ESI)** calcd for [M+H] $C_{24}H_{20}NO_3$, m/z: 370.1438, found: 370.1435.

benzyl (1-(5-bromo-2-hydroxyphenyl)naphthalen-2-yl)carbamate (3z)

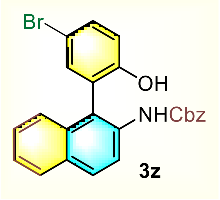

Yield: 72%. **^{1}H NMR (400 MHz, CDCl$_3$)** δ 8.20 (d, *J* = 8.8 Hz, 1H), 7.87 (d, *J* = 9.2 Hz, 1H), 7.80 (d, *J* = 7.6 Hz, 1H), 7.45-7.29 (m, 8H), 7.28-7.22 (m, 2H), 6.92 (d, *J* = 8.4 Hz, 1H), 6.58 (s, 1H), 5.47 (s, 1H), 5.05 (d, *J* = 12.4 Hz, 1H), 5.00 (d, *J* = 12.0 Hz, 1H). **^{13}C NMR (100 MHz, CDCl$_3$)** δ 153.8, 153.2, 135.7, 134.4, 134.0, 133.5, 132.5, 130.8, 130.2, 128.7, 128.5, 128.4, 128.2, 127.4, 125.4, 124.9, 123.2, 120.3, 119.7, 118.5, 113.2, 67.4. **HRMS (ESI)** calcd for [M+H] $C_{24}H_{19}BrNO_3$, m/z: 448.0543, found: 448.0540.

benzyl (1-(3-fluoro-6-hydroxy-2,4-dimethylphenyl)naphthalen-2-yl)carbamate (3aa)

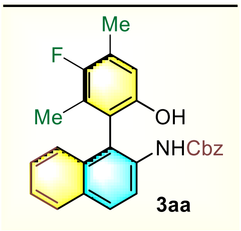

Yield: 72%. **^{1}H NMR (400 MHz, CDCl$_3$)** δ 7.96 (d, *J* = 7.6 Hz, 1H), 7.91-7.87 (m, 2H), 7.42-7.14 (m, 9H), 6.16 (s, 1H), 5.45 (s, 1H), 5.05 (d, *J* = 12.0 Hz, 1H), 5.01 (d, *J* = 12.0 Hz, 1H), 2.41 (d, *J* = 2.4 Hz, 3H), 1.86 (d, *J* = 2.8 Hz, 3H). **^{13}C NMR (100 MHz, CDCl$_3$)** δ 156.8 (d, *J* = 240.3 Hz), 153.8, 151.4, 135.8, 132.6, 131.0, 129.3, 128.5, 128.5, 128.3, 128.2, 127.5, 126.1 (d, *J* = 19.0 Hz), 125.8 (d, *J* = 17.9 Hz), 123.9, 123.5, 122.1, 121.0, 117.9, 113.5, 113.4, 67.0, 15.1 (d, *J* = 3.7 Hz) , 11.9 (d, *J* = 4.4 Hz). **^{19}F NMR (375 MHz,**

$CDCl_3$) δ -123.4. **HRMS (ESI)** calcd for [M+H] $C_{26}H_{23}FNO_3$, m/z: 416.1657, found: 416.1658.

benzyl (2-(2-hydroxynaphthalen-1-yl)-3,5-dimethylphenyl)carbamate (3ab)

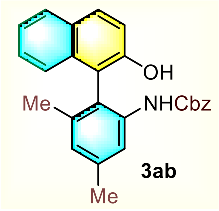

Yield: 62%. **^{1}H NMR (400 MHz, $CDCl_3$)** δ 8.03 (s, 1H), 7.90 (d, *J* = 8.8 Hz, 1H), 7.88-7.85 (m, 1H), 7.41-7.30 (m, 6H), 7.27-7.15 (m, 3H), 7.02 (d, *J* = 1.6 Hz, 1H), 7.02 (s, 1H), 6.25 (s, 1H), 5.04 (s, 2H), 2.47 (s, 3H), 1.90 (s, 3H). **^{13}C NMR (100 MHz, $CDCl_3$)** δ 153.5, 151.4, 139.9, 139.3, 137.3, 135.9, 132.7, 130.8, 129.4, 128.5, 128.5, 128.3, 128.2, 127.3, 126.7, 123.8, 123.7, 118.8, 118.3, 117.7, 114.1, 66.9, 21.7, 19.8. **HRMS (ESI)** calcd for [M+H] $C_{26}H_{24}NO_3$, m/z: 398.1751, found: 398.1750.

benzyl (2-(2-hydroxynaphthalen-1-yl)phenyl)carbamate (3ac)

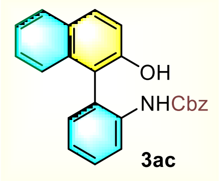

Yield: 68%. **^{1}H NMR (400 MHz, $CDCl_3$)** δ 8.28 (d, *J* = 9.2 Hz, 1H), 7.84 (d, *J* = 9.2 Hz, 1H), 7.76 (d, *J* = 8.0 Hz, 1H), 7.35-7.21 (m, 9H), 7.08-6.99 (m, 3H), 6.99 (s, 1H), 5.05 (s, 1H), 5.09 (d, *J* = 12.0 Hz, 1H), 5.04 (d, *J* = 12.0 Hz, 1H). **^{13}C NMR (100 MHz, $CDCl_3$)** δ 153.9, 153.7, 135.9, 134.6, 132.7, 131.7, 130.7, 130.6, 129.8, 128.6, 128.4, 128.4, 128.2, 127.1, 125.1, 125.1, 121.5, 120.7, 120.0, 119.8, 116.6, 67.2. **HRMS (ESI)** calcd for [M+H] $C_{24}H_{20}NO_3$, m/z: 370.1438, found: 370.1438.

benzyl (1-(2-hydroxy-2'-iodo-6,6'-dimethyl-[1,1'-biphenyl]-3-yl)naphthalen-2-yl)carbamate (3ad) 72% yield, dr = 1.2/1

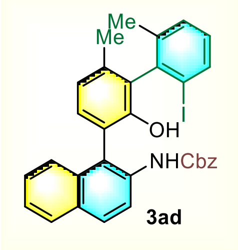

^{1}H NMR (400 MHz, $CDCl_3$) δ 8.48 (d, *J* = 9.2 Hz, 1H), 7.96 (d, *J* = 9.2 Hz, 1H), 7.90-7.88 (m, 1H), 7.80 (d, *J* = 8.0 Hz, 1H), 7.50-7.30 (m, 9H), 7.19-7.10 (m, 2H), 7.02 (t, *J* = 8.0 Hz, 1H), 6.94 (s, 1H), 5.16-5.09 (m, 2H), 4.62 (s, 1H), 2.20 (s, 3H), 2.06 (s, 3H). **^{13}C NMR (100 MHz, $CDCl_3$)** δ 153.5, 149.9, 140.2, 138.8, 138.2, 136.9, 135.8, 134.6, 133.1, 131.6, 131.6, 130.5, 130.1, 129.8, 129.4, 128.6, 128.5, 128.3, 128.1, 126.7, 125.1, 124.7, 123.2, 120.2, 119.3, 118.6, 102.2, 67.1, 21.4, 19.6. **HRMS (ESI)** calcd for [M+H] $C_{32}H_{27}INO_3$, m/z: 600.1030, found: 600.1031.

^{1}H NMR (400 MHz, $CDCl_3$) δ 8.37 (d, *J* = 8.4 Hz, 1H), 7.95 (d, *J* = 9.2 Hz, 1H), 7.89-7.87 (m, 1H), 7.84 (d, *J* = 8.0 Hz, 1H), 7.66-7.62 (m, 1H), 7.48-7.31 (m, 8H), 7.19 (d, *J* = 7.6 Hz, 1H), 7.11 (d, *J* = 7.6 Hz, 1H), 7.02 (t, *J* = 8.0 Hz, 1H), 6.79 (s, 1H), 5.21-5.14 (m, 2H), 4.65 (s, 1H), 2.08 (s, 6H). **^{13}C NMR (100 MHz, $CDCl_3$)** δ 153.5, 149.9, 140.3, 138.8, 138.3, 137.0, 136.0, 134.1, 132.8, 131.6, 131.4, 130.7, 130.2, 129.8, 129.5, 128.6, 128.3, 128.2, 126.9, 125.7, 124.9, 123.2, 121.0, 119.6, 118.7, 115.6, 101.9, 67.1, 21.3, 19.6. **HRMS (ESI)** calcd for [M+H] $C_{32}H_{27}INO_3$, m/z: 600.1030, found: 600.1030.

General procedures for synthesis of BINAM derivatives 5

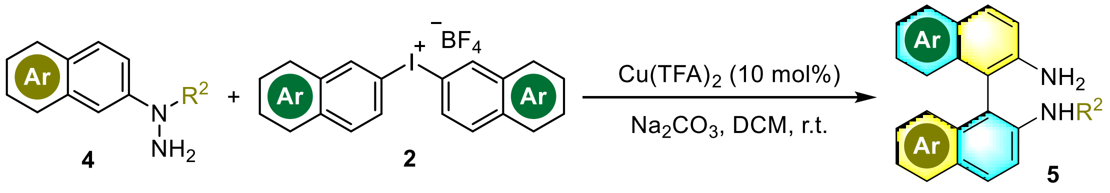

4 (0.20 mmol), **2** (0.24 mmol), Na_2CO_3 (27.6 mg, 0.26 mmol) and $Cu(TFA)_2$ (5.8 mg, 10 mol%) were added to a bottle with a magnetic stirring bar. DCM (4.0 mL) was added and the reaction mixture was stirred at room temperature till **4** was completely consumed (monitored by TLC). After evaporated the solvent, the residue was purified by flash chromatography eluted with DCM to afford the corresponding product **5**.

N^2-methyl-[1,1'-binaphthalene]-2,2'-diamine (5a)

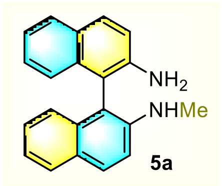

Yield: 52%. **^{1}H NMR (400 MHz, $CDCl_3$)** δ 7.92 (d, *J* = 8.8 Hz, 1H), 7.83-7.79 (m, 3H), 7.27-7.24 (m, 2H), 7.22-7.17 (m, 3H), 7.15 (d, *J* = 8.4 Hz, 1H), 7.04 (d, *J* = 8.4 Hz, 1H), 7.02 (dd, *J* = 9.2, 2.8 Hz, 1H), 3.85 (brs, 1H), 2.85 (s, 3H), 2.55 (brs, 2H). **^{13}C NMR (100 MHz, $CDCl_3$)** δ 144.9, 142.5, 133.5, 133.0, 129.3, 129.1, 128.1, 127.8, 127.7, 127.2, 126.4, 126.3, 123.6, 123.2, 122.0, 121.4, 117.9, 113.0, 112.1, 111.5, 30.7. **HRMS (ESI)** calcd for [M+H] $C_{21}H_{21}N_2$, m/z: 299.1543, found: 299.1544.

N^2-phenyl-[1,1'-binaphthalene]-2,2'-diamine (5b)

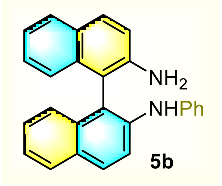

Yield: 50%. **^{1}H NMR (400 MHz, $CDCl_3$)** δ 7.93-7.84 (m, 4H), 7.76 (d, *J* = 9.2 Hz, 1H), 7.48-7.06 (m, 11H), 6.98 (t, *J* = 7.6 Hz, 1H), 5.67 (brs, 1H), 3.22 (brs, 2H). **^{13}C NMR (100 MHz, $CDCl_3$)** δ 142.9, 142.8, 140.2, 134.0, 133.8, 129.8, 129.5, 129.3, 129.2, 128.5, 128.3, 128.2, 127.1, 126.9, 124.6, 123.9, 123.4, 122.6, 122.0, 119.8, 118.4, 118.0, 116.9, 112.1. **HRMS (ESI)** calcd for [M+H] $C_{26}H_{21}N_2$, m/z: 361.1699, found: 361.1699.

N^2-benzyl-[1,1'-binaphthalene]-2,2'-diamine (5c)

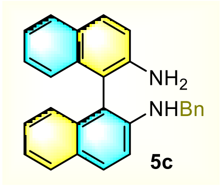

Yield: 50%. **^{1}H NMR (400 MHz, $CDCl_3$)** δ 7.86-7.78 (m, 4H), 7.31-7.18 (m, 11H), 7.15-7.13 (m, 1H), 7.08-7.05 (m, 1H), 4.44 (s, 2H), 3.92 (brs, 3H). **^{13}C NMR (100 MHz, $CDCl_3$)** δ 143.9, 143.0, 139.8, 134.0, 133.6, 129.7, 129.6, 128.6, 128.5, 128.2, 128.2, 127.8, 126.9, 126.9, 126.9, 126.8, 124.2, 123.8, 122.5, 122.0, 118.4, 114.4, 112.5, 112.3, 47.7. **HRMS (ESI)** calcd for [M+H] $C_{27}H_{23}N_2$, m/z: 375.1856, found: 375.1855.

N^2-benzyl-6'-fluoro-[1,1'-binaphthalene]-2,2'-diamine (5d)

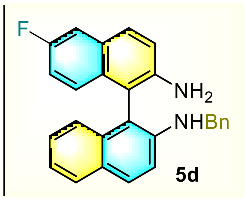

Yield: 56%. **^{1}H NMR (400 MHz, $CDCl_3$)** δ 7.84 (d, *J* = 9.2 Hz, 1H), 7.82-7.79 (m, 1H), 7.78 (d, *J* = 8.8 Hz, 1H), 7.46 (dd, *J* = 10.0, 2.8 Hz, 1H), 7.28-7.20 (m, 9H), 7.12-7.09 (m, 1H), 7.05-7.00 (m, 2H), 4.44 (s, 2H), 4.19 (brs, 1H), 3.72 (brs, 2H). **^{13}C NMR (100 MHz, $CDCl_3$)** δ 158.9 (d, *J* = 241.0 Hz), 143.9, 142.4 (d, *J* = 2.0 Hz), 139.8, 133.5, 130.9, 129.7, 128.9 (d, *J* = 8.0 Hz), 128.8 (d, *J* = 5.0 Hz), 128.5, 128.2, 127.7, 127.0, 126.9, 126.8, 126.5 (d, *J* = 8.0 Hz), 123.6, 122.1, 119.6, 116.7 (d, *J* = 24.0 Hz), 114.3, 112.7, 112.1, 111.3 (d, *J* = 20.0 Hz), 47.7. **^{19}F NMR (376 MHz, $CDCl_3$)** δ -120.50. **HRMS (ESI)** calcd for [M+H] $C_{27}H_{22}FN_2$, m/z: 393.1762, found: 393.1762.

IV. Control experiments

Table S3. The role of copper catalyst and base in the conditions[a]

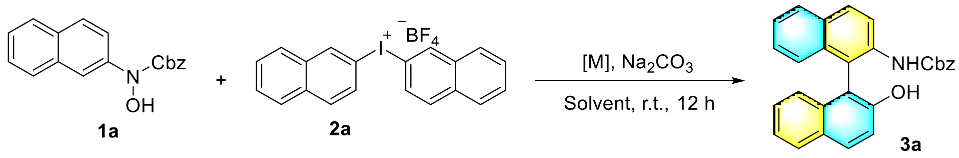

Entry	[M](10 mol%)	Base	Solvent	Yield (%)[b]
1	$Al(OTf)_3$	Na_2CO_3	DCM	71
2	$Mg(OTf)_2$	Na_2CO_3	DCM	72
3	$Zn(OTf)_2$	Na_2CO_3	DCM	70
4	$Ni(OTf)_2$	Na_2CO_3	DCM	68
5	—	Na_2CO_3	DCM	70
6	$Cu(TFA)_2$	—	DCM	33
7	$Cu(TFA)_2$	Na_2CO_3	DCM	98

[a]All reactions were performed with [M] (10 mol%), **1a** (0.10 mmol), **2a** (0.12 mmol) and Na_2CO_3 (0.13 mmol) in DCM (2.0 mL) at room temperature for 12 h; [b]Yield was determined by ^{1}H-NMR analysis of the crude reaction mixture using 1,3,5-trimethoxybenzene as the internal standard.

V. Copies of NMR spectra

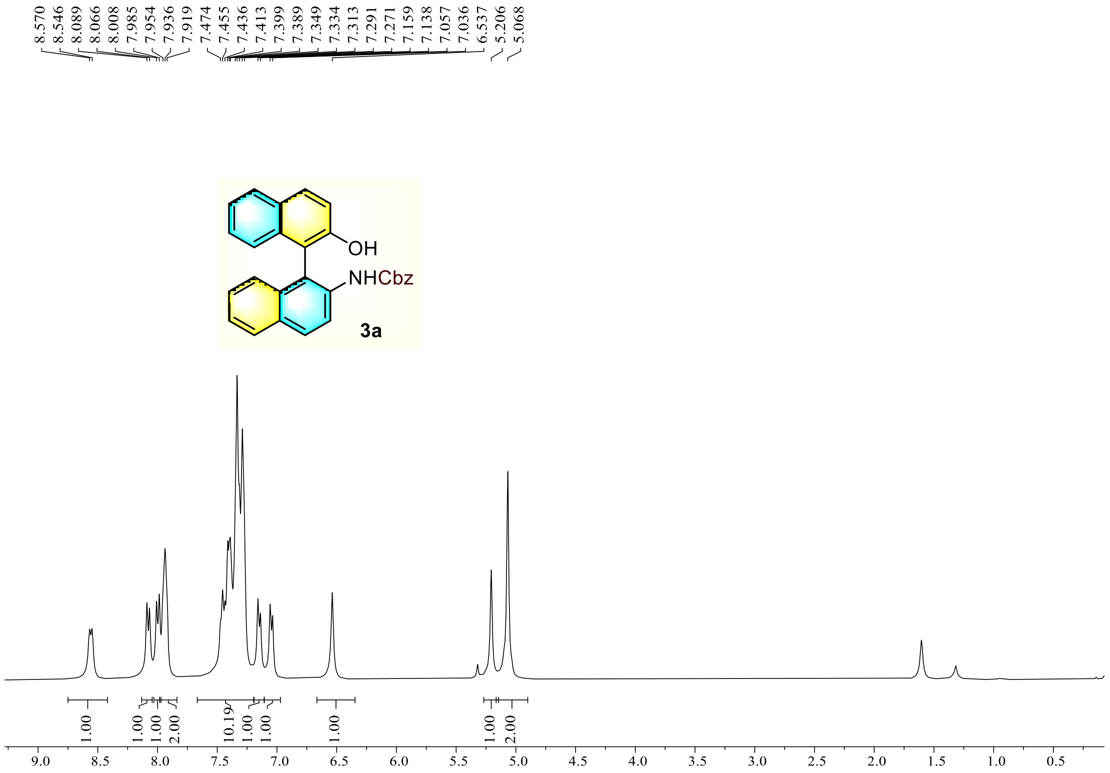

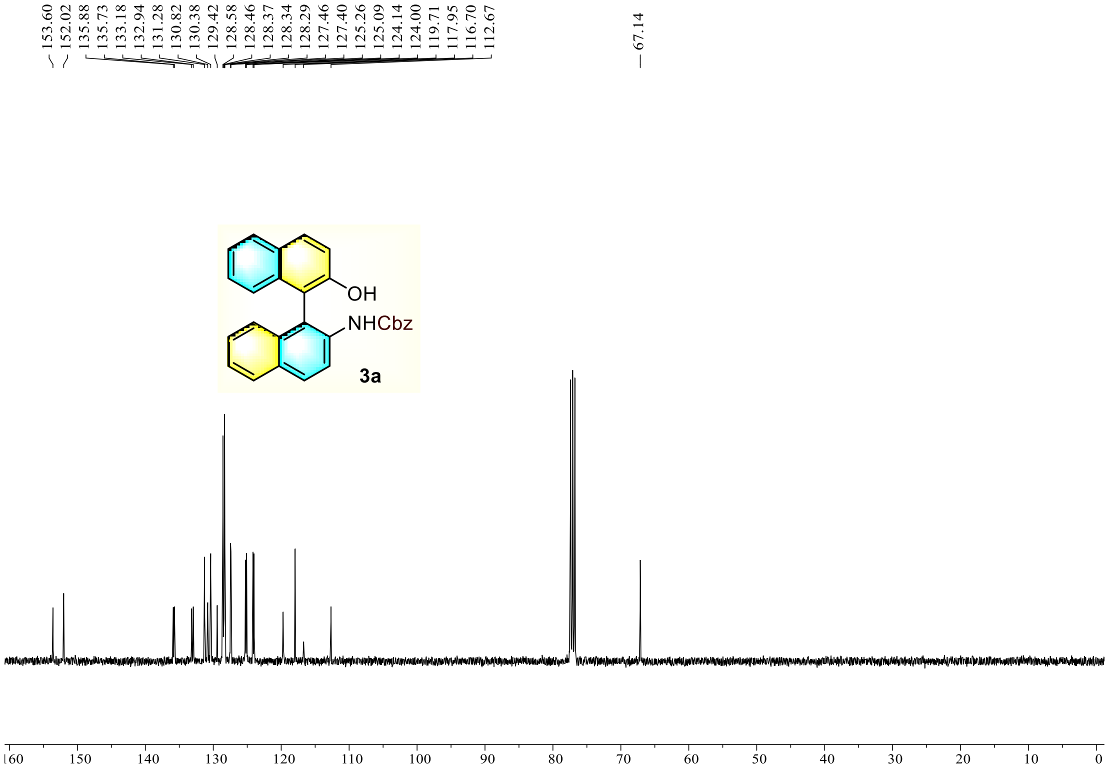

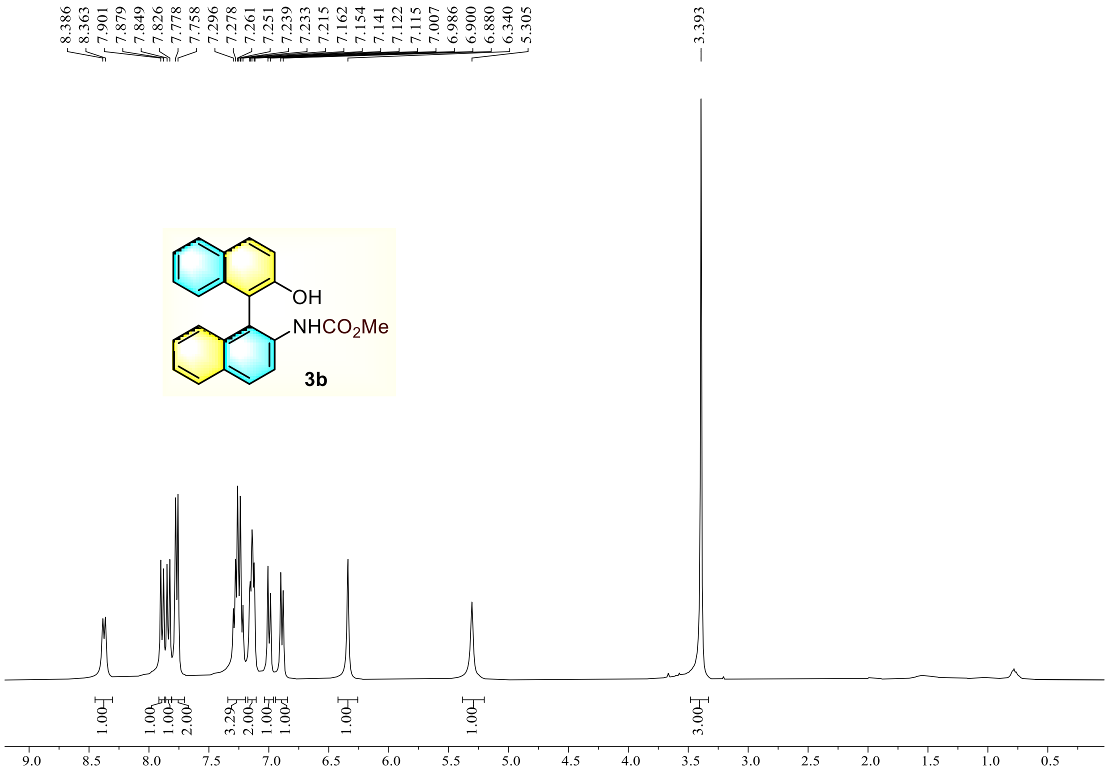
8.386
8.363
7.901
7.879
7.849
7.826
7.778
7.758
7.296
7.278
7.261
7.251
7.239
7.233
7.215
7.162
7.154
7.141
7.122
7.115
7.007
6.986
6.900
6.880
6.340
5.305
3.393
OH
NHCO2Me
3b
1.00
1.00
1.00
2.00
3.29
2.00
1.00
1.00
1.00
1.00
3.00
9.0 8.5 8.0 7.5 7.0 6.5 6.0 5.5 5.0 4.5 4.0 3.5 3.0 2.5 2.0 1.5 1.0 0.5

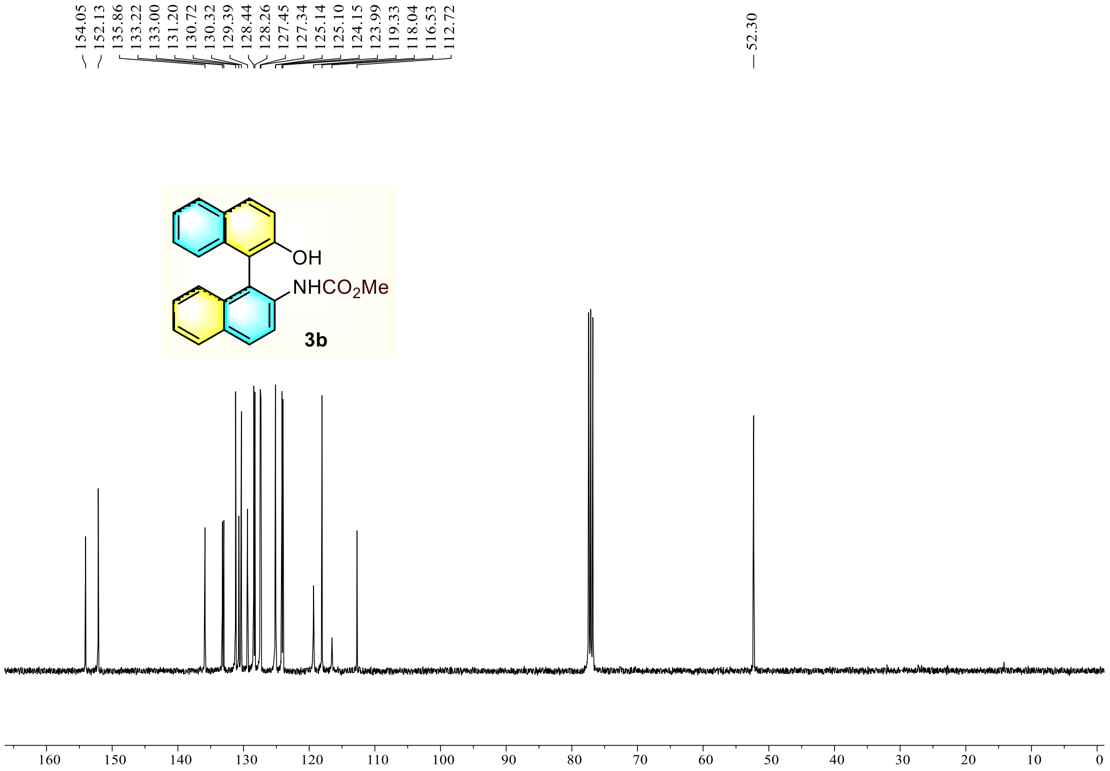
154.05
152.13
135.86
133.22
133.00
131.20
130.72
130.32
129.39
128.44
128.26
127.45
127.34
125.14
125.10
124.15
123.99
119.33
118.04
116.53
112.72
52.30
OH
NHCO2Me
3b
160 150 140 130 120 110 100 90 80 70 60 50 40 30 20 10 0

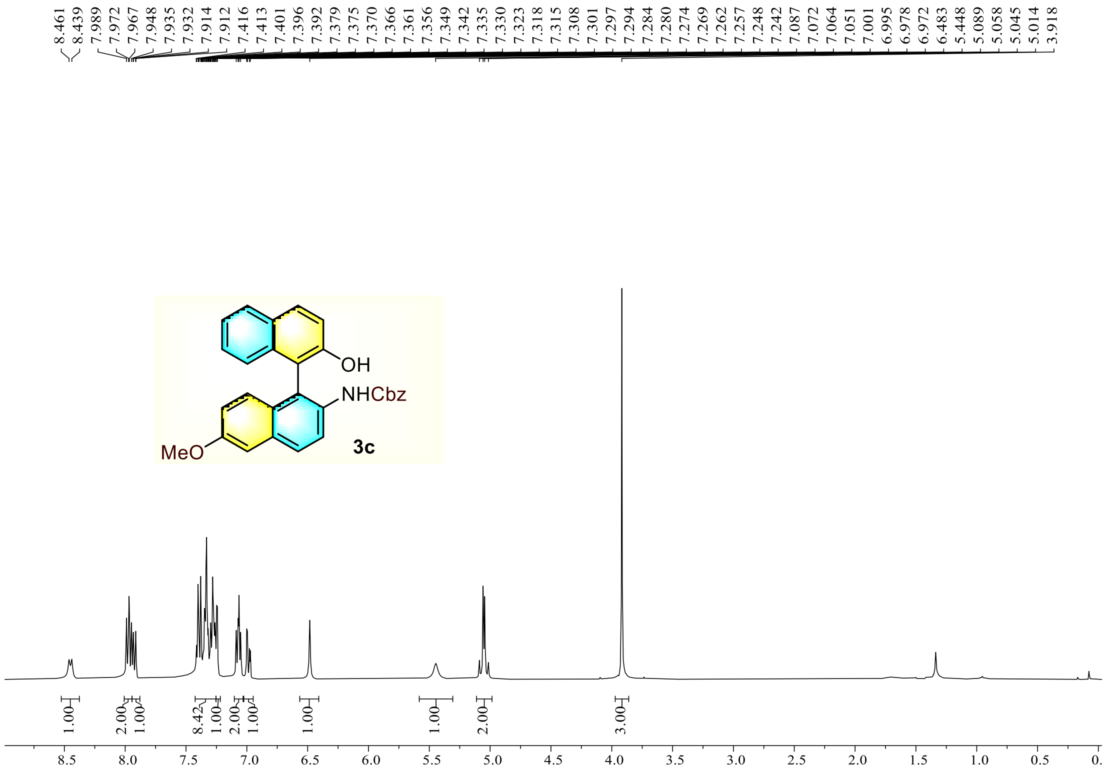
OH
NHCbz
MeO
3c

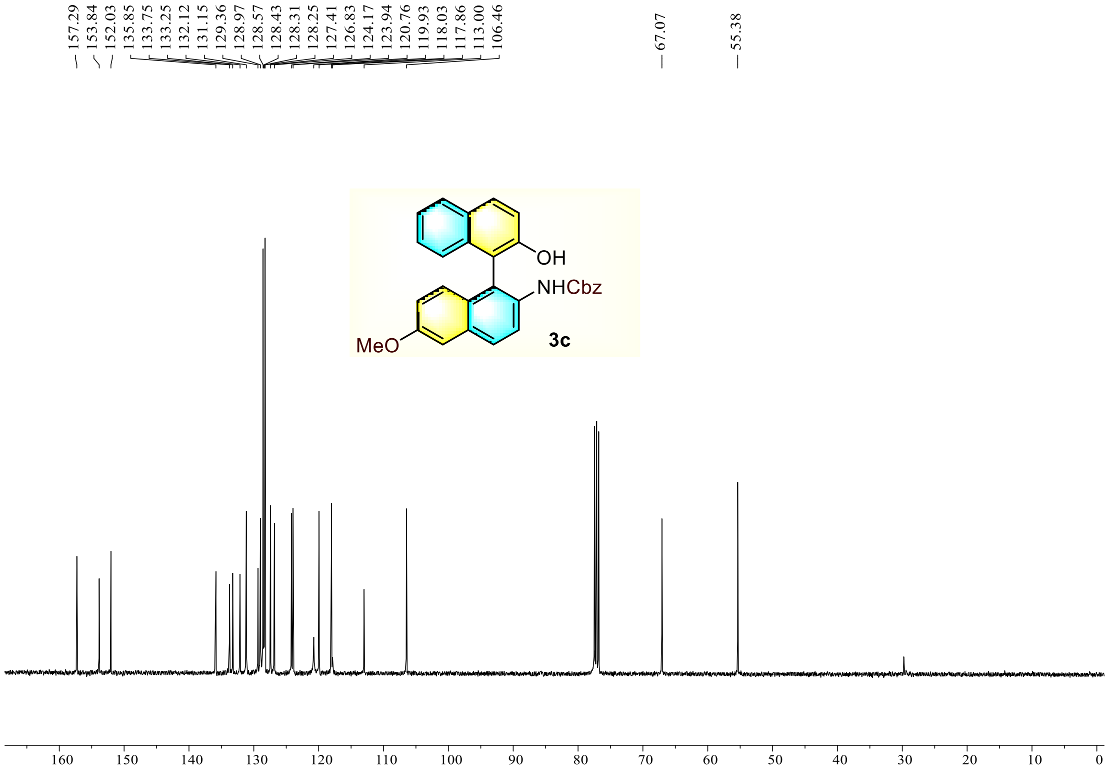
OH
NHCbz
MeO
3c

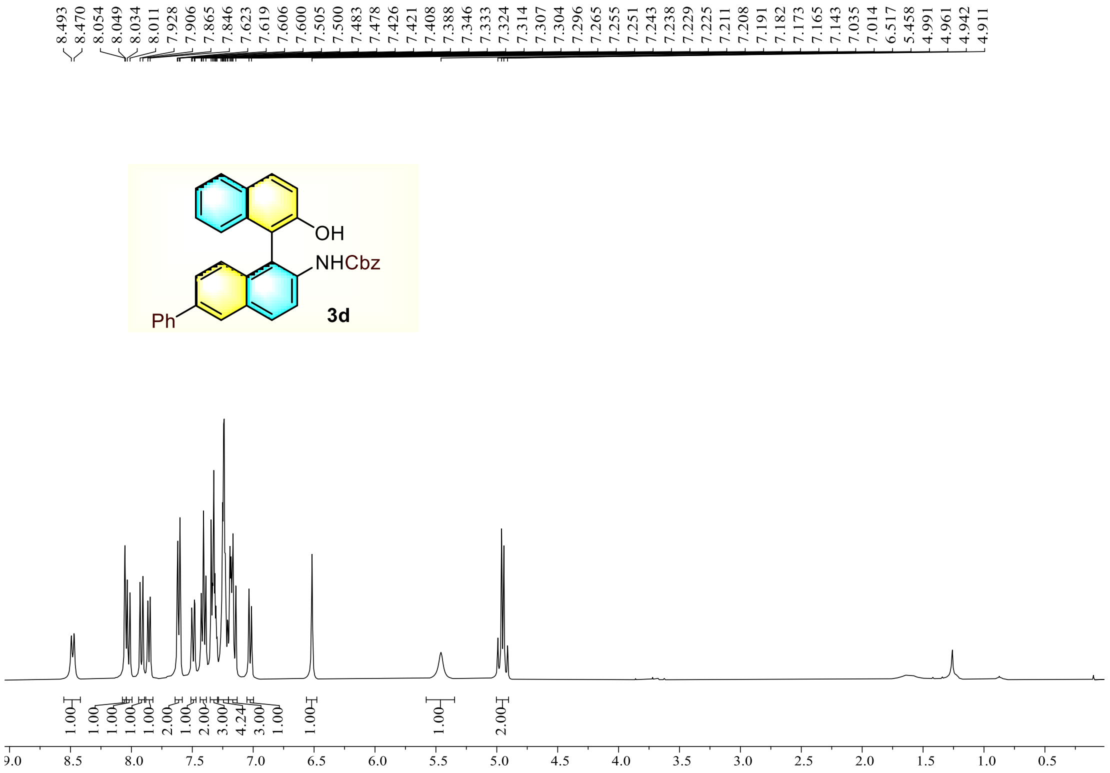
8.493
8.470
8.054
8.049
8.034
8.011
7.928
7.906
7.865
7.846
7.623
7.619
7.606
7.600
7.505
7.500
7.483
7.478
7.426
7.421
7.408
7.388
7.346
7.333
7.324
7.314
7.307
7.304
7.296
7.265
7.255
7.251
7.243
7.238
7.229
7.225
7.211
7.208
7.191
7.182
7.173
7.165
7.143
7.035
7.014
6.517
5.458
4.991
4.961
4.942
4.911
OH
NHCbz
Ph
3d
1.00
1.00
1.00
1.00
1.00
2.00
1.00
2.00
3.00
4.24
3.00
1.00
1.00
1.00
2.00

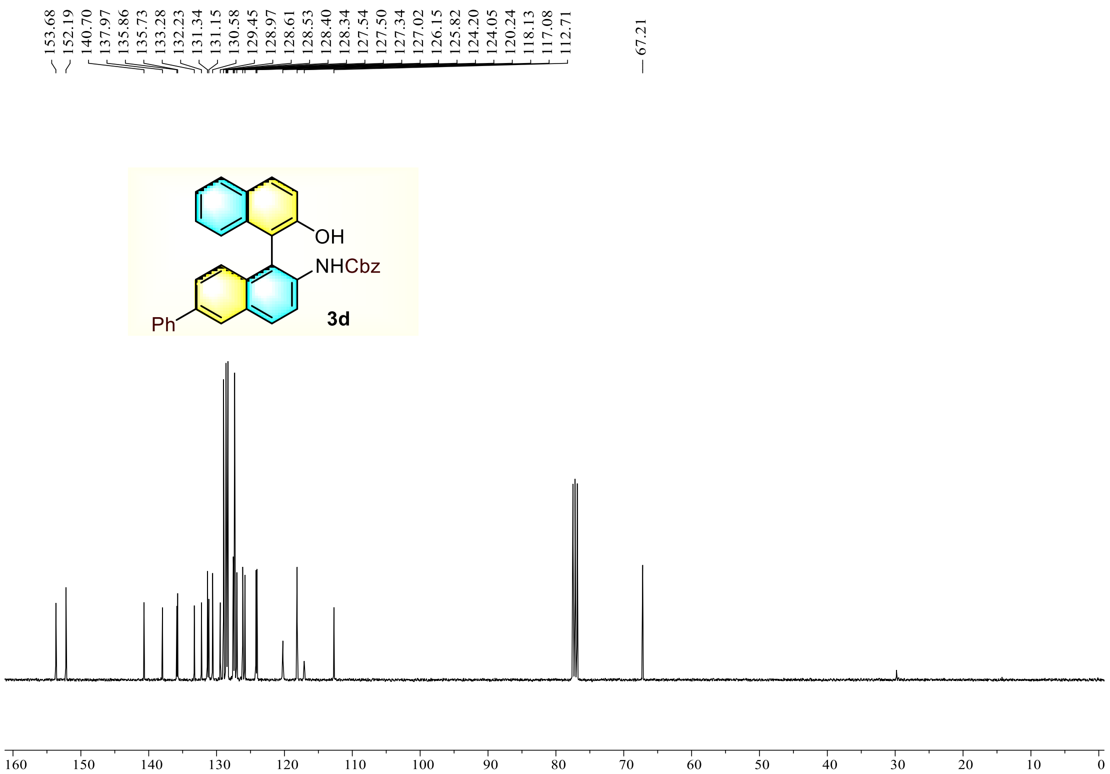
153.68
152.19
140.70
137.97
135.86
135.73
133.28
132.23
131.34
131.15
130.58
129.45
128.97
128.61
128.53
128.40
128.34
127.54
127.50
127.34
127.02
126.15
125.82
124.20
124.05
120.24
118.13
117.08
112.71
67.21
OH
NHCbz
Ph
3d

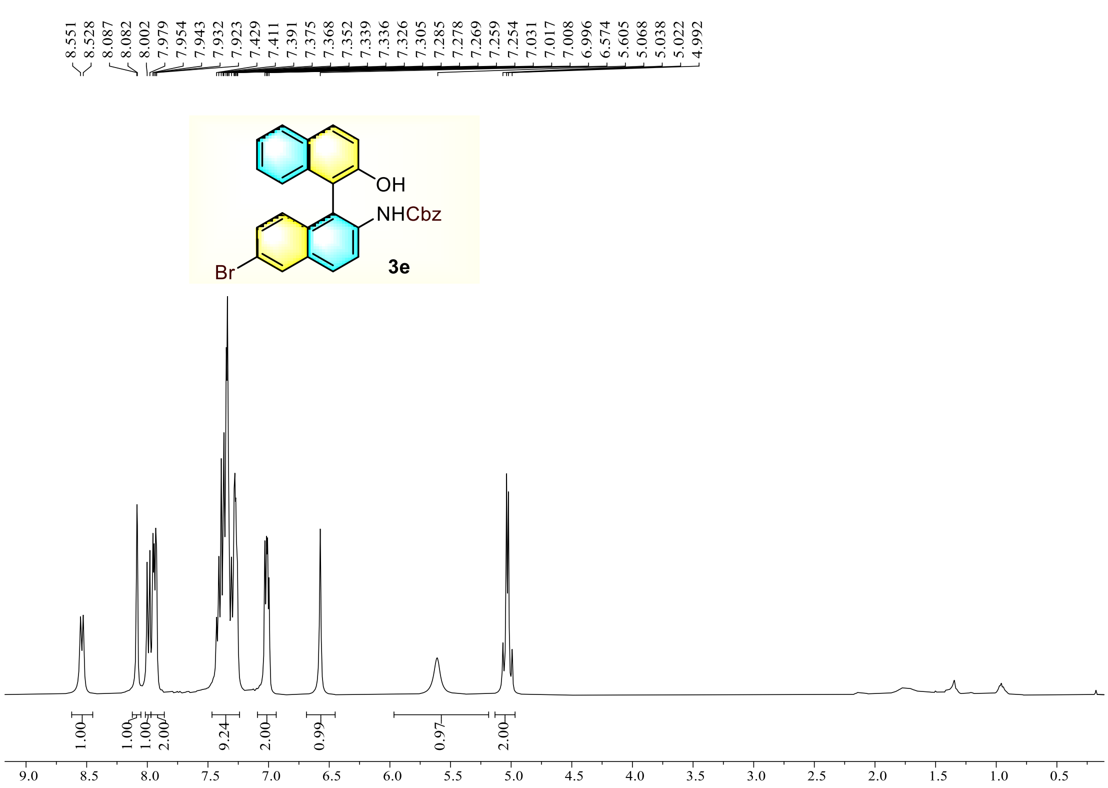

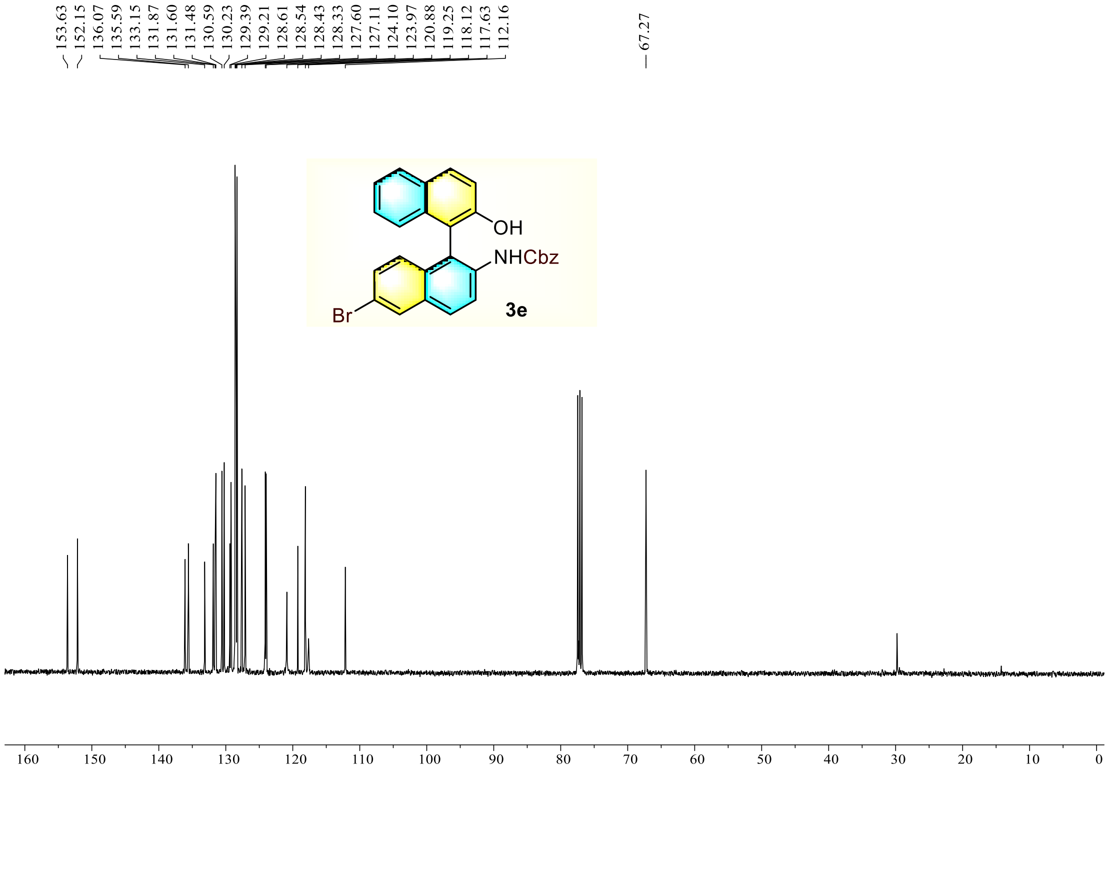

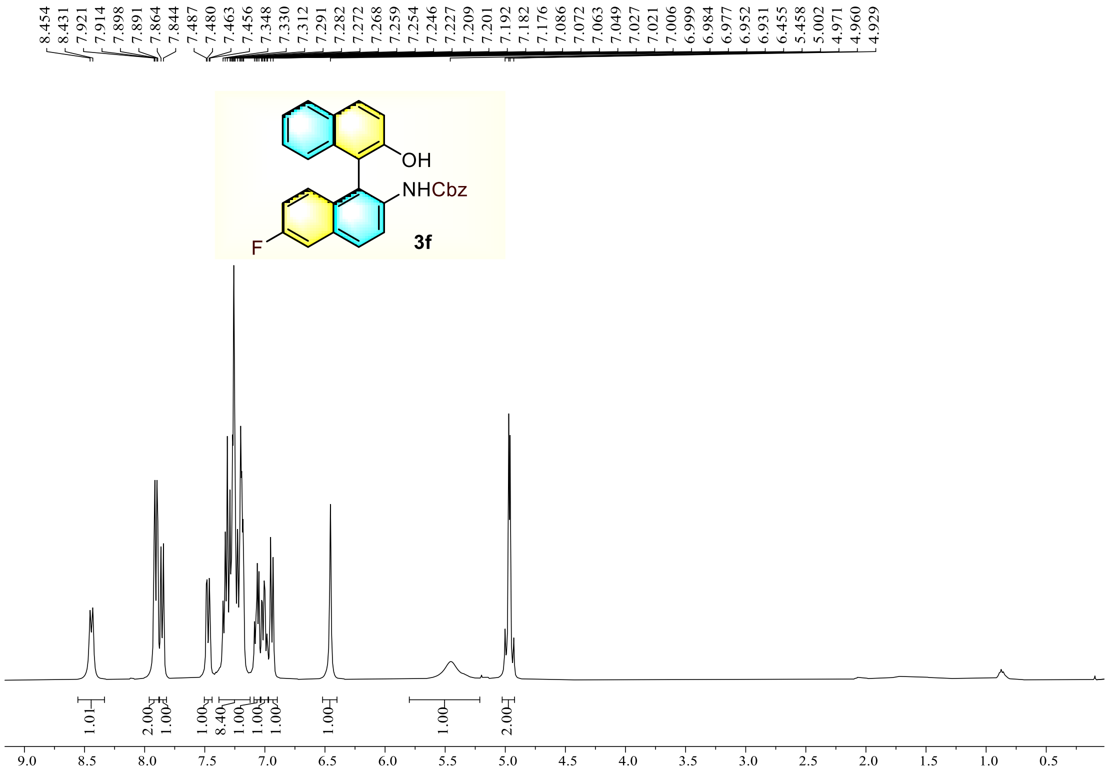
8.454
8.431
7.921
7.914
7.898
7.891
7.864
7.844
7.487
7.480
7.463
7.456
7.348
7.330
7.312
7.291
7.282
7.272
7.268
7.259
7.254
7.246
7.227
7.209
7.201
7.192
7.182
7.176
7.086
7.072
7.063
7.049
7.027
7.021
7.006
6.999
6.984
6.977
6.952
6.931
6.455
5.458
5.002
4.971
4.960
4.929
OH
NHCbz
F
3f
1.01
2.00
1.00
1.00
8.40
1.00
1.00
1.00
1.00
1.00
2.00

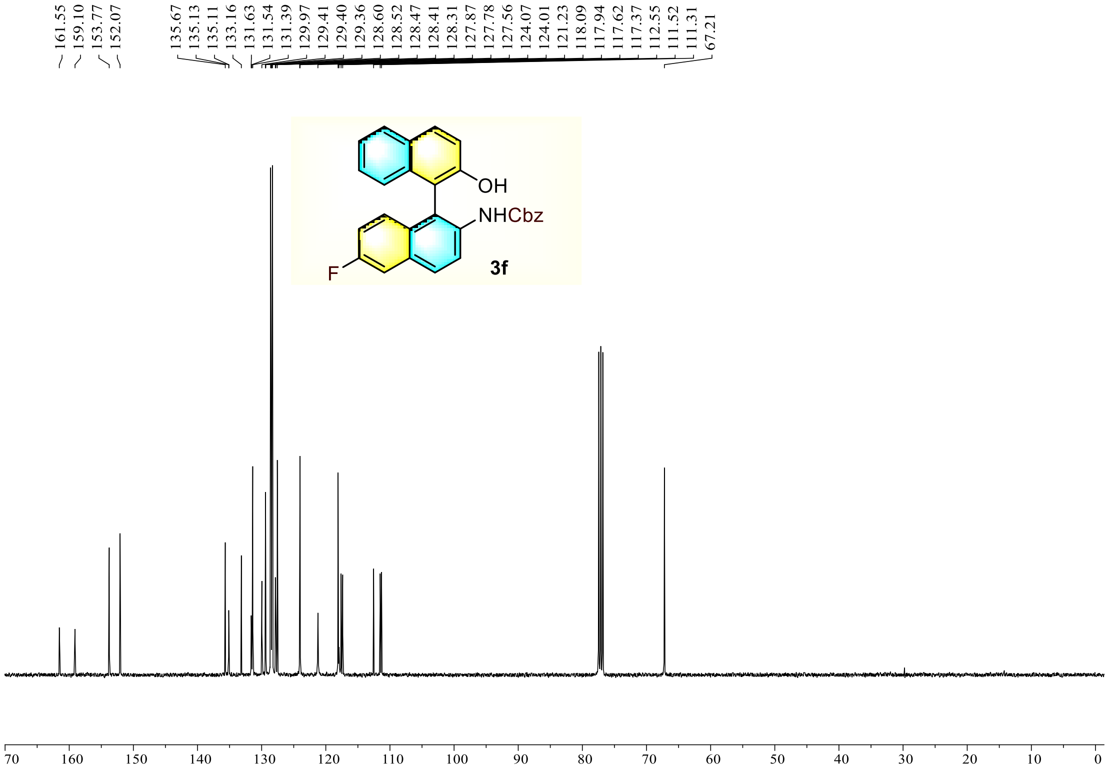
161.55
159.10
153.77
152.07
135.67
135.13
135.11
133.16
131.63
131.54
131.39
129.97
129.41
129.40
129.36
128.60
128.52
128.47
128.41
128.31
127.87
127.78
127.56
124.07
124.01
121.23
118.09
117.94
117.62
117.37
112.55
111.52
111.31
67.21
OH
NHCbz
F
3f

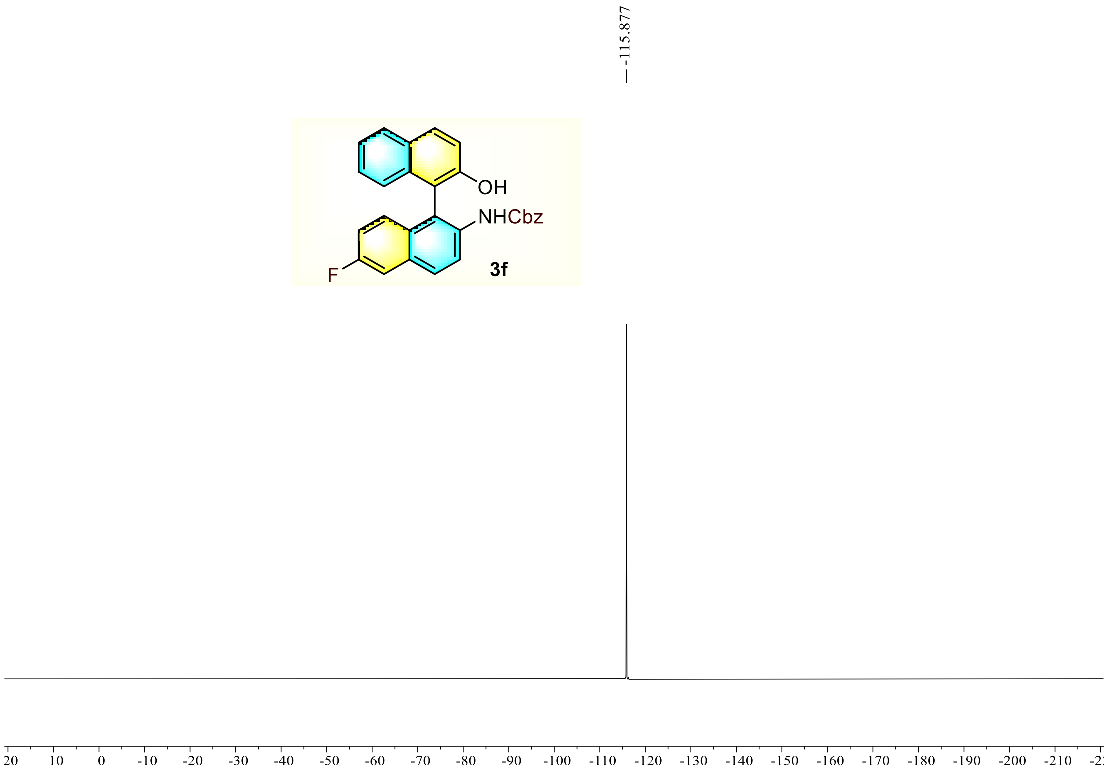
-115.877
OH
NHCbz
F
3f
20 10 0 -10 -20 -30 -40 -50 -60 -70 -80 -90 -100 -110 -120 -130 -140 -150 -160 -170 -180 -190 -200 -210

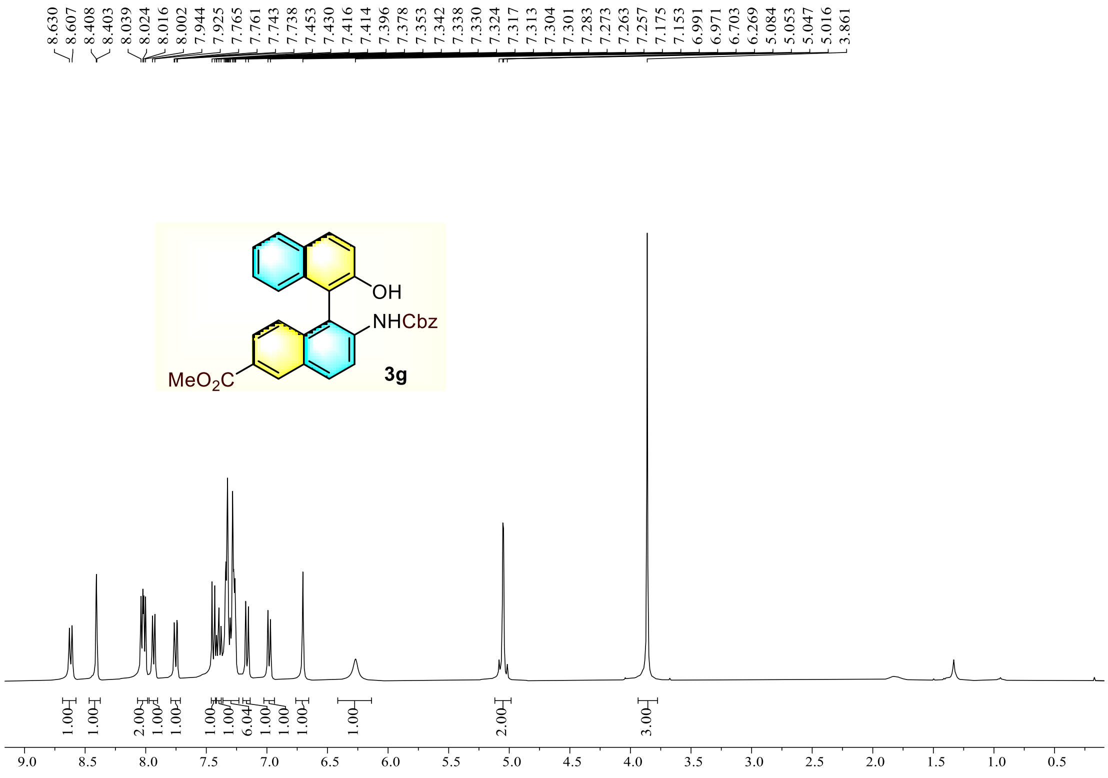
8.630 8.607 8.408 8.403 8.039 8.024 8.016 8.002 7.944 7.925 7.765 7.761 7.743 7.738 7.453 7.430 7.416 7.414 7.396 7.378 7.353 7.342 7.338 7.330 7.324 7.317 7.313 7.304 7.301 7.283 7.273 7.263 7.257 7.175 7.153 6.991 6.971 6.703 6.269 5.084 5.053 5.047 5.016 3.861
OH
NHCbz
MeO2C
3g
1.00 1.00 2.00 1.00 1.00 1.00 1.00 6.04 1.00 1.00 1.00 1.00 2.00 3.00
9.0 8.5 8.0 7.5 7.0 6.5 6.0 5.5 5.0 4.5 4.0 3.5 3.0 2.5 2.0 1.5 1.0 0.5

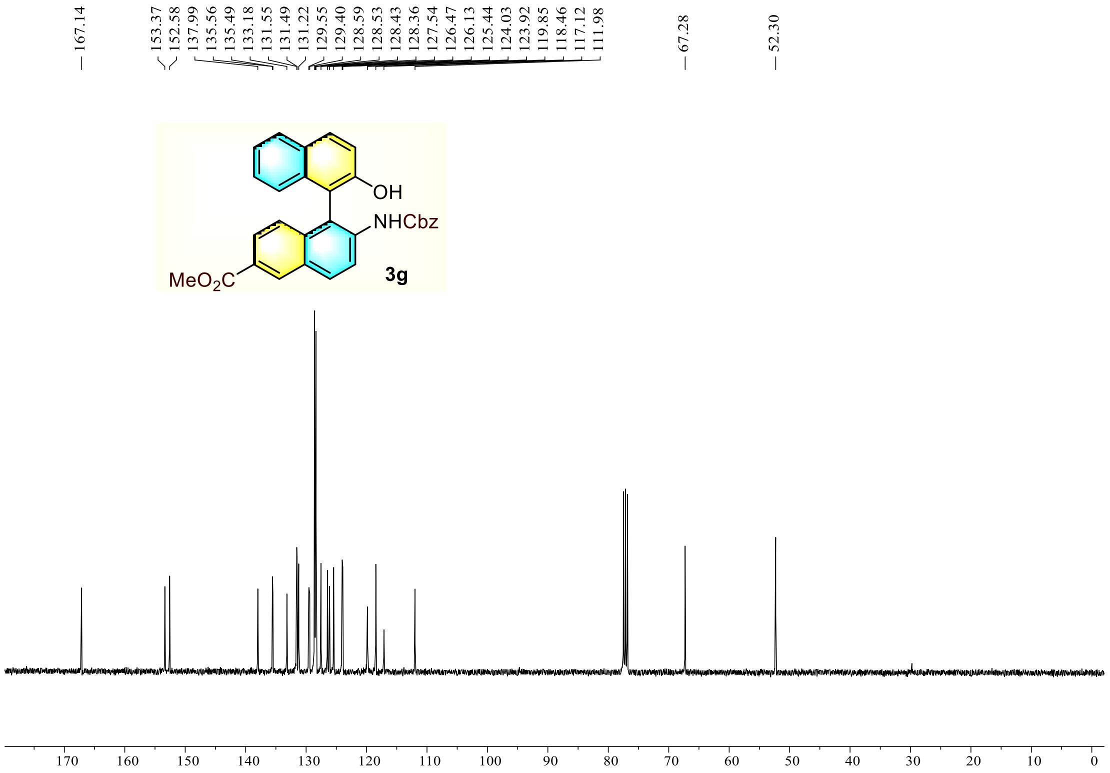

167.14
153.37
152.58
137.99
135.56
135.49
133.18
131.55
131.49
131.22
129.55
129.40
128.59
128.53
128.43
128.36
127.54
126.47
126.13
125.44
124.03
123.92
119.85
118.46
117.12
111.98
67.28
52.30
OH
NHCbz
MeO2C
3g
170 160 150 140 130 120 110 100 90 80 70 60 50 40 30 20 10 0

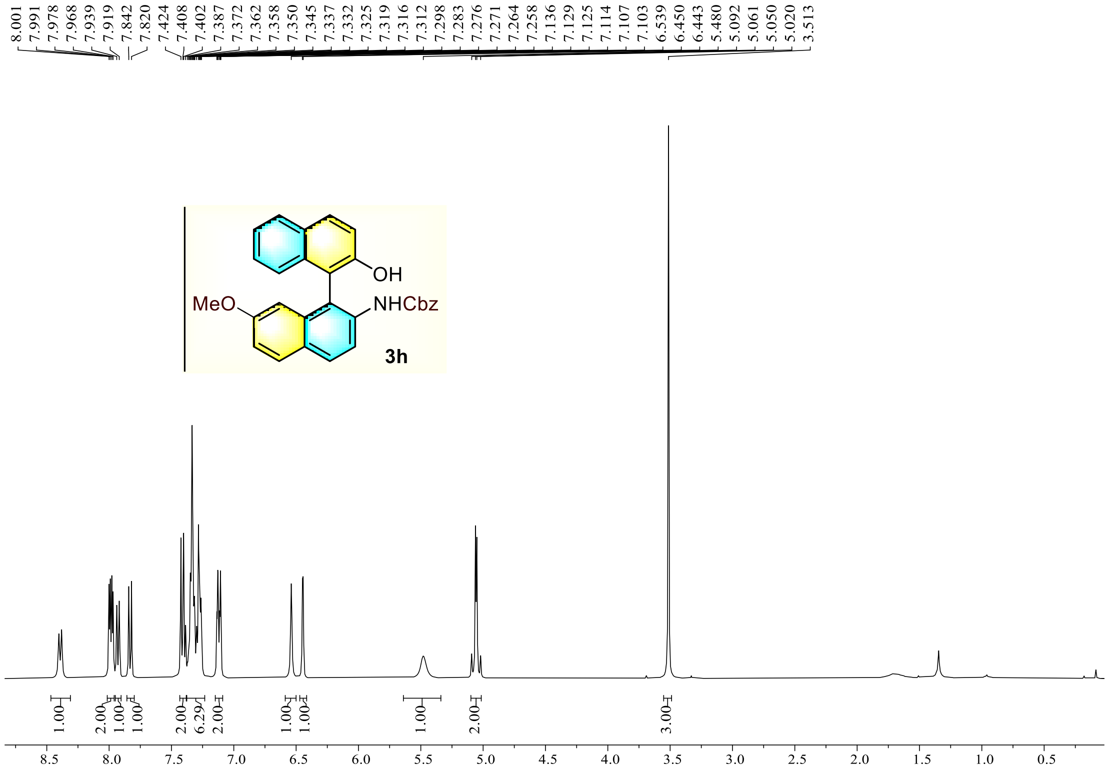

8.001
7.991
7.978
7.968
7.939
7.919
7.842
7.820
7.424
7.408
7.402
7.387
7.372
7.362
7.358
7.350
7.345
7.337
7.332
7.325
7.319
7.316
7.312
7.298
7.283
7.276
7.271
7.264
7.258
7.136
7.129
7.125
7.114
7.107
7.103
6.539
6.450
6.443
5.480
5.092
5.061
5.050
5.020
3.513
OH
MeO
NHCbz
3h
1.00
2.00
1.00
1.00
2.00
6.29
2.00
1.00
1.00
1.00
2.00
3.00
8.5 8.0 7.5 7.0 6.5 6.0 5.5 5.0 4.5 4.0 3.5 3.0 2.5 2.0 1.5 1.0 0.5

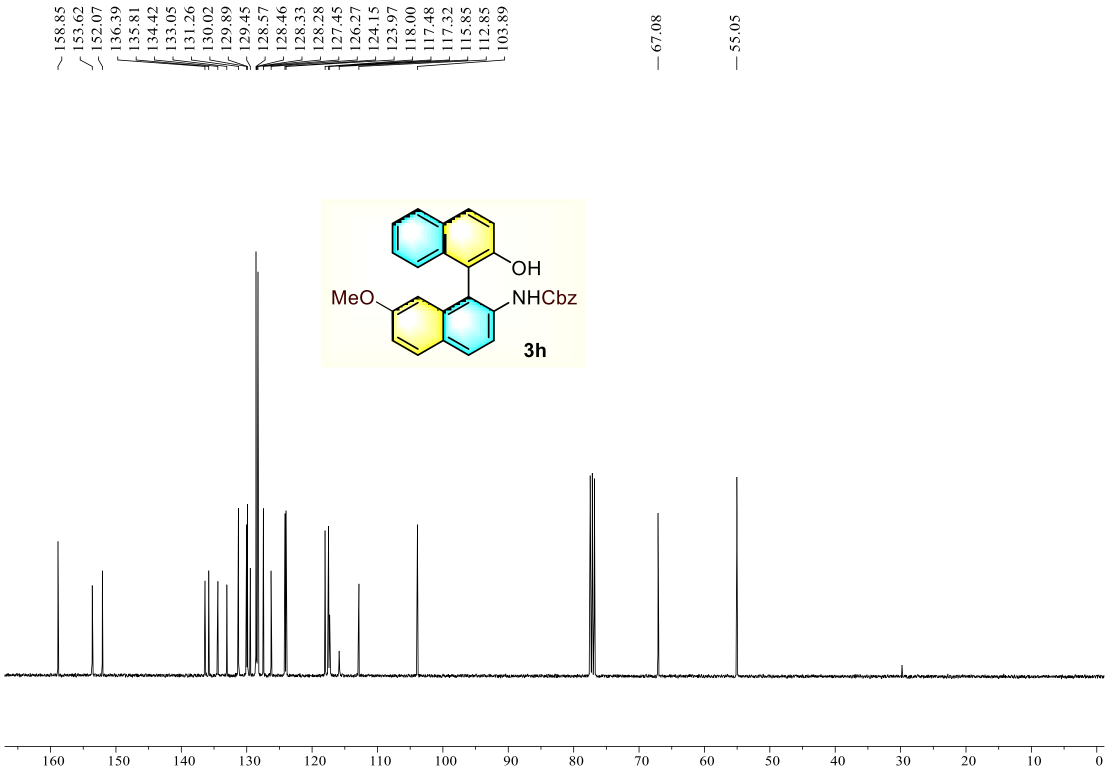
158.85
153.62
152.07
136.39
135.81
134.42
133.05
131.26
130.02
129.89
129.45
128.57
128.46
128.33
128.28
127.45
126.27
124.15
123.97
118.00
117.48
117.32
115.85
112.85
103.89
67.08
55.05
OH
MeO
NHCbz
3h
160 150 140 130 120 110 100 90 80 70 60 50 40 30 20 10 0

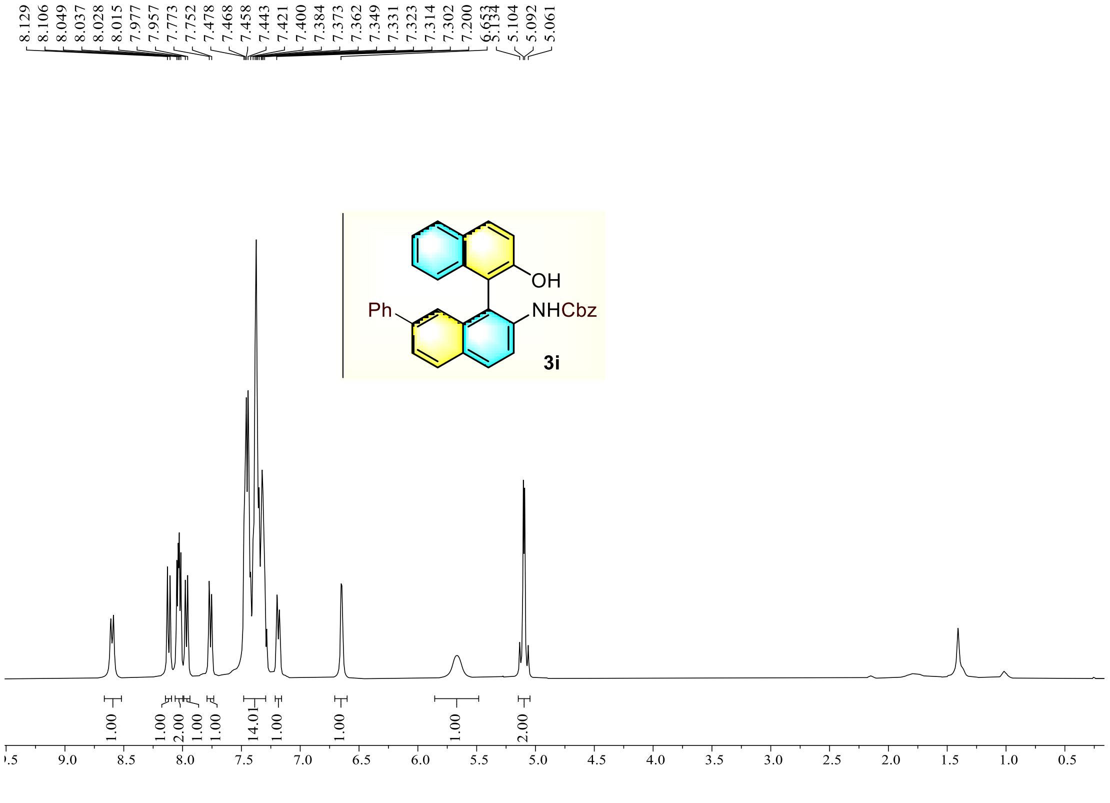
8.129
8.106
8.049
8.037
8.028
8.015
7.977
7.957
7.773
7.752
7.478
7.468
7.458
7.443
7.421
7.400
7.384
7.373
7.362
7.349
7.331
7.323
7.314
7.302
7.200
5.104
5.092
5.061
OH
Ph
NHCbz
3i
1.00
1.00
2.00
1.00
1.00
14.01
1.00
1.00
1.00
2.00
9.0 8.5 8.0 7.5 7.0 6.5 6.0 5.5 5.0 4.5 4.0 3.5 3.0 2.5 2.0 1.5 1.0 0.5

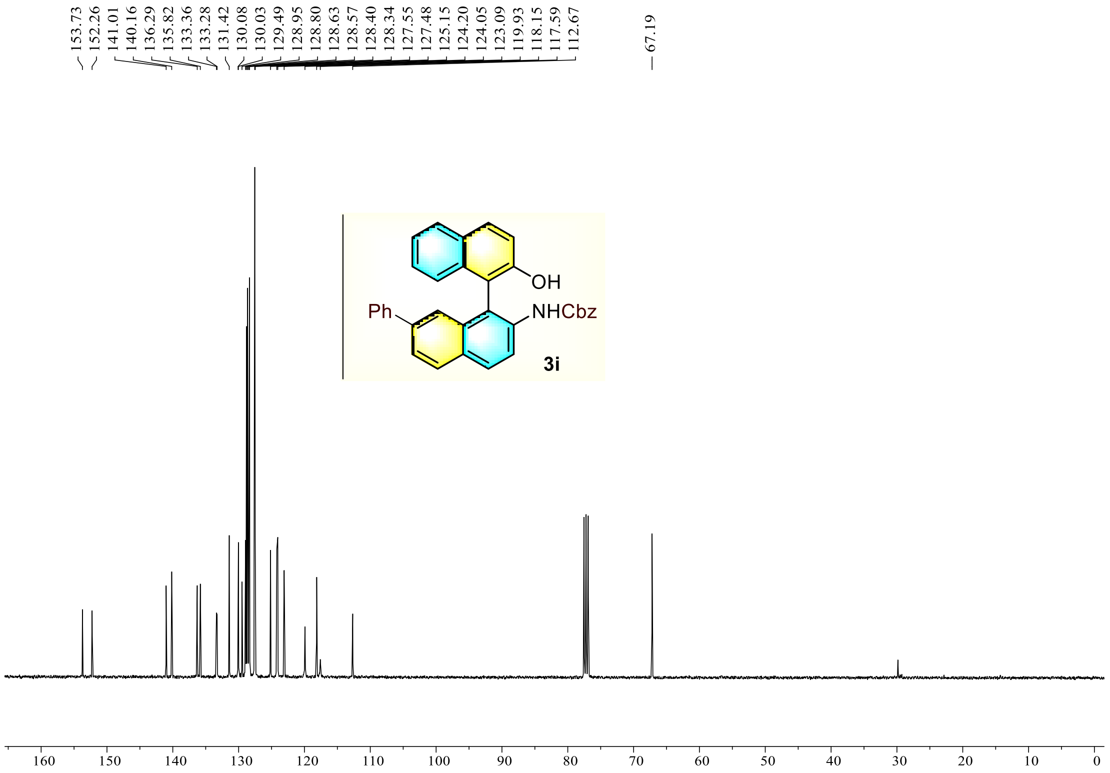
153.73
152.26
141.01
140.16
136.29
135.82
133.36
133.28
131.42
130.08
130.03
129.49
128.95
128.80
128.63
128.57
128.40
128.34
127.55
127.48
125.15
124.20
124.05
123.09
119.93
118.15
117.59
112.67
67.19
OH
Ph
NHCbz
3i
160 150 140 130 120 110 100 90 80 70 60 50 40 30 20 10 0

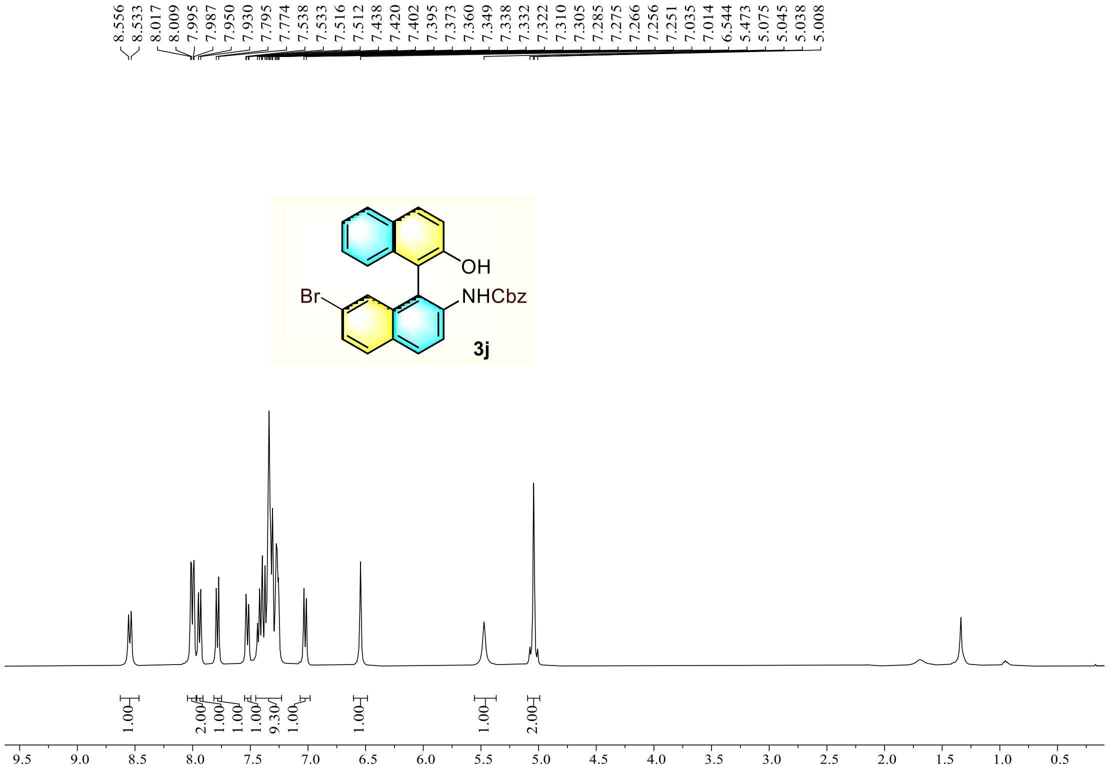
8.556
8.533
8.017
8.009
7.995
7.987
7.950
7.930
7.795
7.774
7.538
7.533
7.516
7.512
7.438
7.420
7.402
7.395
7.373
7.360
7.349
7.338
7.332
7.322
7.310
7.305
7.285
7.275
7.266
7.256
7.251
7.035
7.014
6.544
5.473
5.075
5.045
5.038
5.008
OH
Br
NHCbz
3j
1.00
2.00
1.00
1.00
1.00
9.30
1.00
1.00
1.00
2.00
9.5 9.0 8.5 8.0 7.5 7.0 6.5 6.0 5.5 5.0 4.5 4.0 3.5 3.0 2.5 2.0 1.5 1.0 0.5

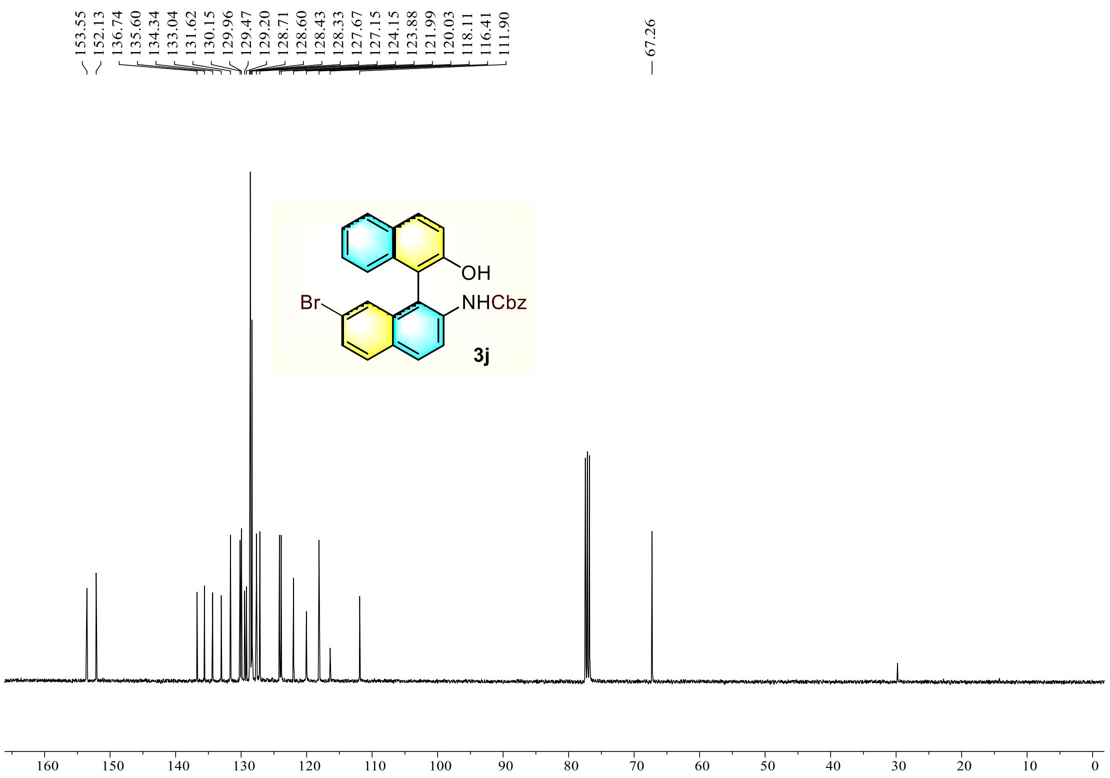
153.55
152.13
136.74
135.60
134.34
133.04
131.62
130.15
129.96
129.47
129.20
128.71
128.60
128.43
128.33
127.67
127.15
124.15
123.88
121.99
120.03
118.11
116.41
111.90
67.26
OH
Br
NHCbz
3j
160
150
140
130
120
110
100
90
80
70
60
50
40
30
20
10
0

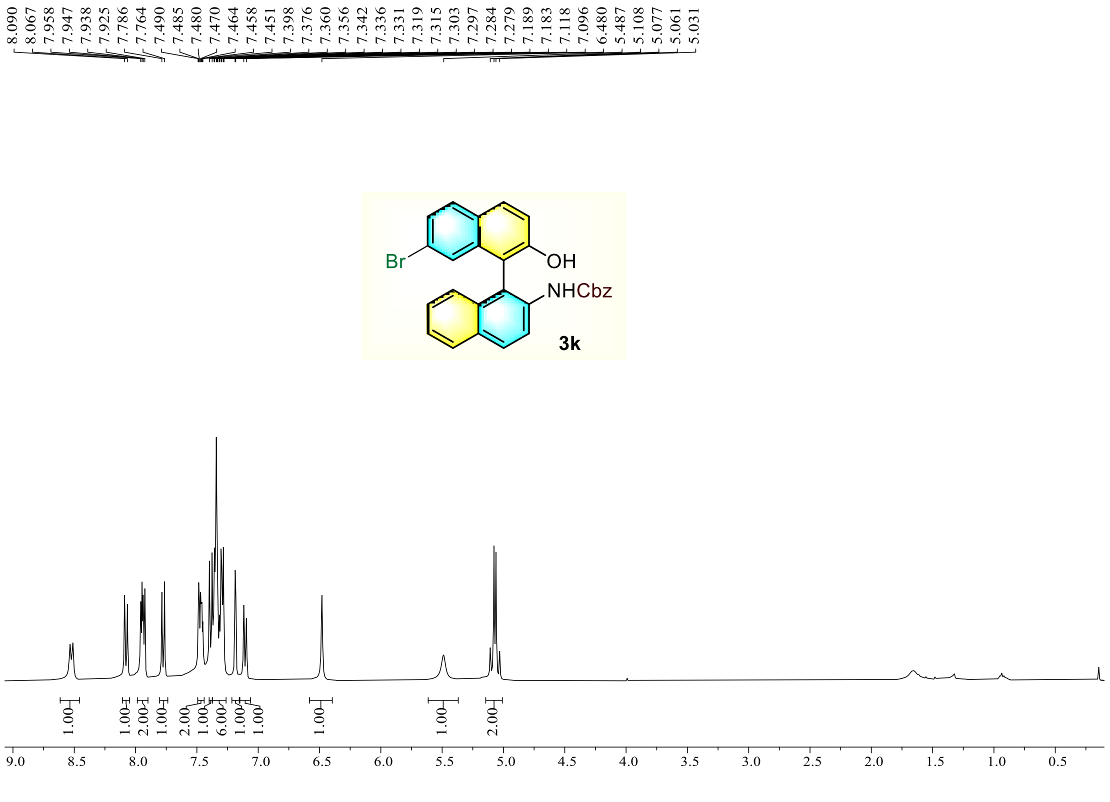
8.090
8.067
7.958
7.947
7.938
7.925
7.786
7.764
7.490
7.485
7.480
7.470
7.464
7.458
7.451
7.398
7.376
7.360
7.356
7.342
7.336
7.331
7.319
7.315
7.303
7.297
7.284
7.279
7.189
7.183
7.118
7.096
6.480
5.487
5.108
5.077
5.061
5.031
Br
OH
NHCbz
3k
1.00
1.00
2.00
1.00
2.00
1.00
6.00
1.00
1.00
1.00
1.00
2.00
9.0
8.5
8.0
7.5
7.0
6.5
6.0
5.5
5.0
4.5
4.0
3.5
3.0
2.5
2.0
1.5
1.0
0.5

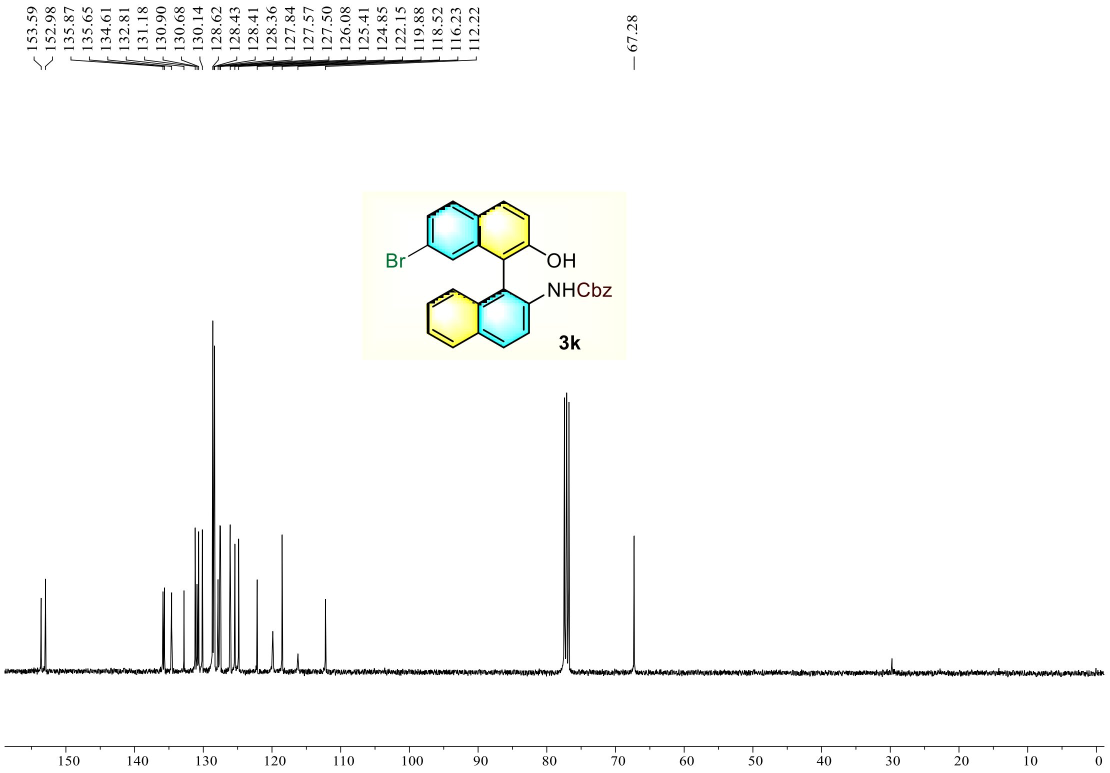
153.59
152.98
135.87
135.65
134.61
132.81
131.18
130.90
130.68
130.14
128.62
128.43
128.41
128.36
127.84
127.57
127.50
126.08
125.41
124.85
122.15
119.88
118.52
116.23
112.22
67.28
Br
OH
NHCbz
3k

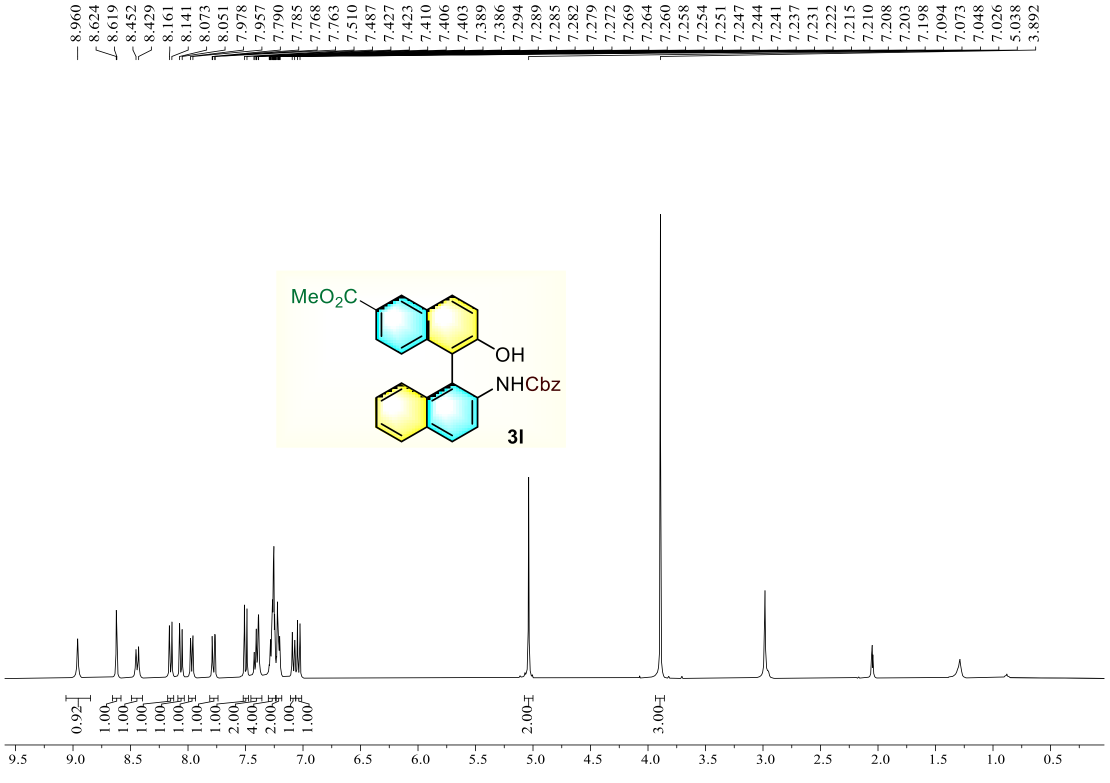
8.960
8.624
8.619
8.452
8.429
8.161
8.141
8.073
8.051
7.978
7.957
7.790
7.785
7.768
7.763
7.510
7.487
7.427
7.423
7.410
7.406
7.403
7.389
7.386
7.294
7.289
7.285
7.282
7.279
7.272
7.269
7.264
7.260
7.258
7.254
7.251
7.247
7.244
7.241
7.237
7.231
7.222
7.215
7.210
7.208
7.203
7.198
7.094
7.073
7.048
7.026
5.038
3.892
MeO_2C
OH
NHCbz
3l

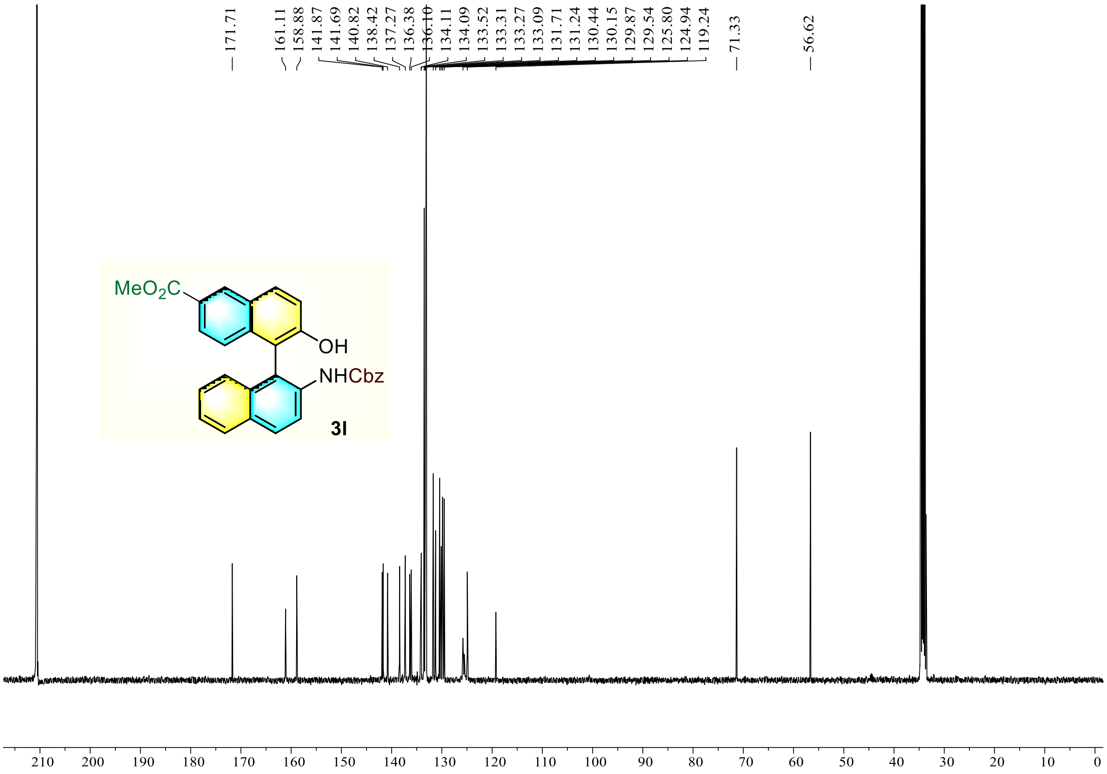
171.71
161.11
158.88
141.87
141.69
140.82
138.42
137.27
136.38
136.10
134.11
134.09
133.52
133.31
133.27
133.09
131.71
131.24
130.44
130.15
129.87
129.54
125.80
124.94
119.24
71.33
56.62
MeO2C
OH
NHCbz
3l

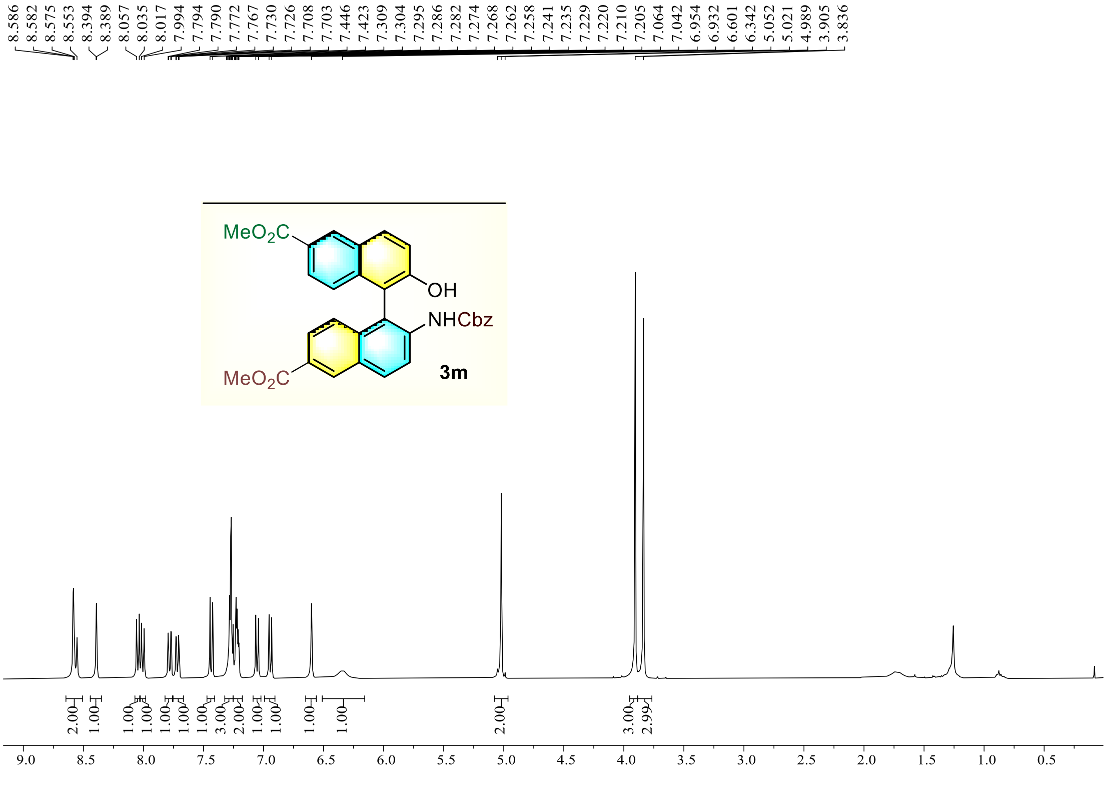
8.586
8.582
8.575
8.553
8.394
8.389
8.057
8.035
8.017
7.994
7.794
7.790
7.772
7.767
7.730
7.726
7.708
7.703
7.446
7.423
7.309
7.304
7.295
7.286
7.282
7.274
7.268
7.262
7.258
7.241
7.235
7.229
7.220
7.210
7.205
7.064
7.042
6.954
6.932
6.601
6.342
5.052
5.021
4.989
3.905
3.836
MeO2C
OH
NHCbz
MeO2C
3m
2.00
1.00
1.00
1.00
1.00
1.00
1.00
3.00
2.00
1.00
1.00
1.00
1.00
2.00
3.00
2.99

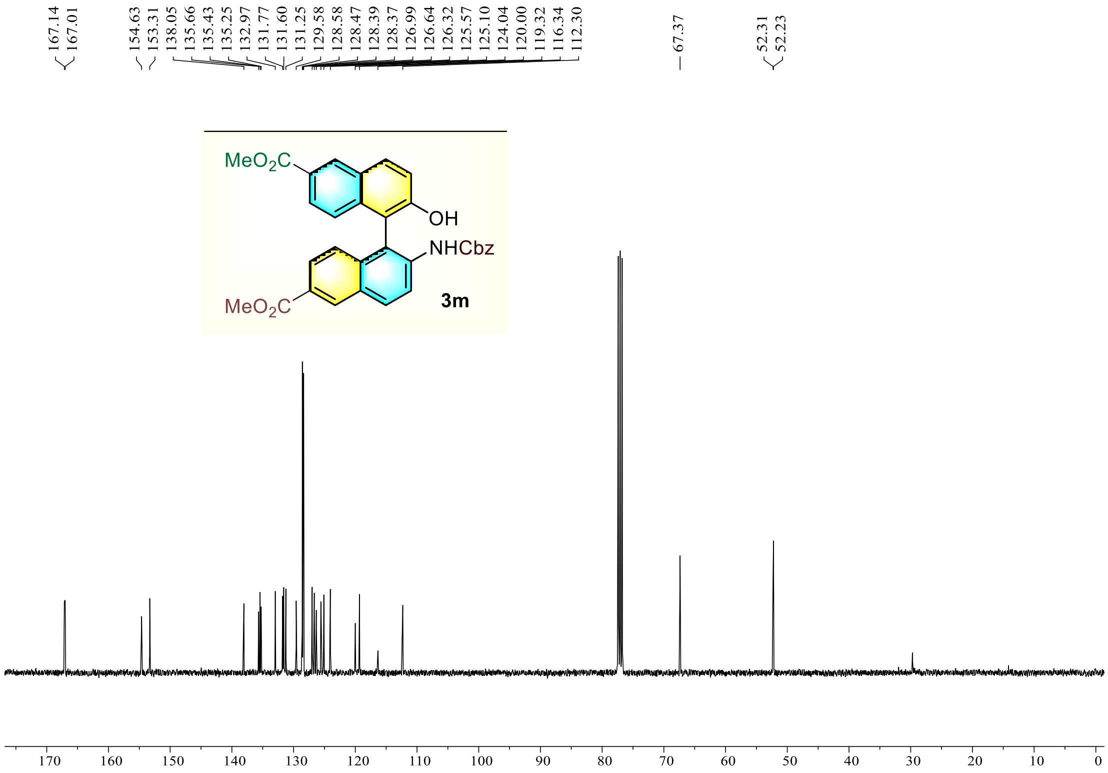
167.14
167.01
154.63
153.31
138.05
135.66
135.43
135.25
132.97
131.77
131.60
131.25
129.58
128.58
128.47
128.39
128.37
126.99
126.64
126.32
125.57
125.10
124.04
120.00
119.32
116.34
112.30
67.37
52.31
52.23
MeO2C
OH
NHCbz
MeO2C
3m
170
160
150
140
130
120
110
100
90
80
70
60
50
40
30
20
10
0

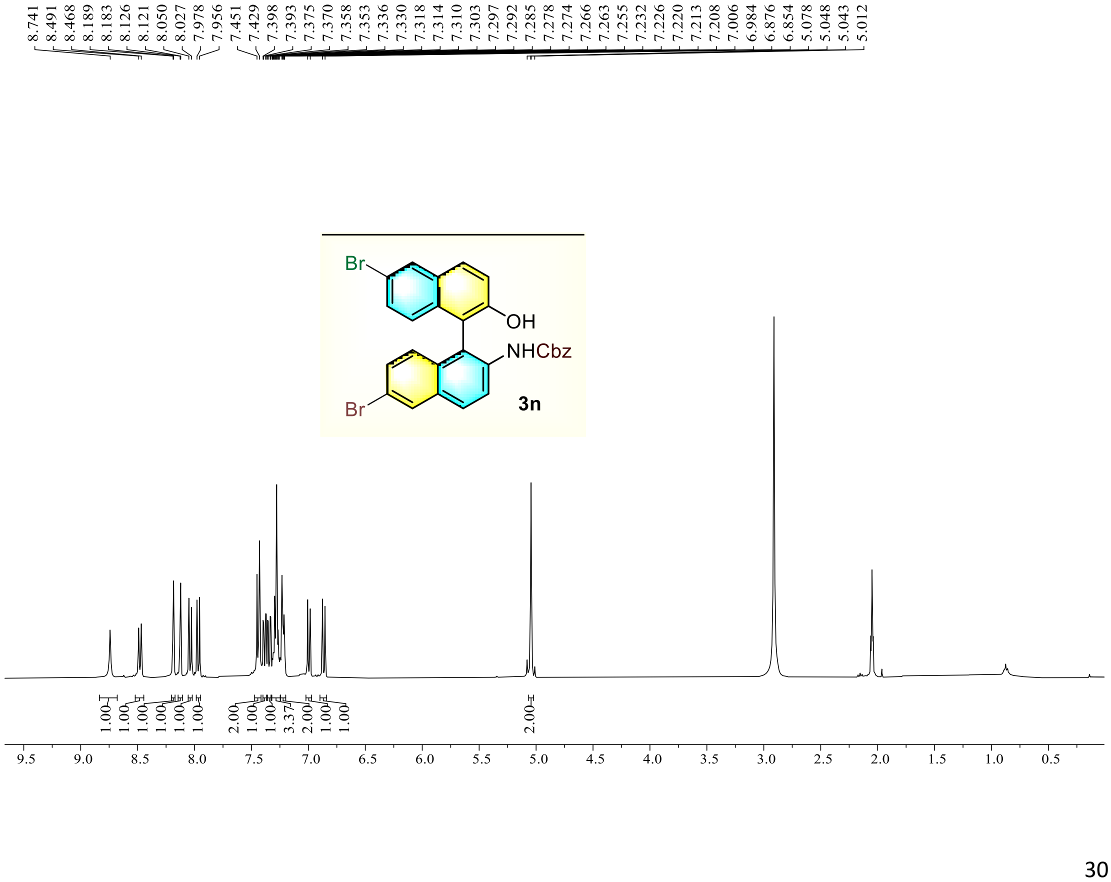
8.741
8.491
8.468
8.189
8.183
8.126
8.121
8.050
8.027
7.978
7.956
7.451
7.429
7.398
7.393
7.375
7.370
7.358
7.353
7.336
7.330
7.318
7.314
7.310
7.303
7.297
7.292
7.285
7.278
7.274
7.266
7.263
7.255
7.232
7.226
7.220
7.213
7.208
7.006
6.984
6.876
6.854
5.078
5.048
5.043
5.012
Br
OH
NHCbz
Br
3n
1.00
1.00
1.00
1.00
1.00
2.00
1.00
1.00
3.37
2.00
1.00
1.00
2.00
9.5
9.0
8.5
8.0
7.5
7.0
6.5
6.0
5.5
5.0
4.5
4.0
3.5
3.0
2.5
2.0
1.5
1.0
0.5

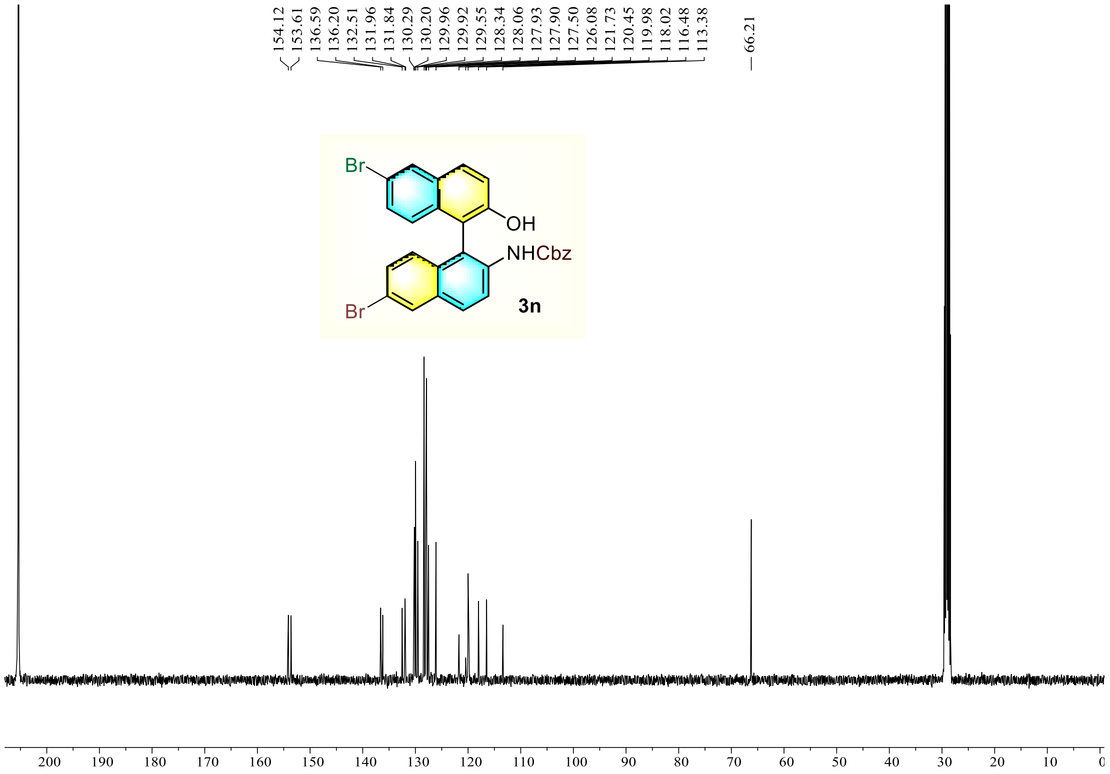
154.12
153.61
136.59
136.20
132.51
131.96
131.84
130.29
130.20
129.96
129.92
129.55
128.34
128.06
127.93
127.90
127.50
126.08
121.73
120.45
119.98
118.02
116.48
113.38
66.21
Br
OH
NHCbz
Br
3n
200 190 180 170 160 150 140 130 120 110 100 90 80 70 60 50 40 30 20 10 0

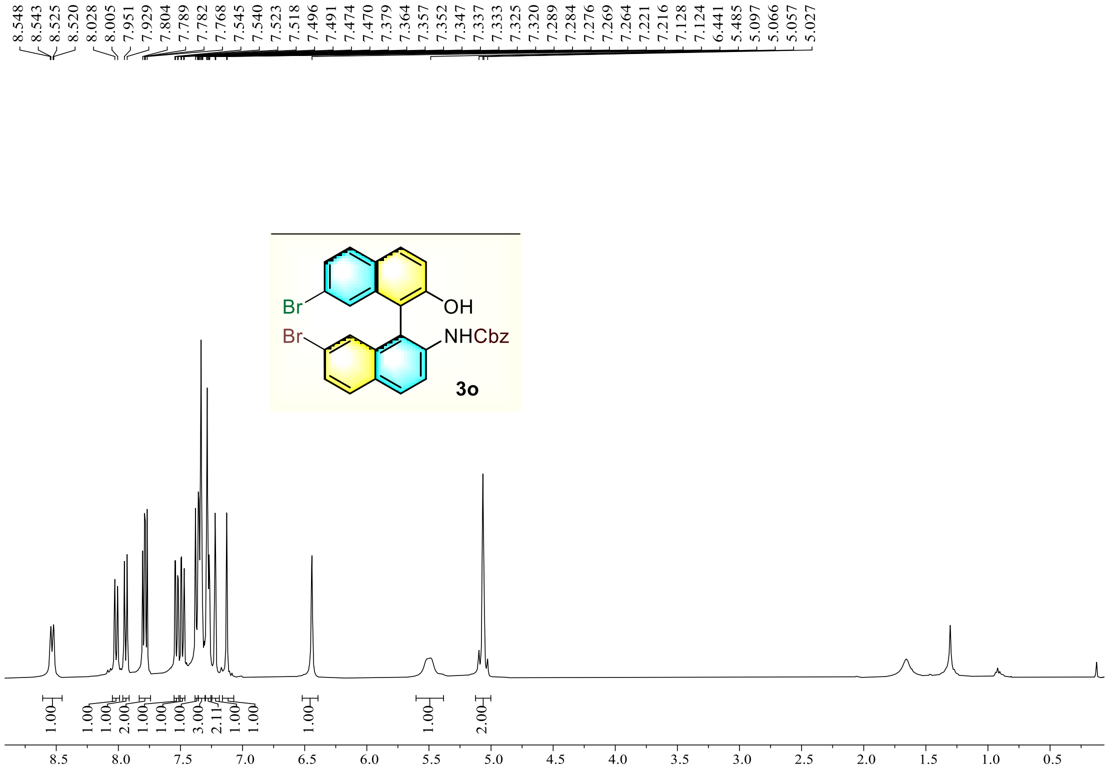
8.548
8.543
8.525
8.520
8.028
8.005
7.951
7.929
7.804
7.789
7.782
7.768
7.545
7.540
7.523
7.518
7.496
7.491
7.474
7.470
7.379
7.364
7.357
7.352
7.347
7.337
7.333
7.325
7.320
7.289
7.284
7.276
7.269
7.264
7.221
7.216
7.128
7.124
6.441
5.485
5.097
5.066
5.057
5.027
Br
OH
Br
NHCbz
3o
1.00
1.00
1.00
2.00
1.00
1.00
1.00
3.00
2.11
1.00
1.00
1.00
1.00
2.00
8.5 8.0 7.5 7.0 6.5 6.0 5.5 5.0 4.5 4.0 3.5 3.0 2.5 2.0 1.5 1.0 0.5

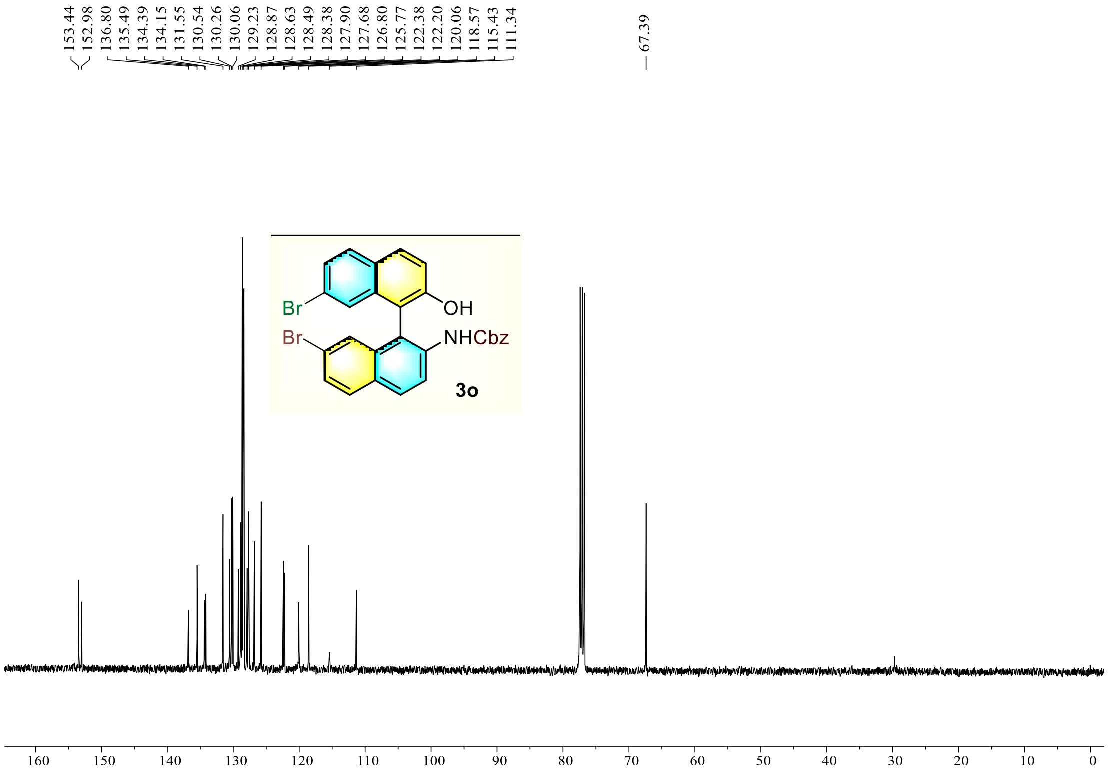
153.44
152.98
136.80
135.49
134.39
134.15
131.55
130.54
130.26
130.06
129.23
128.87
128.63
128.49
128.38
127.90
127.68
126.80
125.77
122.38
122.20
120.06
118.57
115.43
111.34
67.39
Br
Br
OH
NHCbz
3o
160 150 140 130 120 110 100 90 80 70 60 50 40 30 20 10 0

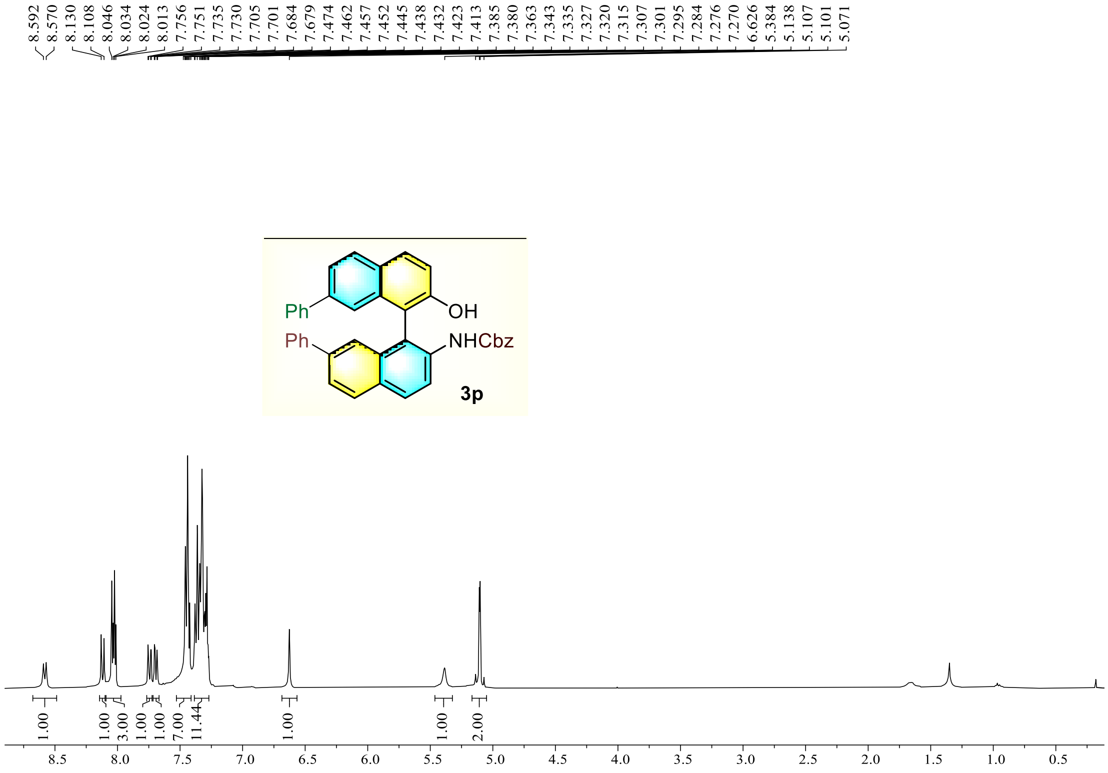
8.592
8.570
8.130
8.108
8.046
8.034
8.024
8.013
7.756
7.751
7.735
7.730
7.705
7.701
7.684
7.679
7.474
7.462
7.457
7.452
7.445
7.438
7.432
7.423
7.413
7.385
7.380
7.363
7.343
7.335
7.327
7.320
7.315
7.307
7.301
7.295
7.284
7.276
7.270
6.626
5.384
5.138
5.107
5.101
5.071
Ph
Ph
OH
NHCbz
3p
1.00
1.00
3.00
1.00
1.00
7.00
11.44
1.00
1.00
2.00
8.5 8.0 7.5 7.0 6.5 6.0 5.5 5.0 4.5 4.0 3.5 3.0 2.5 2.0 1.5 1.0 0.5

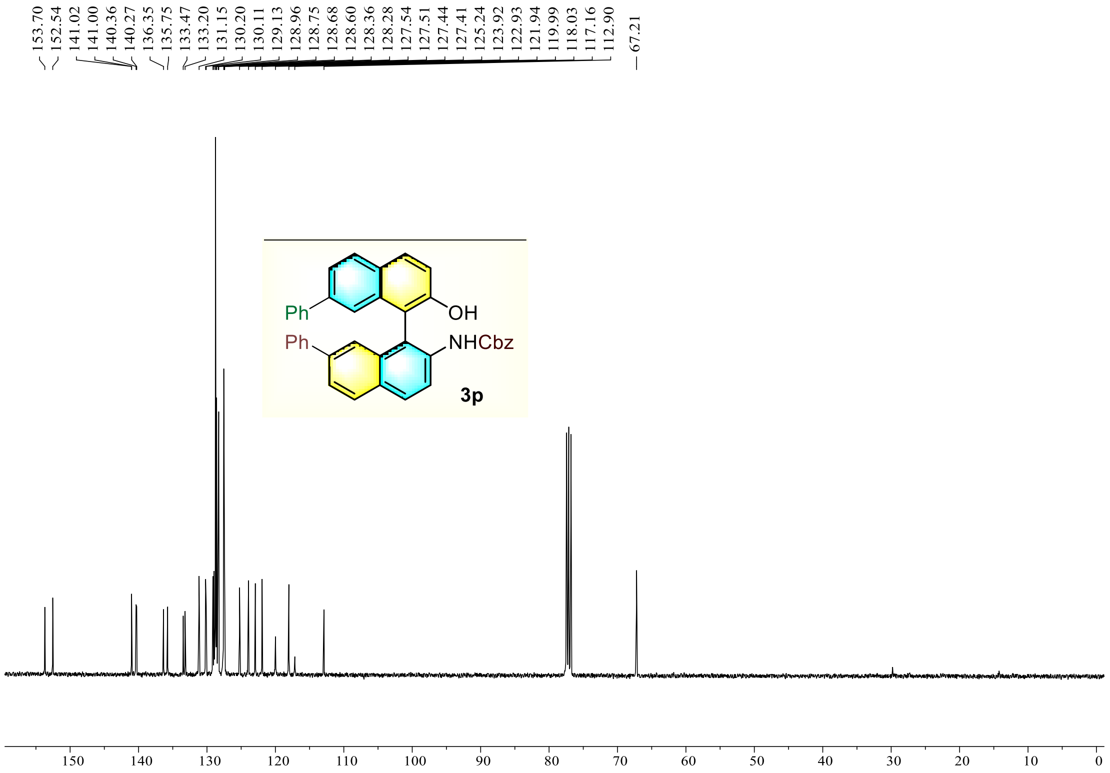
153.70
152.54
141.02
141.00
140.36
140.27
136.35
135.75
133.47
133.20
131.15
130.20
130.11
129.13
128.96
128.75
128.68
128.60
128.36
128.28
127.54
127.51
127.44
127.41
125.24
123.92
122.93
121.94
119.99
118.03
117.16
112.90
67.21
Ph
OH
Ph
NHCbz
3p
150
140
130
120
110
100
90
80
70
60
50
40
30
20
10
0

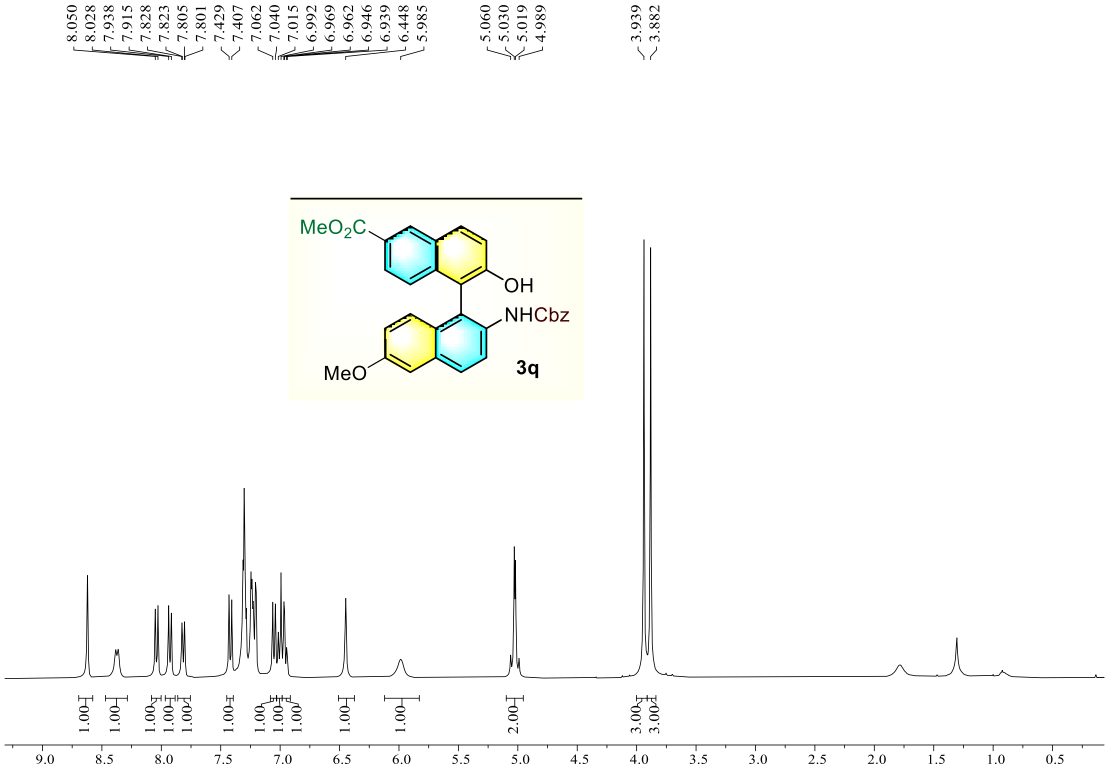
8.050
8.028
7.938
7.915
7.828
7.823
7.805
7.801
7.429
7.407
7.062
7.040
7.015
6.992
6.969
6.962
6.946
6.939
6.448
5.985
5.060
5.030
5.019
4.989
3.939
3.882
MeO2C
OH
NHCbz
MeO
3q
1.00
1.00
1.00
1.00
1.00
1.00
1.00
1.00
1.00
1.00
1.00
2.00
3.00
3.00
9.0
8.5
8.0
7.5
7.0
6.5
6.0
5.5
5.0
4.5
4.0
3.5
3.0
2.5
2.0
1.5
1.0
0.5

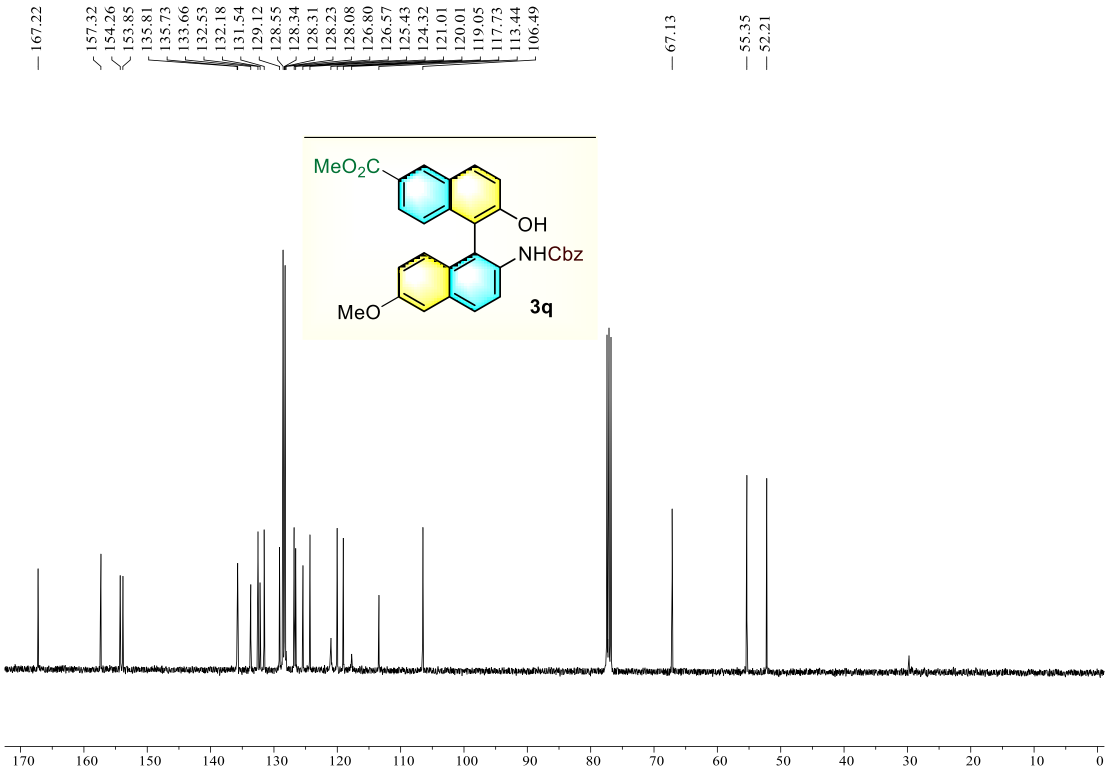

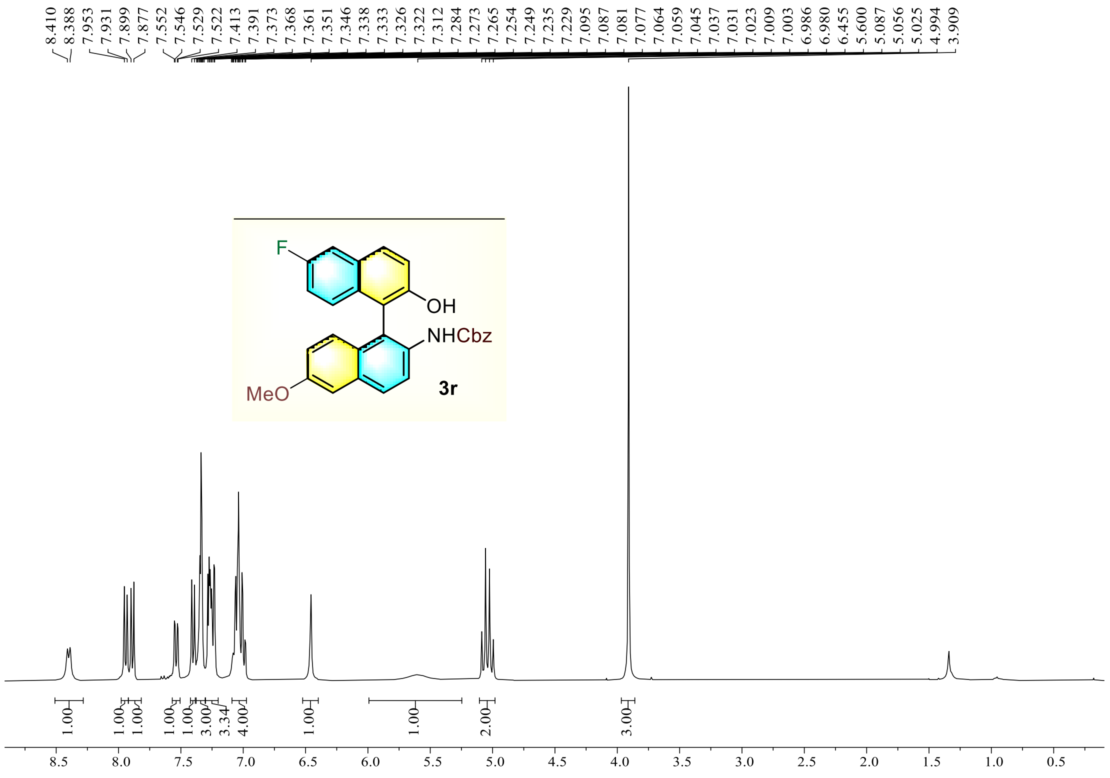

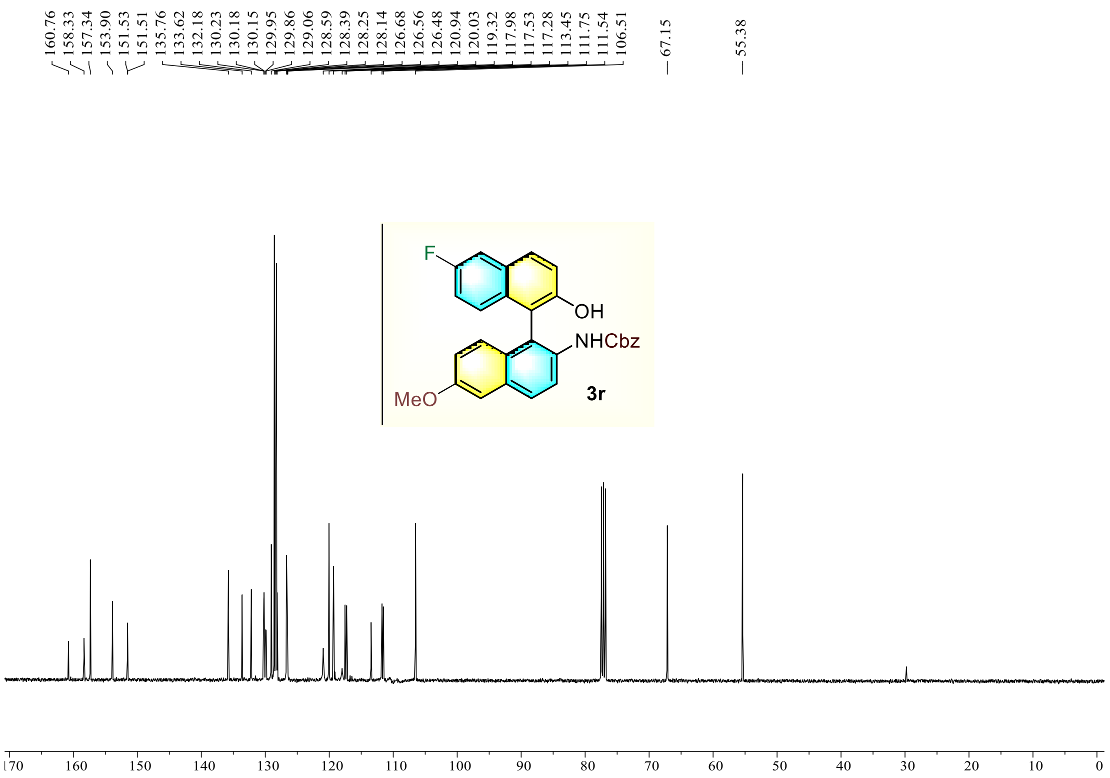

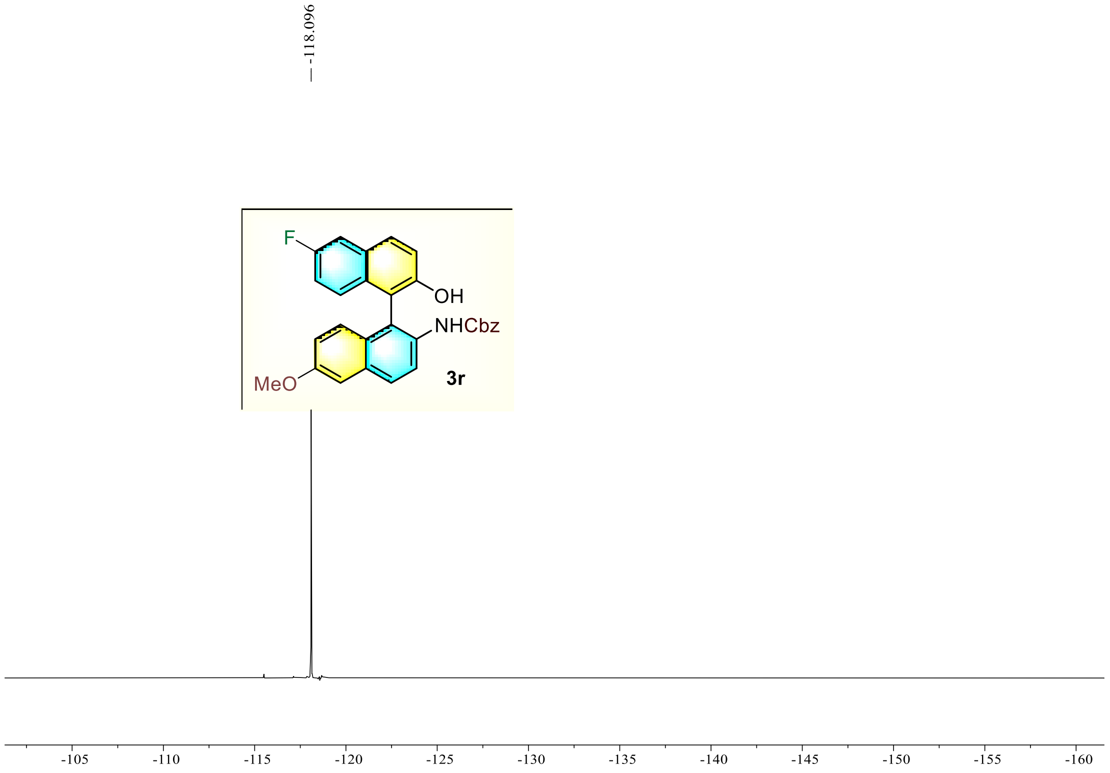

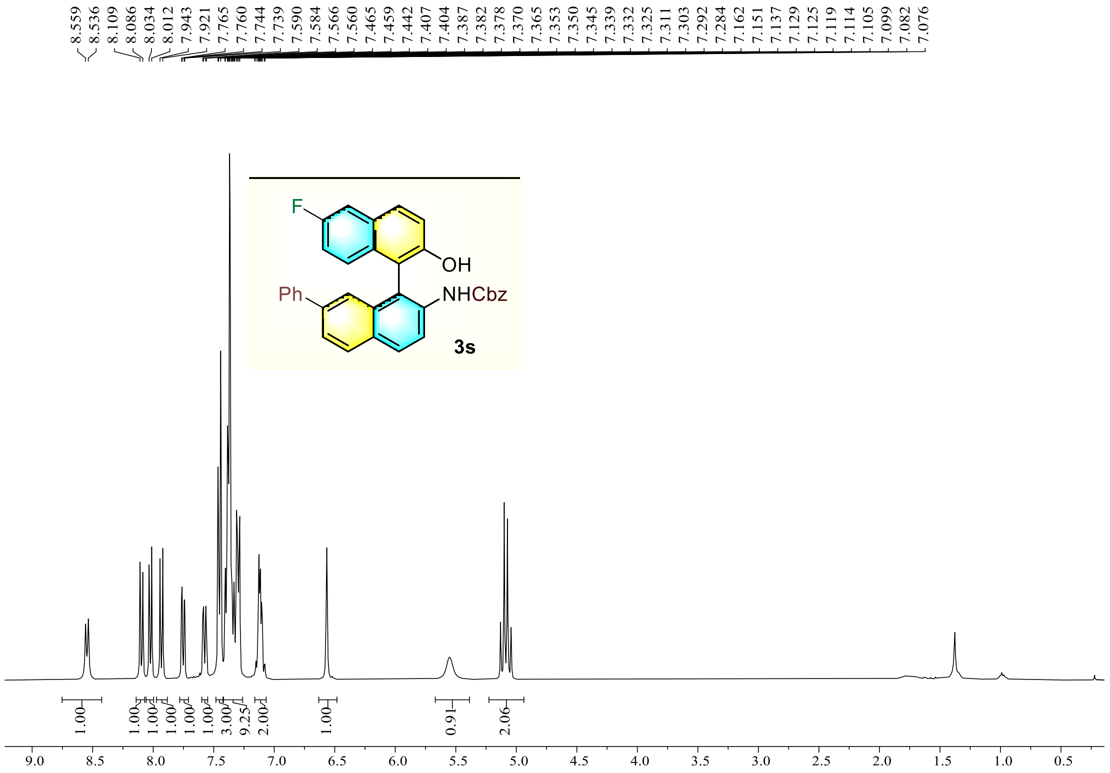
8.559
8.536
8.109
8.086
8.034
8.012
7.943
7.921
7.765
7.760
7.744
7.739
7.590
7.584
7.566
7.560
7.465
7.459
7.442
7.407
7.404
7.387
7.382
7.378
7.370
7.365
7.353
7.350
7.345
7.339
7.332
7.325
7.311
7.303
7.292
7.284
7.162
7.151
7.137
7.129
7.125
7.119
7.114
7.105
7.099
7.082
7.076
F
OH
Ph
NHCbz
3s
1.00
1.00
1.00
1.00
1.00
1.00
3.00
9.25
2.00
1.00
0.91
2.06
9.0 8.5 8.0 7.5 7.0 6.5 6.0 5.5 5.0 4.5 4.0 3.5 3.0 2.5 2.0 1.5 1.0 0.5

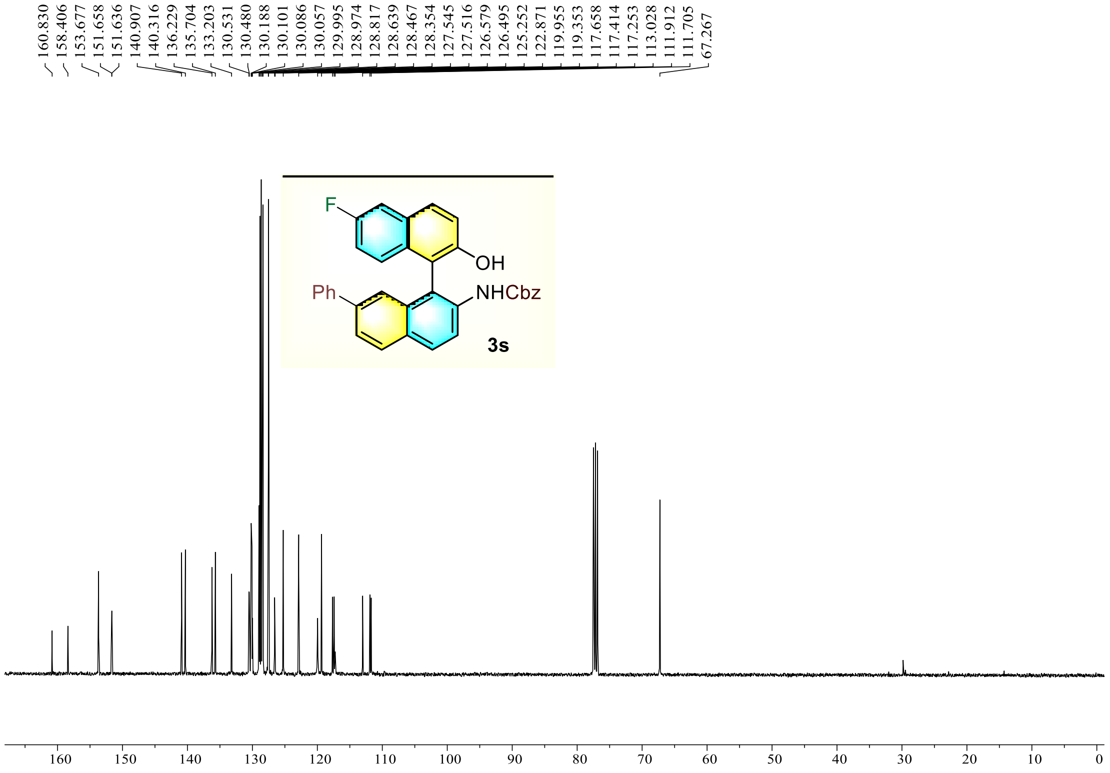
160.830
158.406
153.677
151.658
151.636
140.907
140.316
136.229
135.704
133.203
130.531
130.480
130.188
130.101
130.086
130.057
129.995
128.974
128.817
128.639
128.467
128.354
127.545
127.516
126.579
126.495
125.252
122.871
119.955
119.353
117.658
117.414
117.253
113.028
111.912
111.705
67.267
F
OH
Ph
NHCbz
3s
160 150 140 130 120 110 100 90 80 70 60 50 40 30 20 10 0

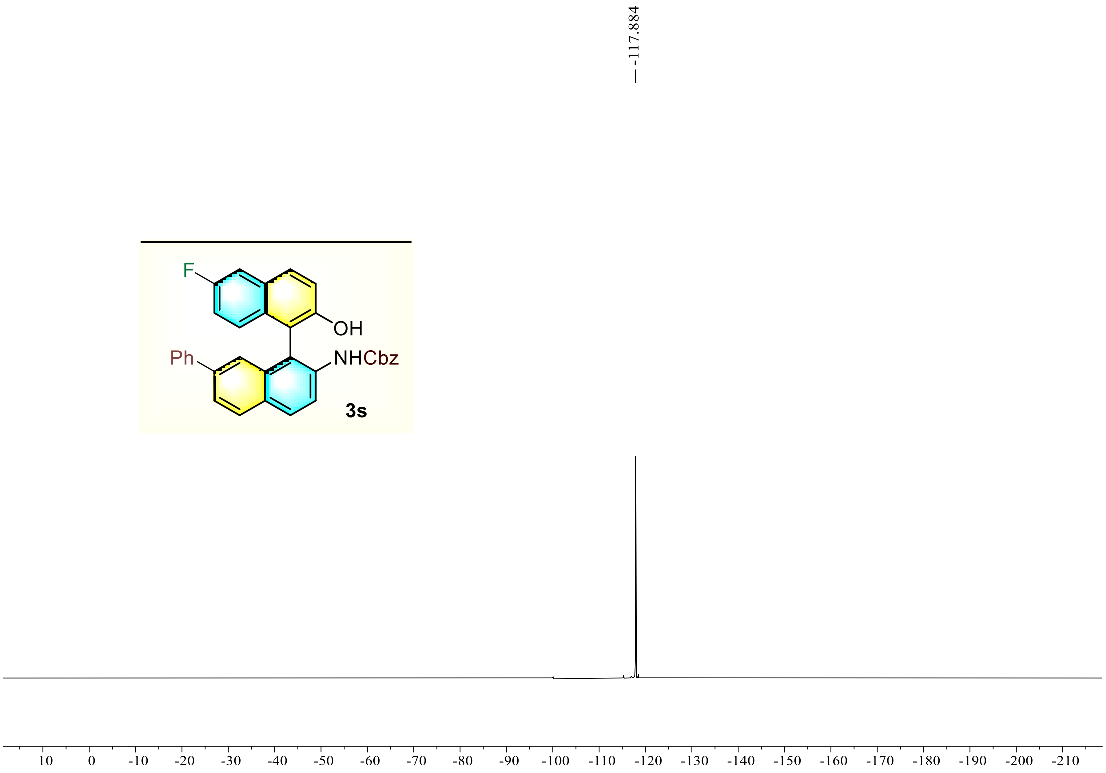
-117.884
F
OH
Ph
NHCbz
3s
10 0 -10 -20 -30 -40 -50 -60 -70 -80 -90 -100 -110 -120 -130 -140 -150 -160 -170 -180 -190 -200 -210

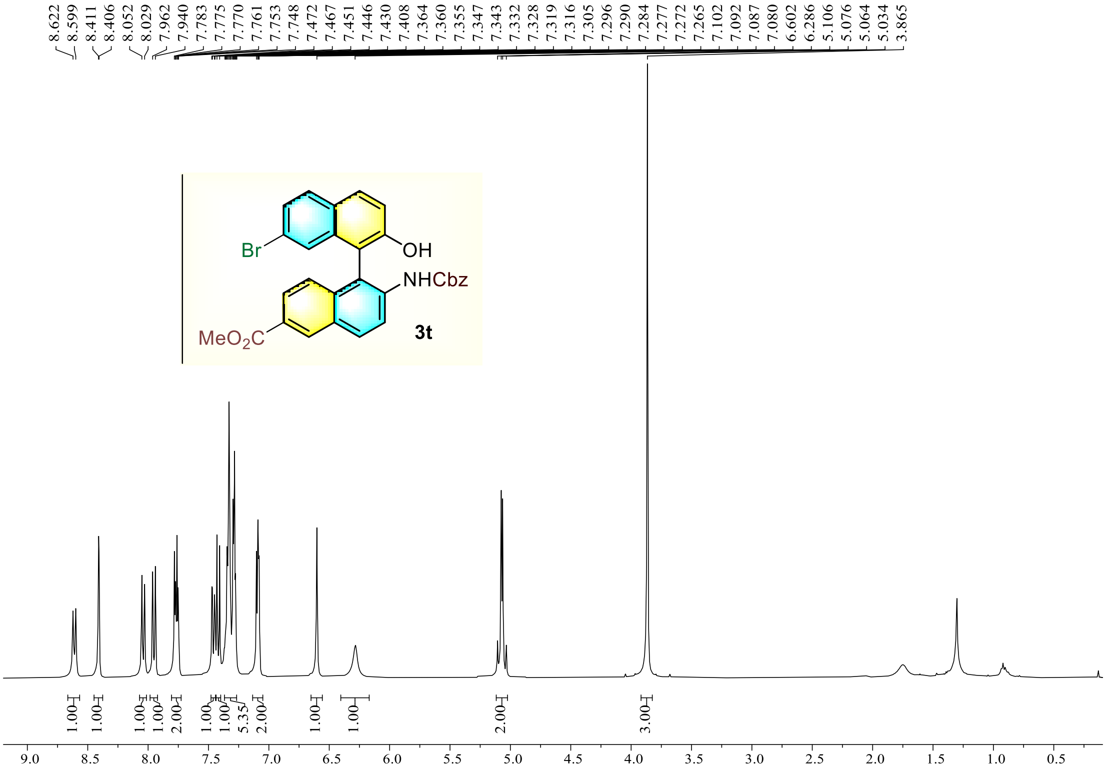
8.622 8.599 8.411 8.406 8.052 8.029 7.962 7.940 7.783 7.775 7.770 7.761 7.753 7.748 7.472 7.467 7.451 7.446 7.430 7.408 7.364 7.360 7.355 7.347 7.343 7.332 7.328 7.319 7.316 7.305 7.296 7.290 7.284 7.277 7.272 7.265 7.102 7.092 7.087 7.080 6.602 6.286 5.106 5.076 5.064 5.034 3.865
Br
OH
NHCbz
MeO2C
3t
1.00 1.00 1.00 1.00 2.00 1.00 1.00 5.35 2.00 1.00 1.00 2.00 3.00
9.0 8.5 8.0 7.5 7.0 6.5 6.0 5.5 5.0 4.5 4.0 3.5 3.0 2.5 2.0 1.5 1.0 0.5

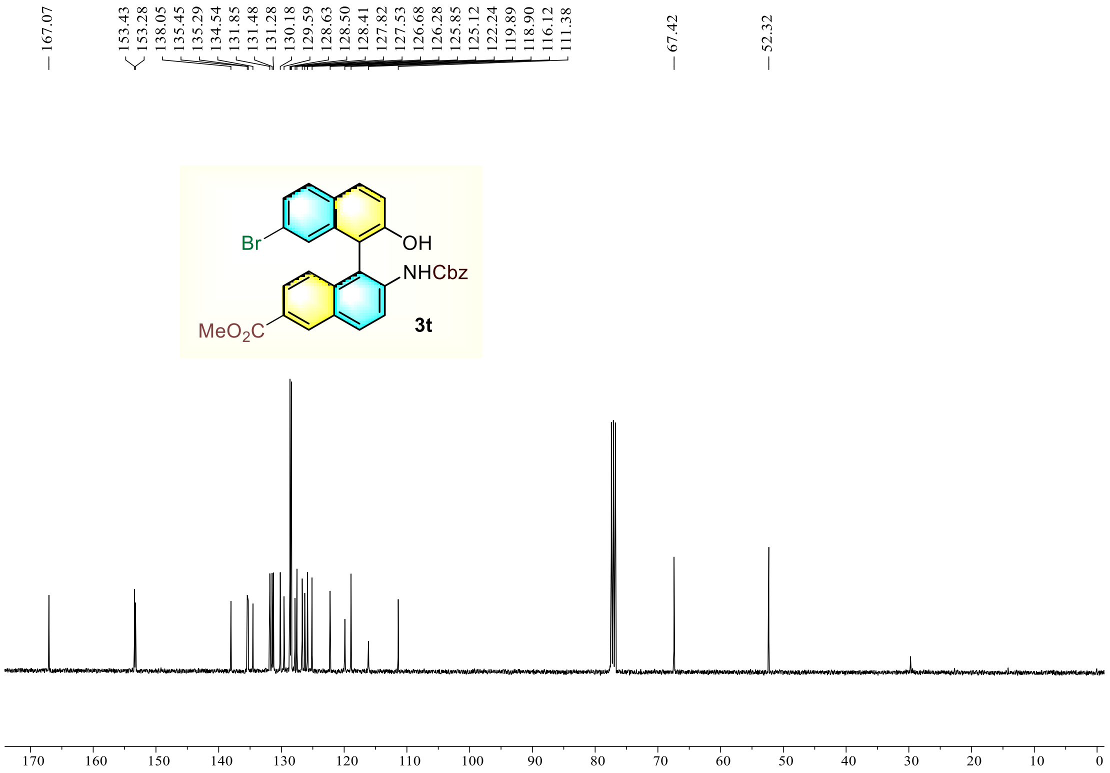
167.07
153.43
153.28
138.05
135.45
135.29
134.54
131.85
131.48
131.28
130.18
129.59
128.63
128.50
128.41
127.82
127.53
126.68
126.28
125.85
125.12
122.24
119.89
118.90
116.12
111.38
67.42
52.32
Br
OH
NHCbz
MeO_2C
3t
170
160
150
140
130
120
110
100
90
80
70
60
50
40
30
20
10
0

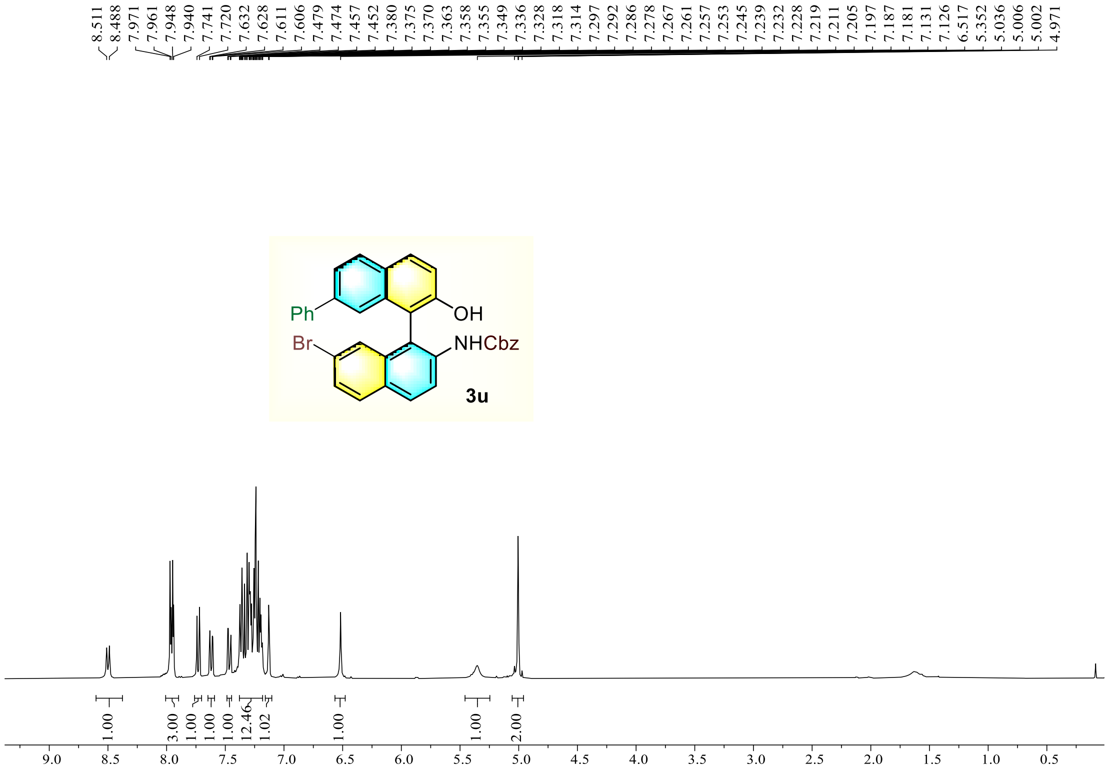
8.511
8.488
7.971
7.961
7.948
7.940
7.741
7.720
7.632
7.628
7.611
7.606
7.479
7.474
7.457
7.452
7.380
7.375
7.370
7.363
7.358
7.355
7.349
7.336
7.328
7.318
7.314
7.297
7.292
7.286
7.278
7.267
7.261
7.257
7.253
7.245
7.239
7.232
7.228
7.219
7.211
7.205
7.197
7.187
7.181
7.131
7.126
6.517
5.352
5.036
5.006
5.002
4.971
Ph
OH
Br
NHCbz
3u
1.00
3.00
1.00
1.00
1.00
12.46
1.02
1.00
1.00
2.00
9.0
8.5
8.0
7.5
7.0
6.5
6.0
5.5
5.0
4.5
4.0
3.5
3.0
2.5
2.0
1.5
1.0
0.5

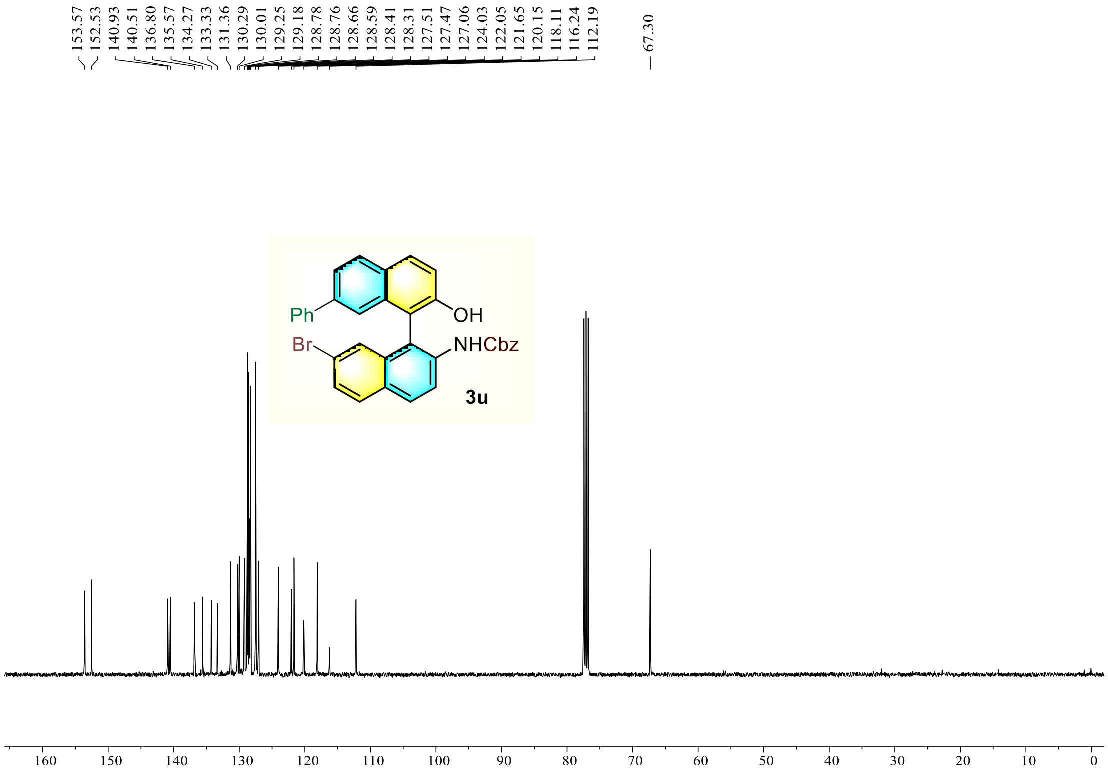
153.57
152.53
140.93
140.51
136.80
135.57
134.27
133.33
131.36
130.29
130.01
129.25
129.18
128.78
128.76
128.66
128.59
128.41
128.31
127.51
127.47
127.06
124.03
122.05
121.65
120.15
118.11
116.24
112.19
67.30
Ph
OH
Br
NHCbz
3u
160 150 140 130 120 110 100 90 80 70 60 50 40 30 20 10 0

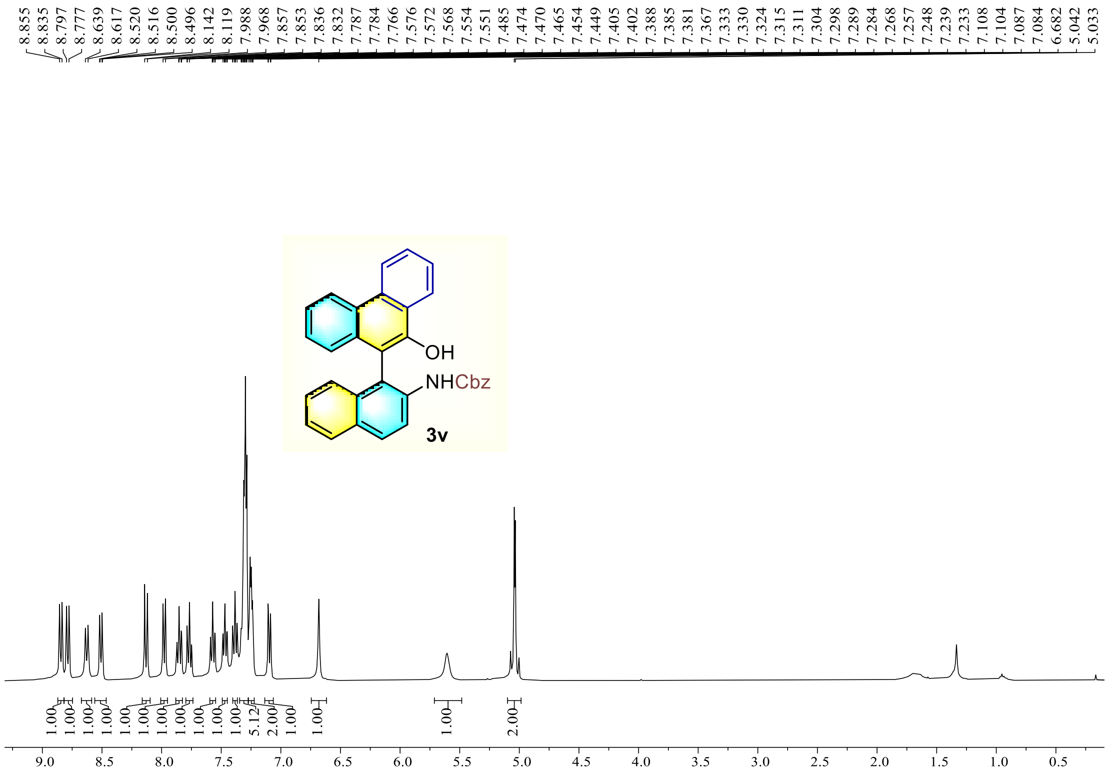
8.855
8.835
8.797
8.777
8.639
8.617
8.520
8.516
8.500
8.496
8.142
8.119
7.988
7.968
7.857
7.853
7.836
7.832
7.787
7.784
7.766
7.576
7.572
7.568
7.554
7.551
7.485
7.474
7.470
7.465
7.454
7.449
7.405
7.402
7.388
7.385
7.381
7.367
7.333
7.330
7.324
7.315
7.311
7.304
7.298
7.289
7.284
7.268
7.257
7.248
7.239
7.233
7.108
7.104
7.087
7.084
6.682
5.042
5.033
OH
NHCbz
3v
1.00
1.00
1.00
1.00
1.00
1.00
1.00
1.00
1.00
1.00
5.12
2.00
1.00
1.00
1.00
2.00
9.0 8.5 8.0 7.5 7.0 6.5 6.0 5.5 5.0 4.5 4.0 3.5 3.0 2.5 2.0 1.5 1.0 0.5

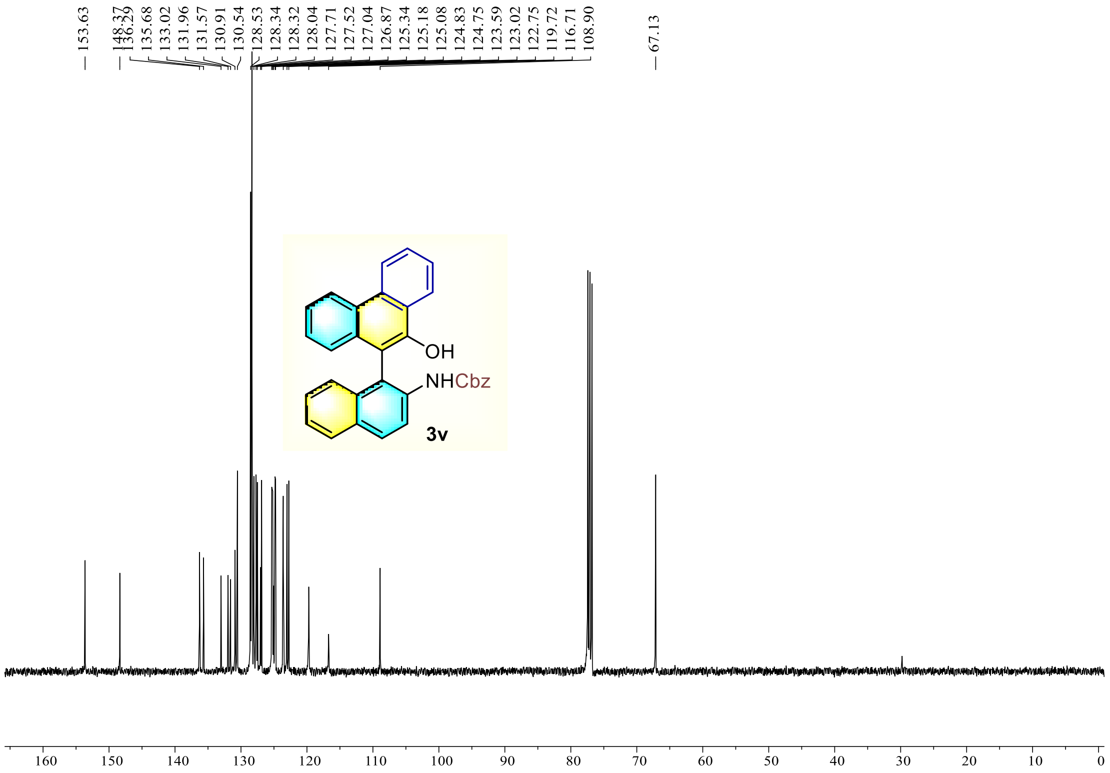
3v
OH
NHCbz

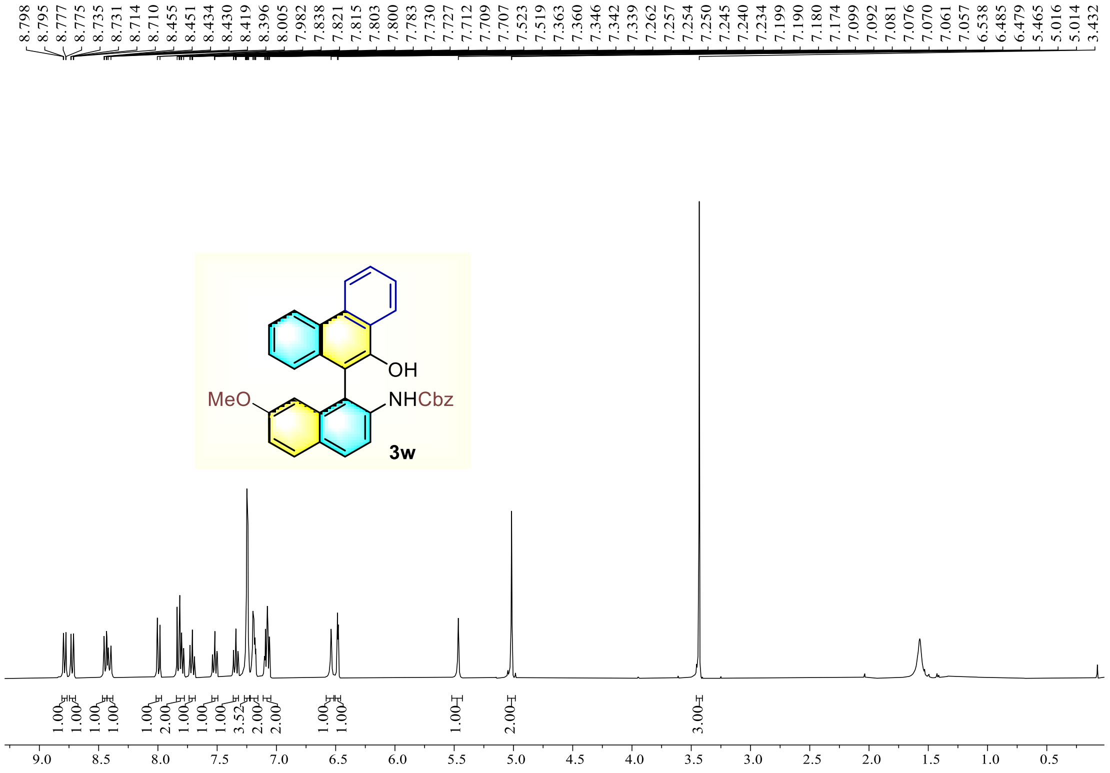
3w
OH
NHCbz
MeO

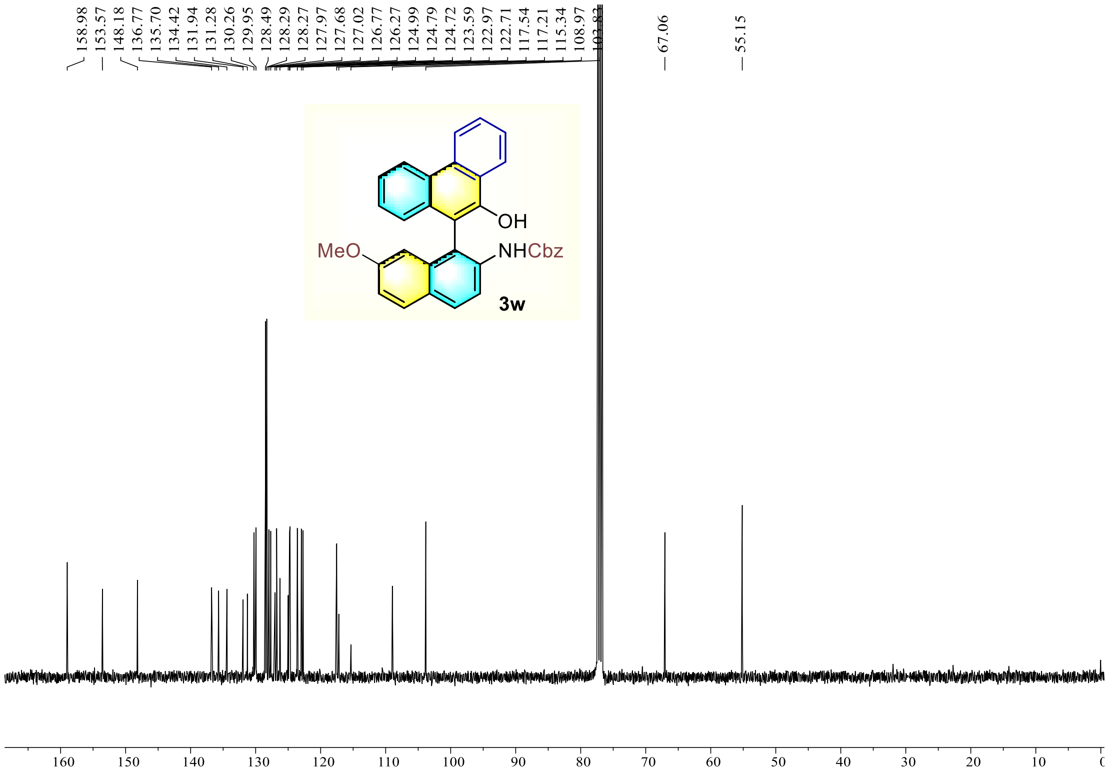
158.98
153.57
148.18
136.77
135.70
134.42
131.94
131.28
130.26
129.95
128.49
128.29
128.27
127.97
127.68
127.02
126.77
126.27
124.99
124.79
124.72
123.59
122.97
122.71
117.54
117.21
115.34
108.97
67.06
55.15
OH
MeO
NHCbz
3w
160
150
140
130
120
110
100
90
80
70
60
50
40
30
20
10

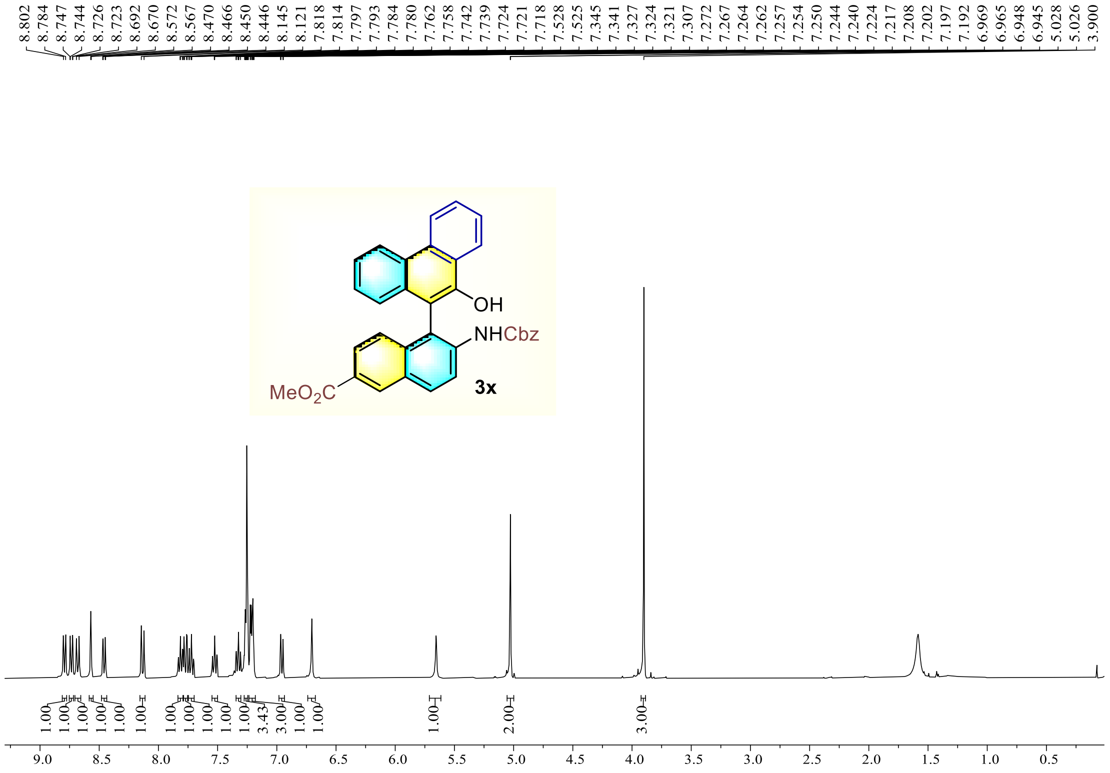
8.802
8.784
8.747
8.744
8.726
8.723
8.692
8.670
8.572
8.567
8.470
8.466
8.450
8.446
8.145
8.121
7.818
7.814
7.797
7.793
7.784
7.780
7.762
7.758
7.742
7.739
7.724
7.721
7.718
7.528
7.525
7.345
7.341
7.327
7.324
7.321
7.307
7.272
7.267
7.264
7.262
7.257
7.254
7.250
7.244
7.240
7.224
7.217
7.208
7.202
7.197
7.192
6.969
6.965
6.948
6.945
5.028
5.026
3.900
OH
NHCbz
MeO_2C
3x
1.00
1.00
1.00
1.00
1.00
1.00
1.00
1.00
1.00
3.43
3.00
1.00
1.00
1.00
2.00
3.00
9.0
8.5
8.0
7.5
7.0
6.5
6.0
5.5
5.0
4.5
4.0
3.5
3.0
2.5
2.0
1.5
1.0
0.5

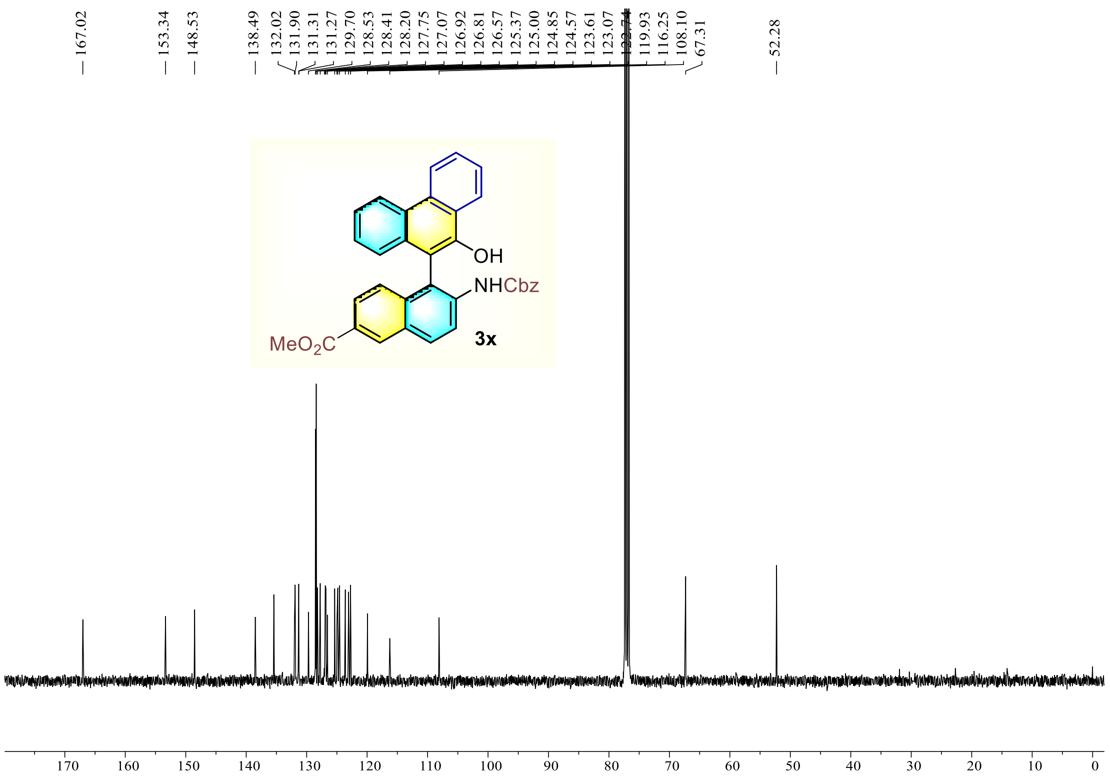

167.02
153.34
148.53
138.49
132.02
131.90
131.31
131.27
129.70
128.53
128.41
128.20
127.75
127.07
126.92
126.81
126.57
125.37
125.00
124.85
124.57
123.61
123.07
119.93
116.25
108.10
67.31
52.28
OH
NHCbz
MeO2C
3x
170 160 150 140 130 120 110 100 90 80 70 60 50 40 30 20 10 0

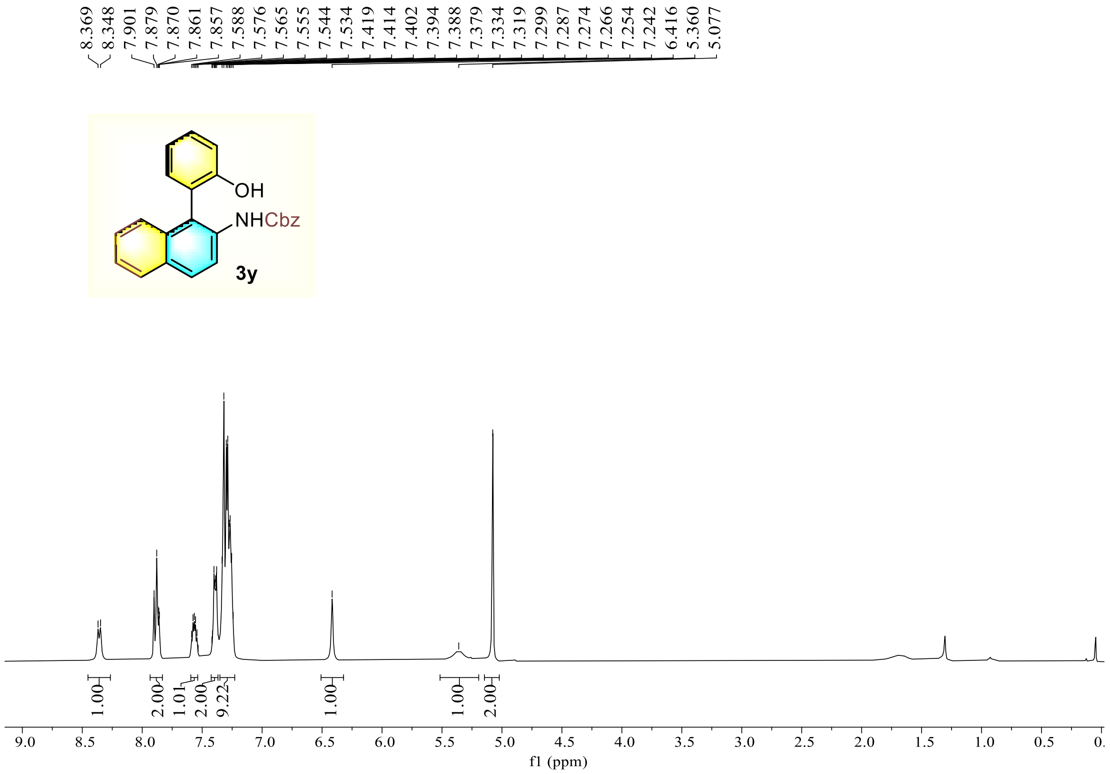

8.369
8.348
7.901
7.879
7.870
7.861
7.857
7.588
7.576
7.565
7.555
7.544
7.534
7.419
7.414
7.402
7.394
7.388
7.379
7.334
7.319
7.299
7.287
7.274
7.266
7.254
7.242
6.416
5.360
5.077
OH
NHCbz
3y
1.00
2.00
1.01
2.00
9.22
1.00
1.00
2.00
9.0 8.5 8.0 7.5 7.0 6.5 6.0 5.5 5.0 4.5 4.0 3.5 3.0 2.5 2.0 1.5 1.0 0.5 0.
f1 (ppm)

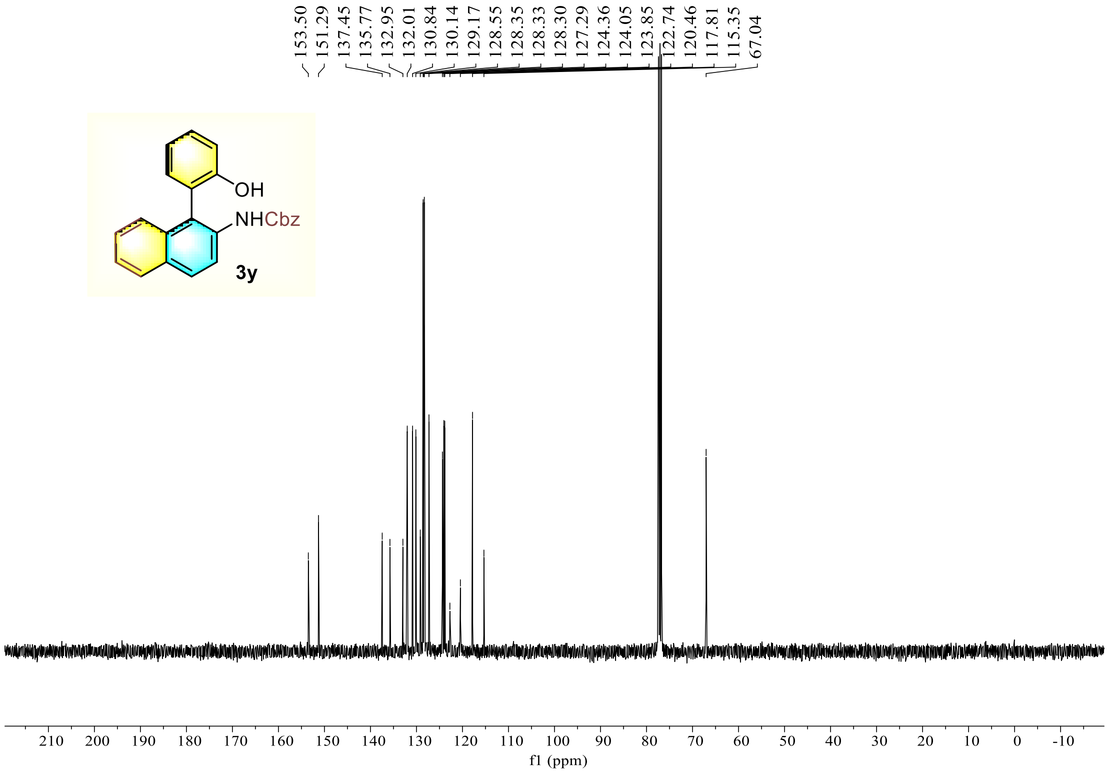

153.50
151.29
137.45
135.77
132.95
132.01
130.84
130.14
129.17
128.55
128.35
128.33
128.30
127.29
124.36
124.05
123.85
122.74
120.46
117.81
115.35
67.04
OH
NHCbz
3y
210 200 190 180 170 160 150 140 130 120 110 100 90 80 70 60 50 40 30 20 10 0 -10
f1 (ppm)

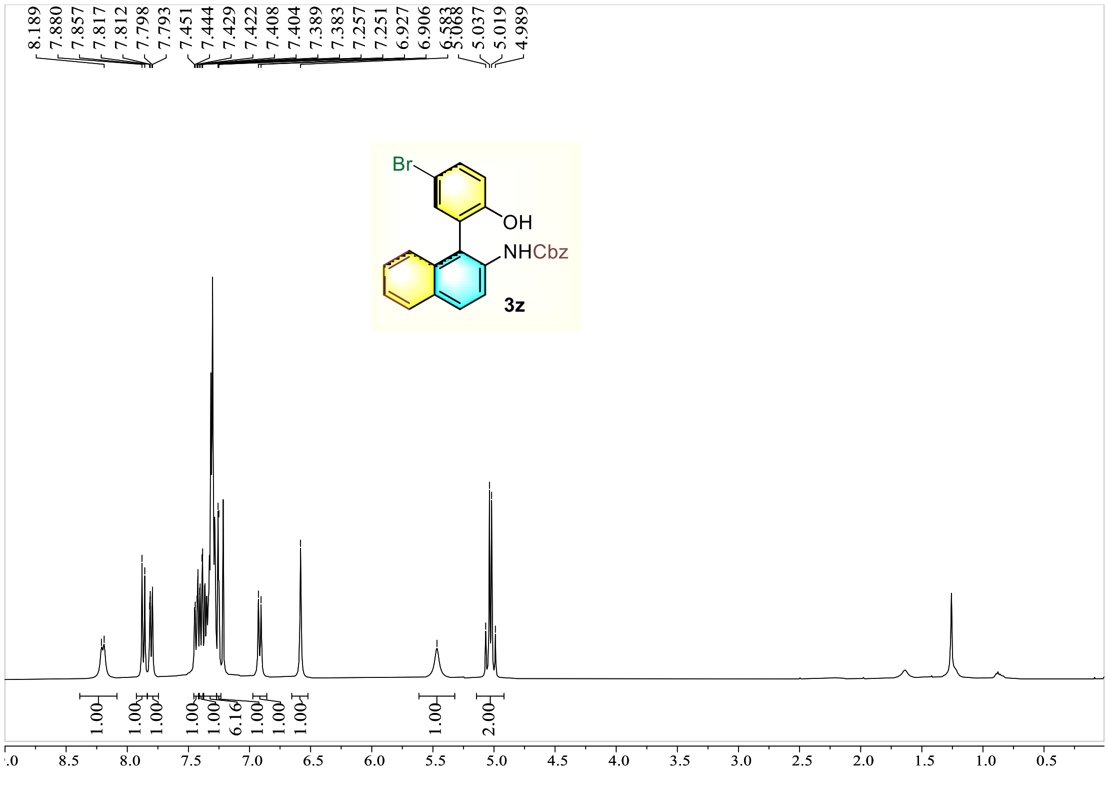

8.189
7.880
7.857
7.817
7.812
7.798
7.793
7.451
7.444
7.429
7.422
7.408
7.404
7.389
7.383
7.257
7.251
6.927
6.906
6.583
5.488
5.037
5.019
4.989
Br
OH
NHCbz
3z
1.00
1.00
1.00
1.00
1.00
6.16
1.00
1.00
1.00
1.00
2.00
8.5 8.0 7.5 7.0 6.5 6.0 5.5 5.0 4.5 4.0 3.5 3.0 2.5 2.0 1.5 1.0 0.5

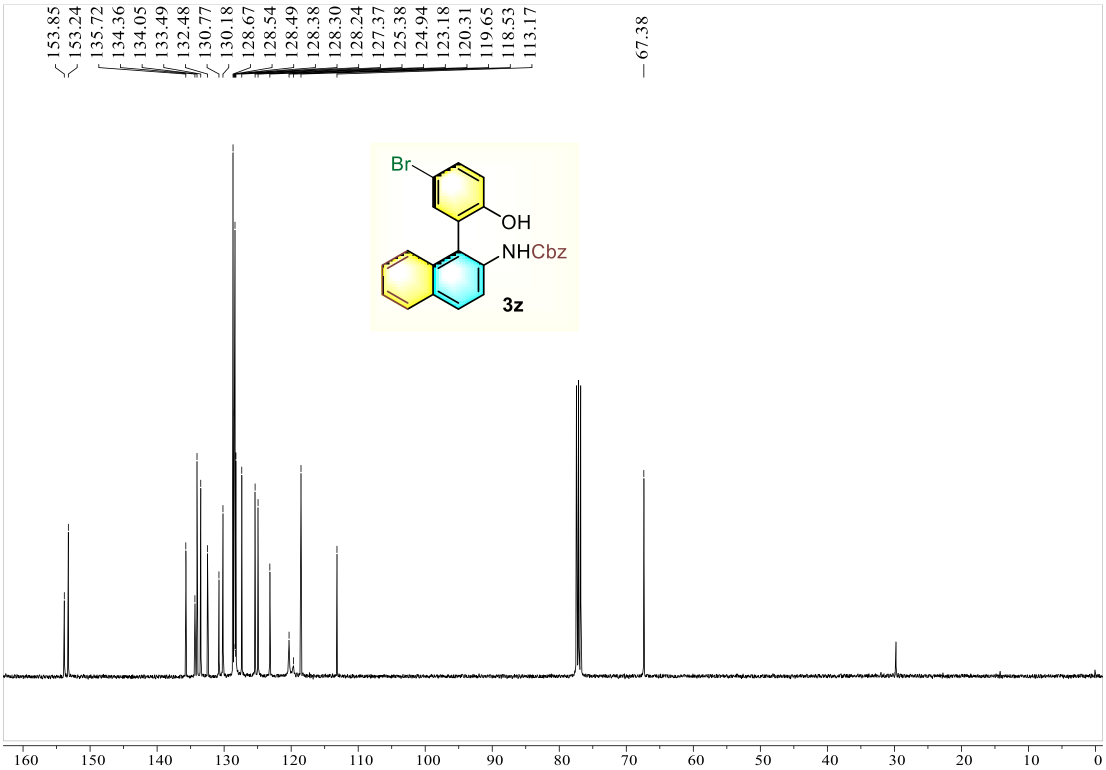
153.85
153.24
135.72
134.36
134.05
133.49
132.48
130.77
130.18
128.67
128.54
128.49
128.38
128.30
128.24
127.37
125.38
124.94
123.18
120.31
119.65
118.53
113.17
67.38
Br
OH
NHCbz
3z
160
150
140
130
120
110
100
90
80
70
60
50
40
30
20
10
0

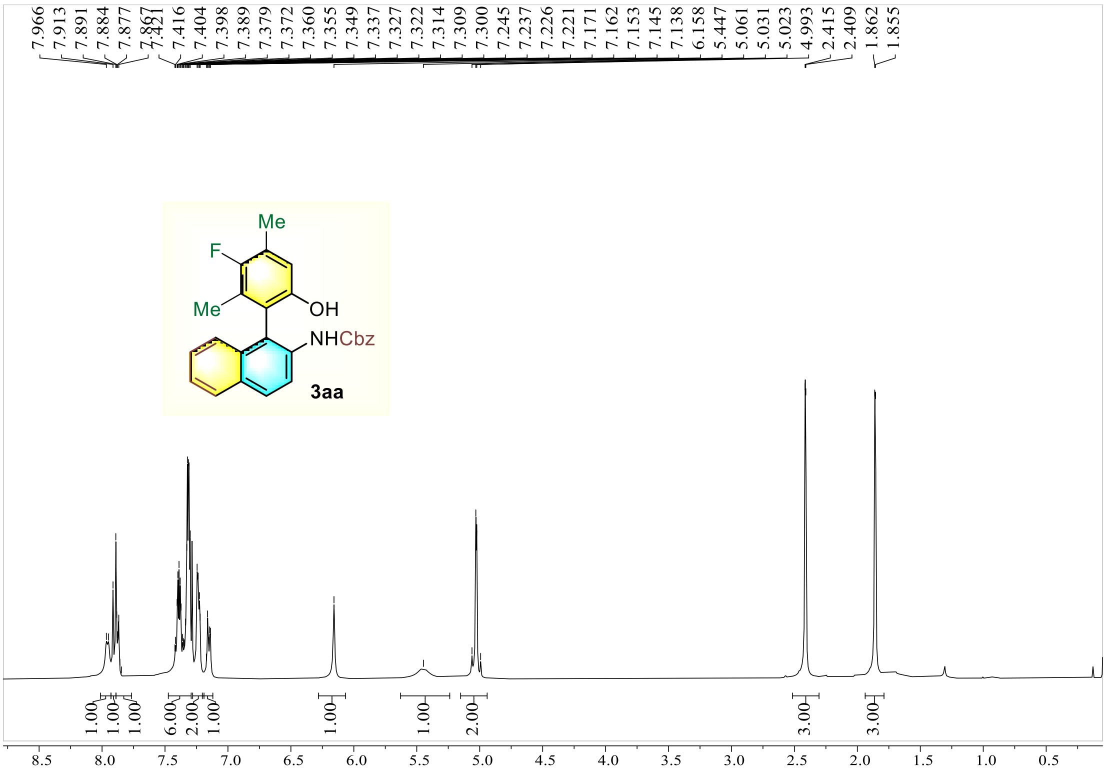
7.966
7.913
7.891
7.884
7.877
7.867
7.421
7.416
7.404
7.398
7.389
7.379
7.372
7.360
7.355
7.349
7.337
7.327
7.322
7.314
7.309
7.300
7.245
7.237
7.226
7.221
7.171
7.162
7.153
7.145
7.138
6.158
5.447
5.061
5.031
5.023
4.993
2.415
2.409
1.862
1.855
Me
F
Me
OH
NHCbz
3aa
1.00
1.00
1.00
6.00
2.00
1.00
1.00
1.00
2.00
3.00
3.00
8.5
8.0
7.5
7.0
6.5
6.0
5.5
5.0
4.5
4.0
3.5
3.0
2.5
2.0
1.5
1.0
0.5

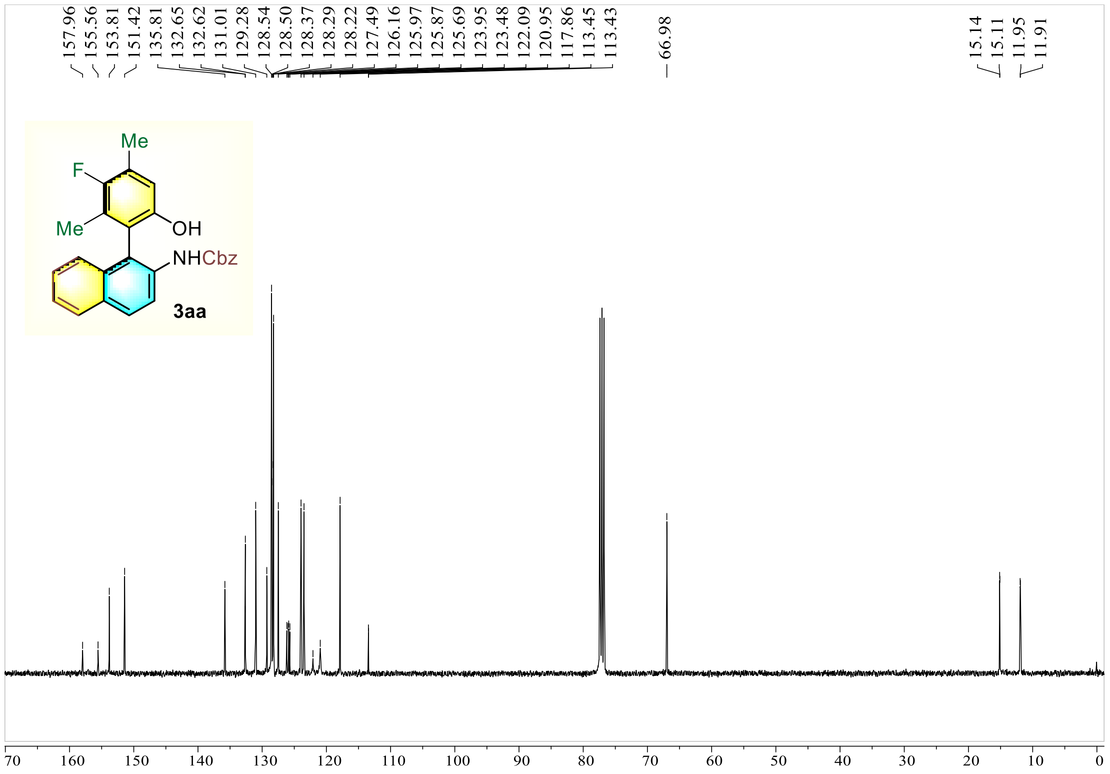
157.96
155.56
153.81
151.42
135.81
132.65
132.62
131.01
129.28
128.54
128.50
128.37
128.29
128.22
127.49
126.16
125.97
125.87
125.69
123.95
123.48
122.09
120.95
117.86
113.45
113.43
66.98
15.14
15.11
11.95
11.91
Me
F
Me
OH
NHCbz
3aa
170
160
150
140
130
120
110
100
90
80
70
60
50
40
30
20
10
0

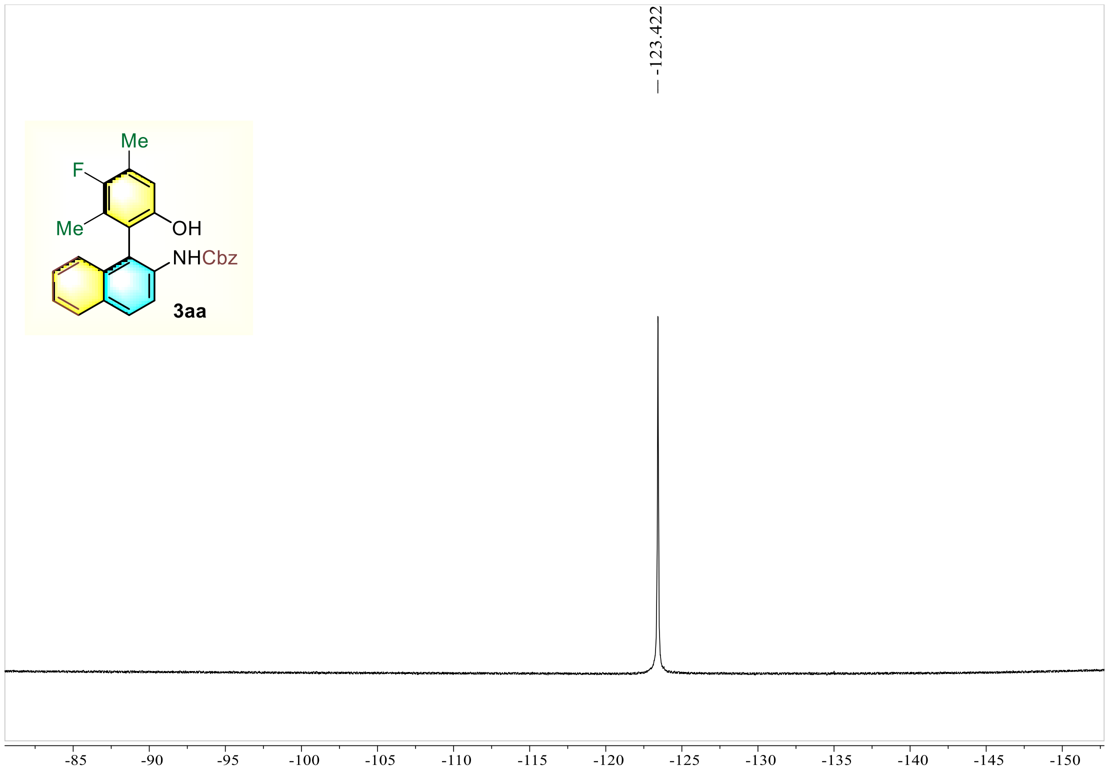
-123.422
Me
F
Me
OH
NHCbz
3aa
-85
-90
-95
-100
-105
-110
-115
-120
-125
-130
-135
-140
-145
-150

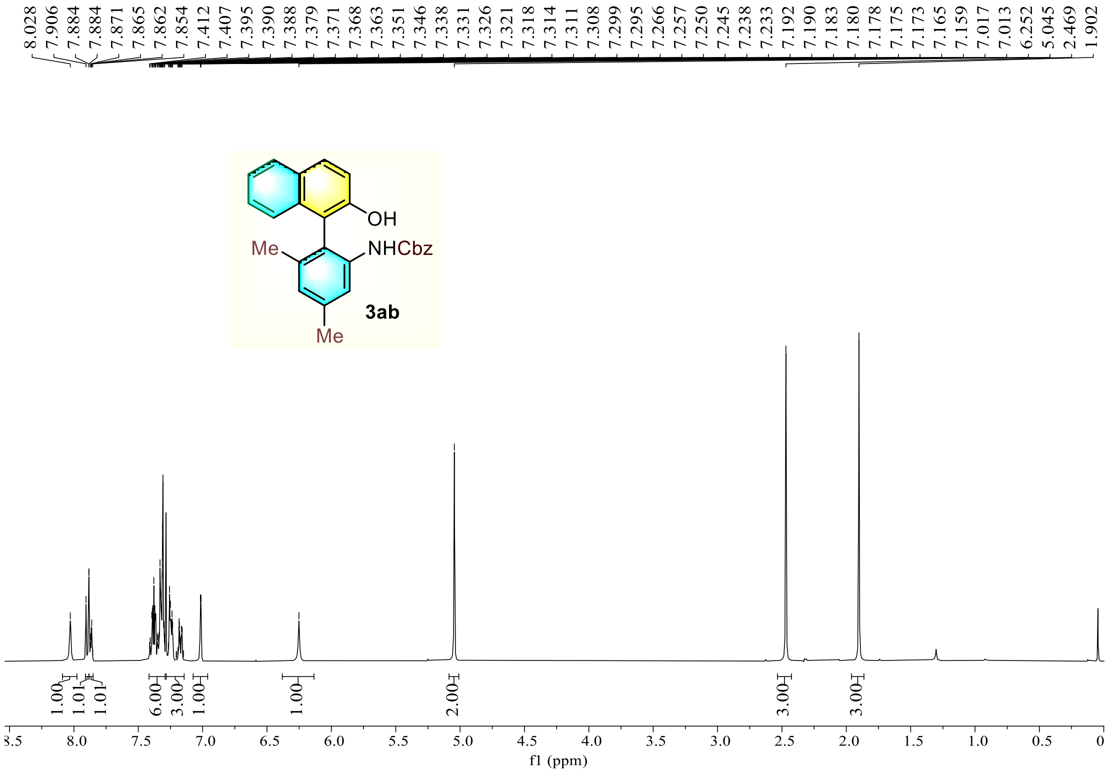
8.028 7.906 7.884 7.884 7.871 7.865 7.862 7.854 7.412 7.407 7.395 7.390 7.388 7.379 7.371 7.368 7.363 7.351 7.346 7.338 7.331 7.326 7.321 7.318 7.314 7.311 7.308 7.299 7.295 7.266 7.257 7.250 7.245 7.238 7.233 7.192 7.190 7.183 7.180 7.178 7.175 7.173 7.165 7.159 7.017 7.013 6.252 5.045 2.469 1.902
OH
Me
NHCbz
3ab
Me
1.00 1.01 1.01 6.00 3.00 1.00 1.00 2.00 3.00 3.00
f1 (ppm)

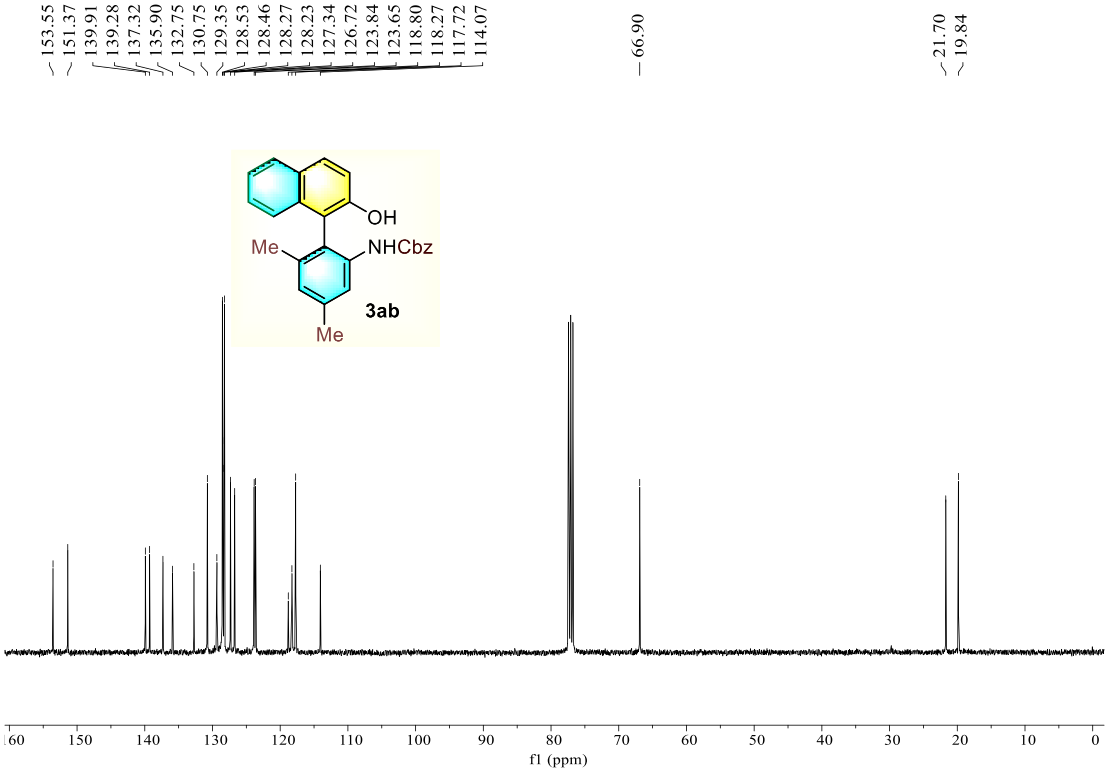
153.55 151.37 139.91 139.28 137.32 135.90 132.75 130.75 129.35 128.53 128.46 128.27 128.23 127.34 126.72 123.84 123.65 118.80 118.27 117.72 114.07
66.90
21.70 19.84
OH
Me
NHCbz
3ab
Me
f1 (ppm)

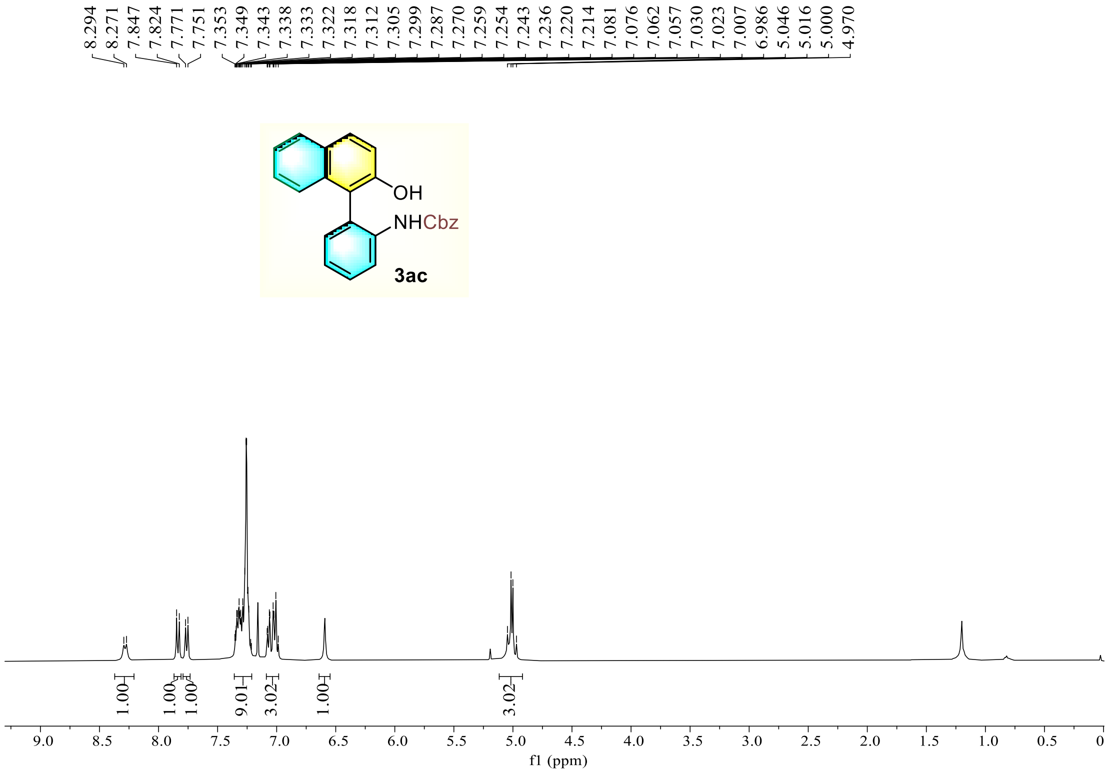
8.294
8.271
7.847
7.824
7.771
7.751
7.353
7.349
7.343
7.338
7.333
7.322
7.318
7.312
7.305
7.299
7.287
7.270
7.259
7.254
7.243
7.236
7.220
7.214
7.081
7.076
7.062
7.057
7.030
7.023
7.007
6.986
5.046
5.016
5.000
4.970
OH
NHCbz
3ac
1.00
1.00
1.00
9.01
3.02
1.00
3.02
9.0 8.5 8.0 7.5 7.0 6.5 6.0 5.5 5.0 4.5 4.0 3.5 3.0 2.5 2.0 1.5 1.0 0.5 0
f1 (ppm)

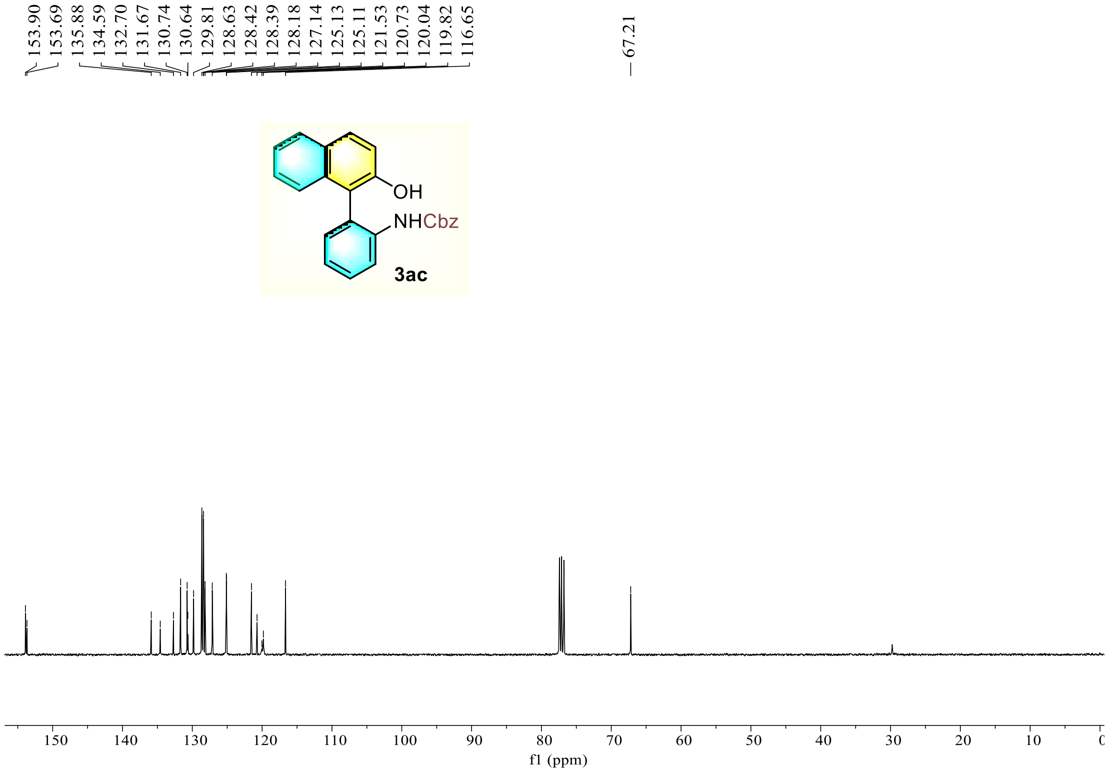
153.90
153.69
135.88
134.59
132.70
131.67
130.74
130.64
129.81
128.63
128.42
128.39
128.18
127.14
125.13
125.11
121.53
120.73
120.04
119.82
116.65
67.21
OH
NHCbz
3ac
150 140 130 120 110 100 90 80 70 60 50 40 30 20 10 0
f1 (ppm)

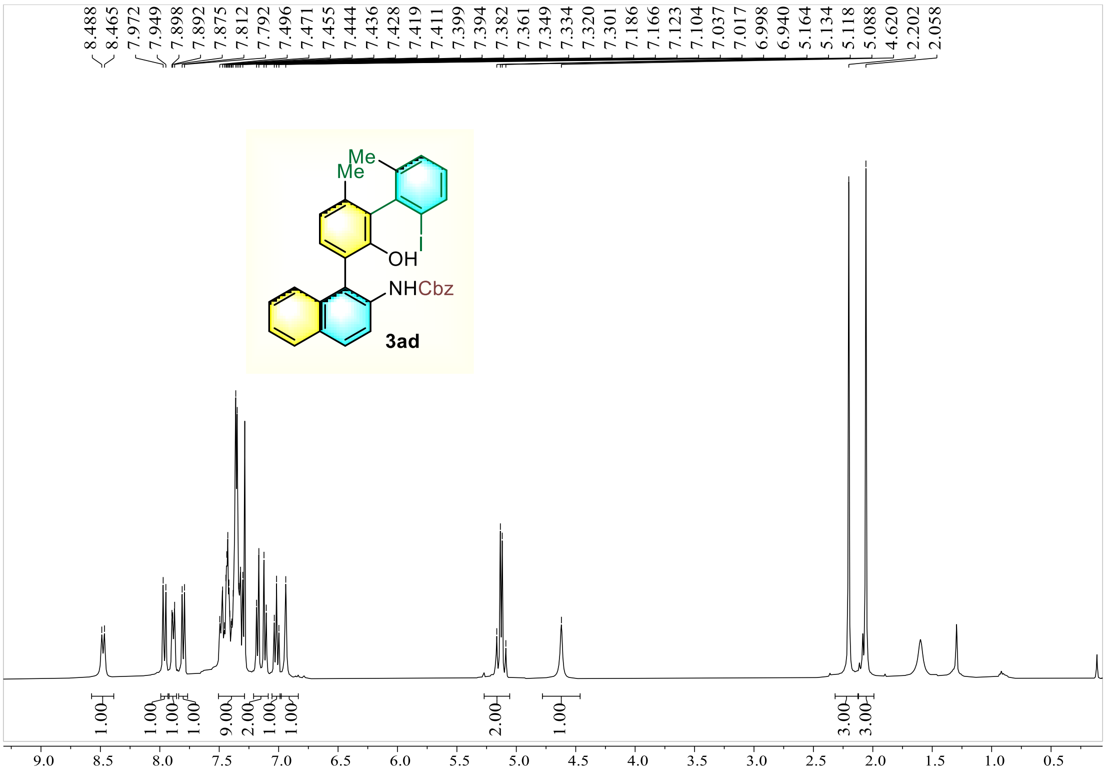
8.488
8.465
7.972
7.949
7.898
7.892
7.875
7.812
7.792
7.496
7.471
7.455
7.444
7.436
7.428
7.419
7.411
7.399
7.394
7.382
7.361
7.349
7.334
7.320
7.301
7.186
7.166
7.123
7.104
7.037
7.017
6.998
6.940
5.164
5.134
5.118
5.088
4.620
2.202
2.058
Me
Me
OH
I
NHCbz
3ad
1.00
1.00
1.00
1.00
1.00
9.00
2.00
1.00
1.00
1.00
2.00
1.00
3.00
3.00
9.0
8.5
8.0
7.5
7.0
6.5
6.0
5.5
5.0
4.5
4.0
3.5
3.0
2.5
2.0
1.5
1.0
0.5

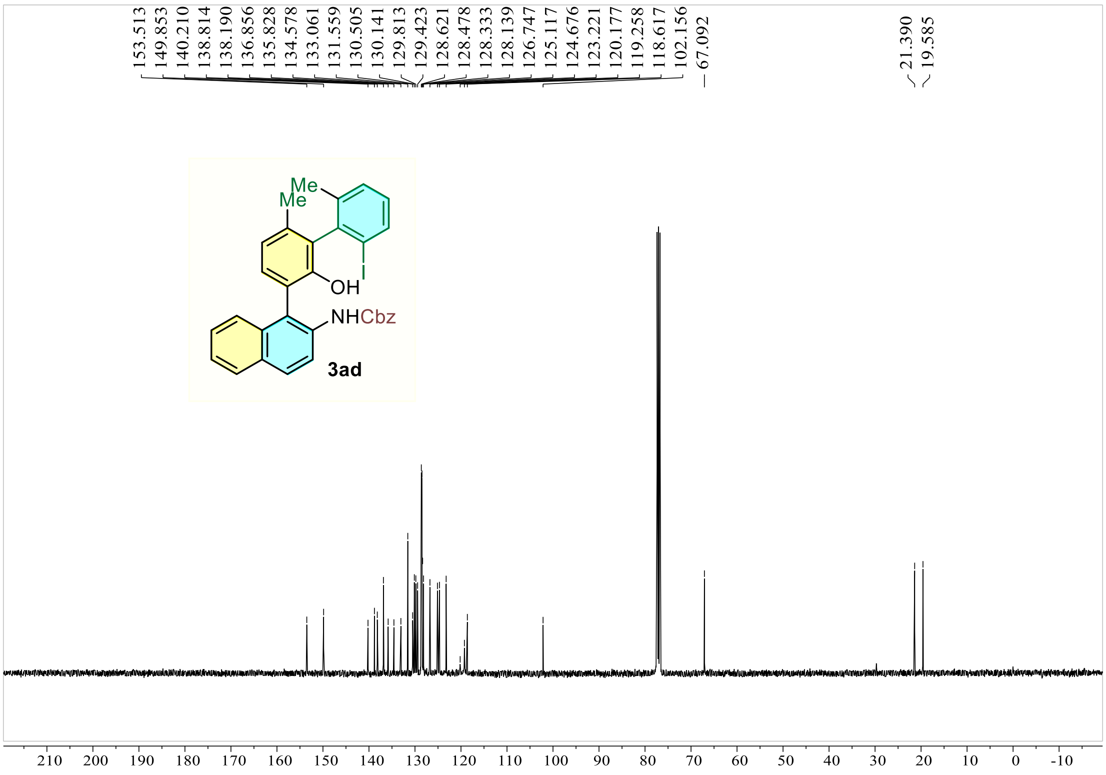
153.513
149.853
140.210
138.814
138.190
136.856
135.828
134.578
133.061
131.559
130.505
130.141
129.813
129.423
128.621
128.478
128.333
128.139
126.747
125.117
124.676
123.221
120.177
119.258
118.617
102.156
67.092
21.390
19.585
Me
Me
OH
I
NHCbz
3ad
210
200
190
180
170
160
150
140
130
120
110
100
90
80
70
60
50
40
30
20
10
0
-10

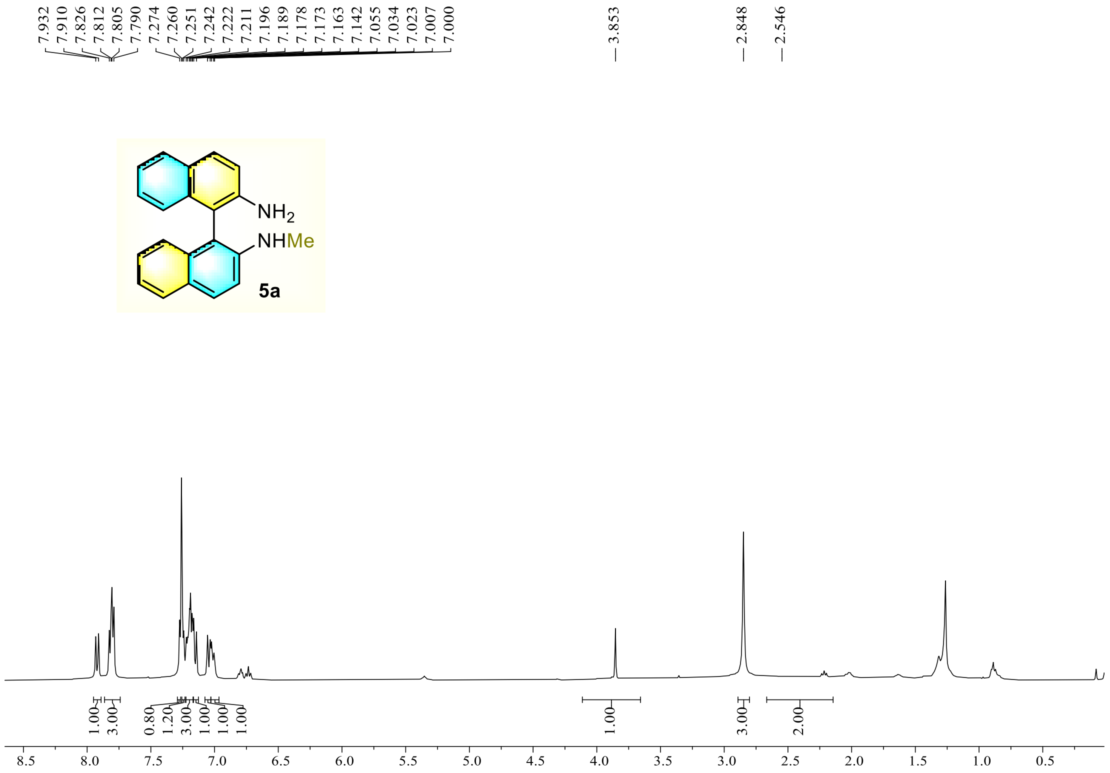
7.932
7.910
7.826
7.812
7.805
7.790
7.274
7.260
7.251
7.242
7.222
7.211
7.196
7.189
7.178
7.173
7.163
7.142
7.055
7.034
7.023
7.007
7.000
3.853
2.848
2.546
NH2
NHMe
5a
1.00
3.00
0.80
1.20
3.00
1.00
1.00
1.00
1.00
3.00
2.00
8.5 8.0 7.5 7.0 6.5 6.0 5.5 5.0 4.5 4.0 3.5 3.0 2.5 2.0 1.5 1.0 0.5

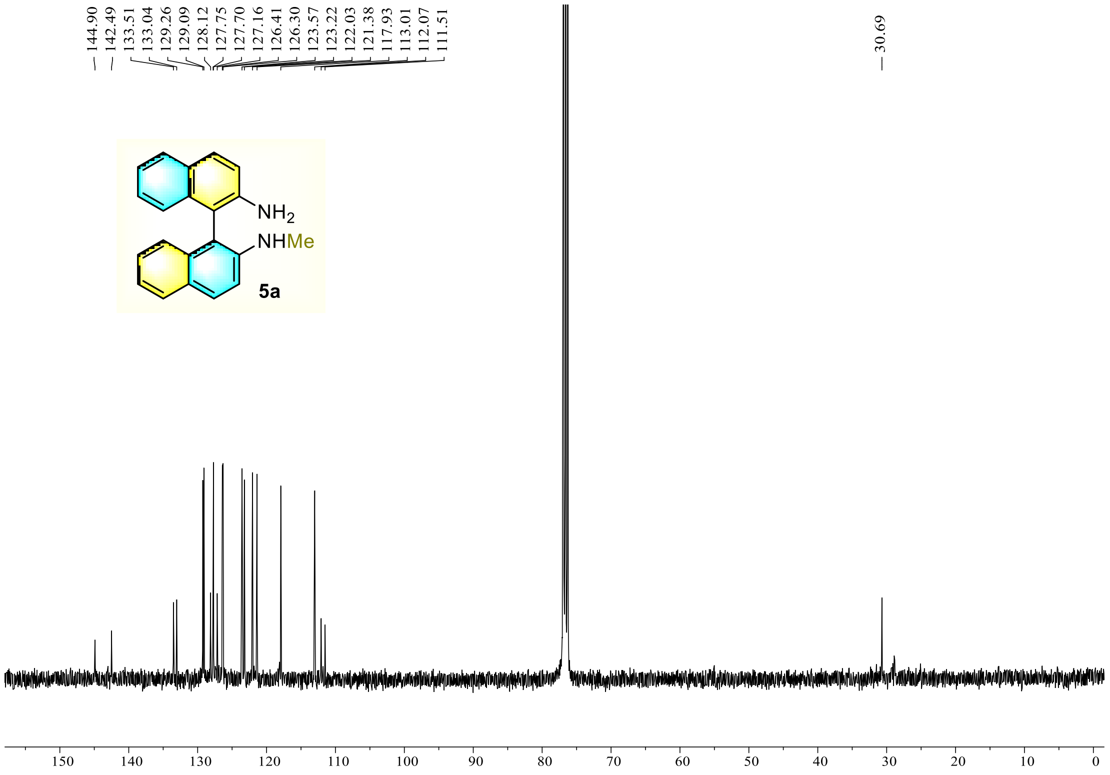
144.90
142.49
133.51
133.04
129.26
129.09
128.12
127.75
127.70
127.16
126.41
126.30
123.57
123.22
122.03
121.38
117.93
113.01
112.07
111.51
30.69
NH2
NHMe
5a
150 140 130 120 110 100 90 80 70 60 50 40 30 20 10 0

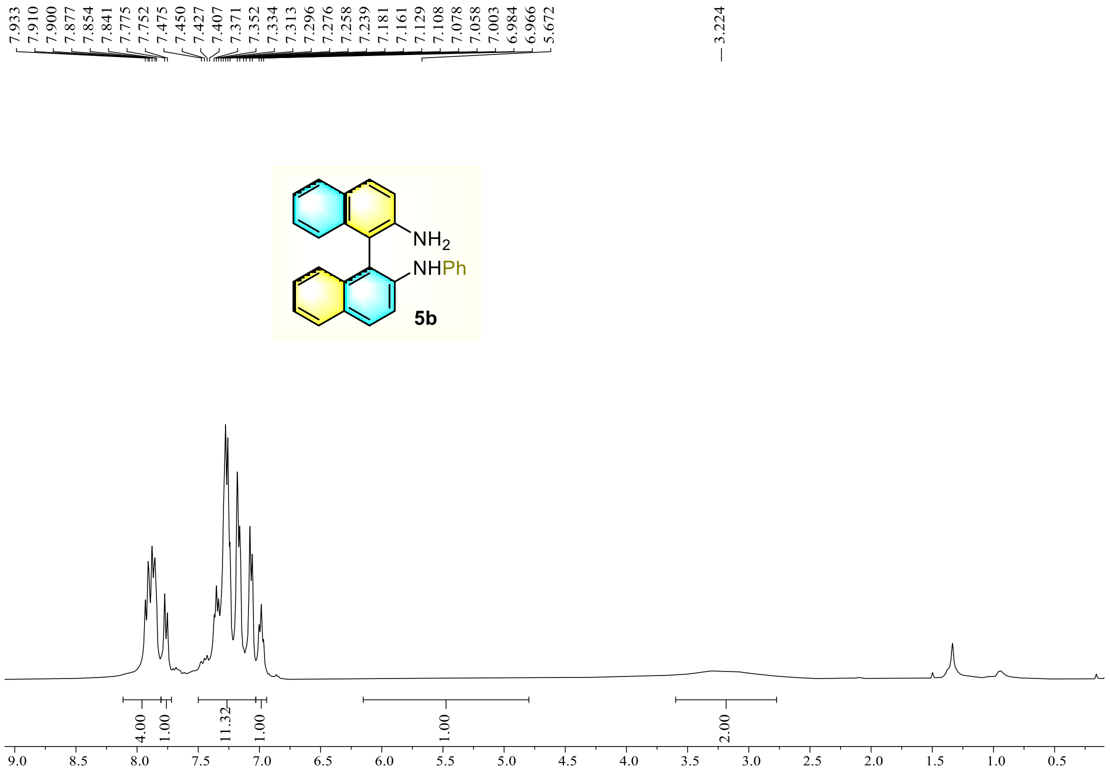

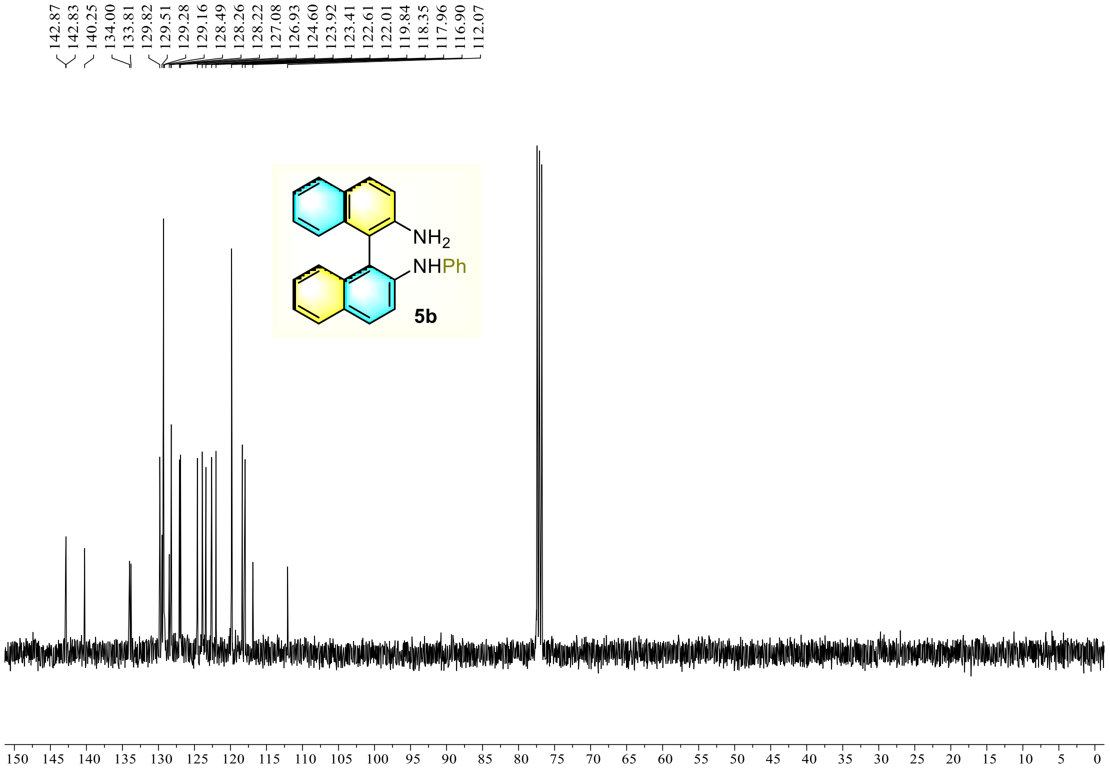

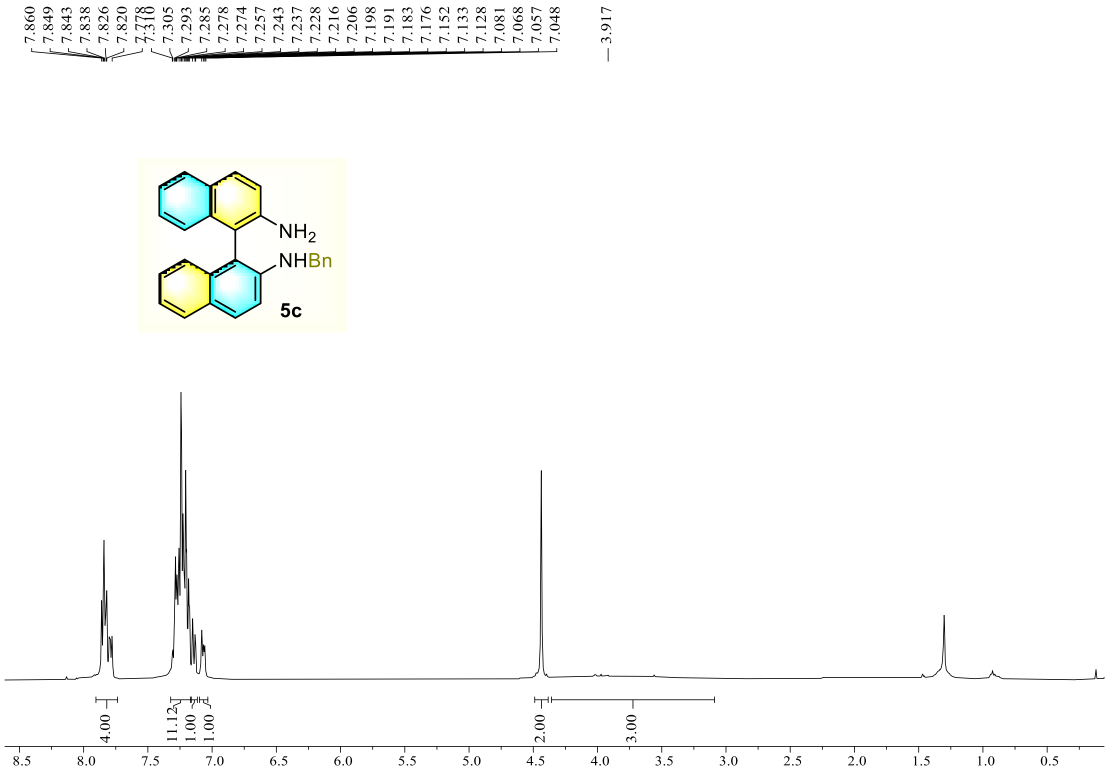
NH2
NHBn
5c

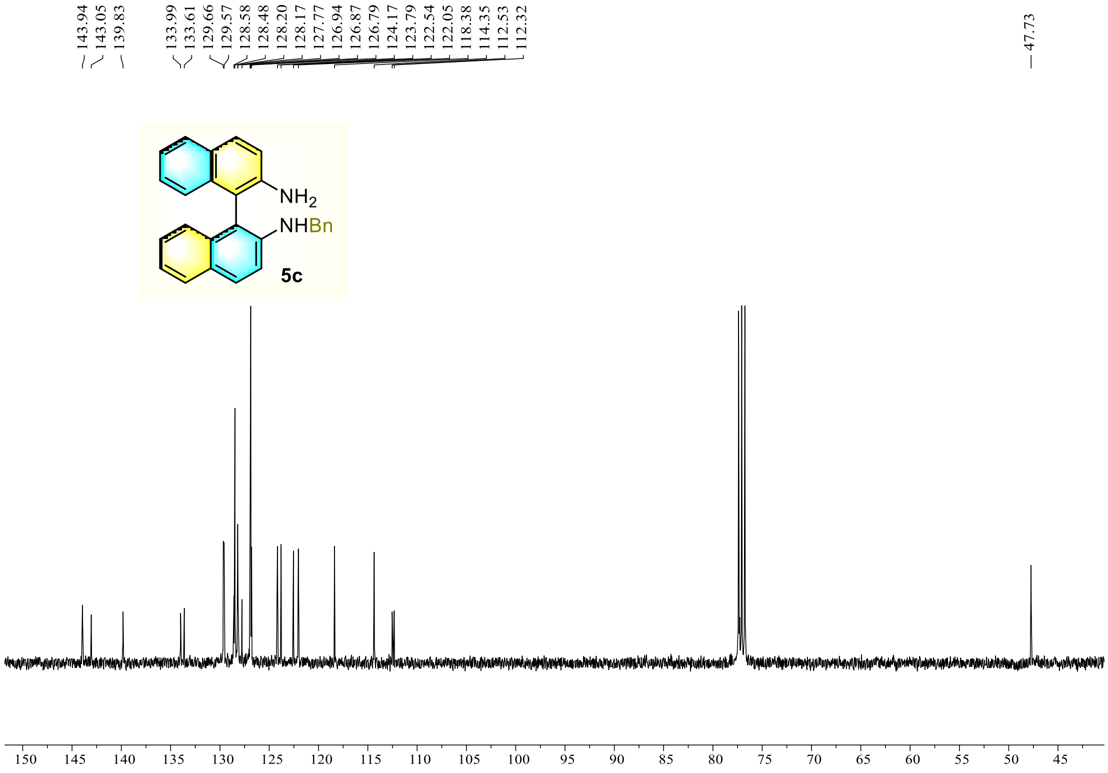
NH2
NHBn
5c

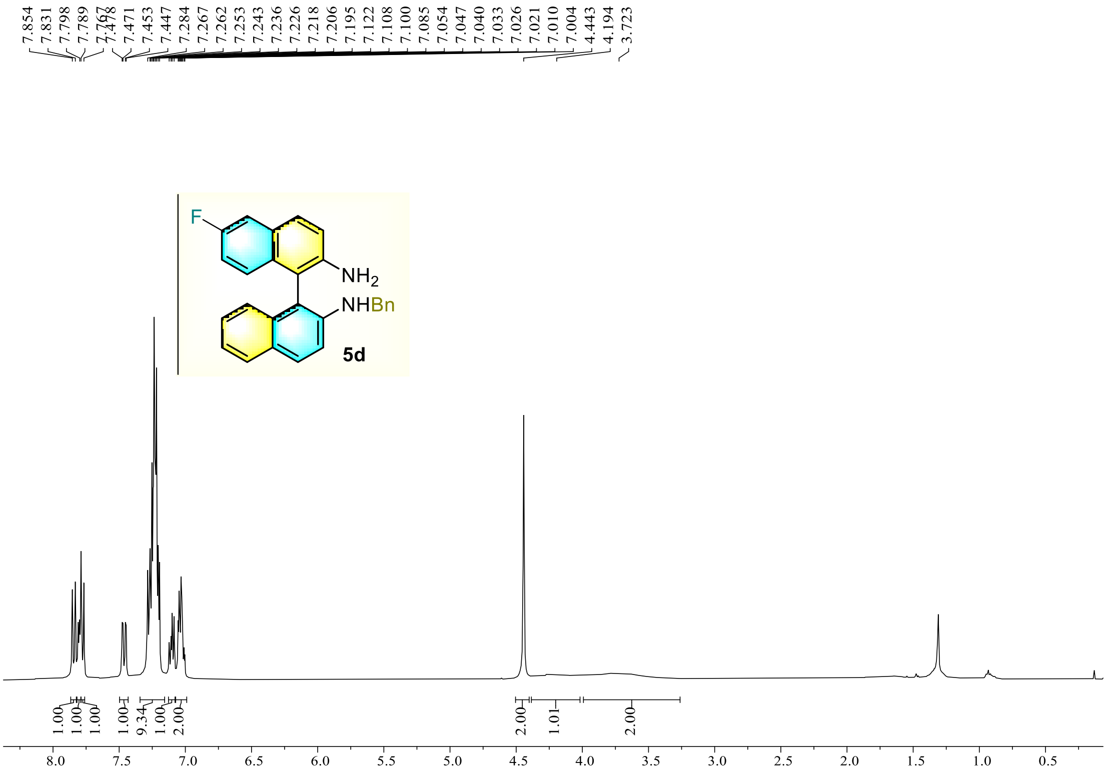

7.854
7.831
7.798
7.789
7.767
7.478
7.471
7.453
7.447
7.284
7.267
7.262
7.253
7.243
7.236
7.226
7.218
7.206
7.195
7.122
7.108
7.100
7.085
7.054
7.047
7.040
7.033
7.026
7.021
7.010
7.004
4.443
4.194
3.723
F
NH2
NHBn
5d
1.00
1.00
1.00
1.00
9.34
1.00
2.00
2.00
1.01
2.00
8.0
7.5
7.0
6.5
6.0
5.5
5.0
4.5
4.0
3.5
3.0
2.5
2.0
1.5
1.0
0.5

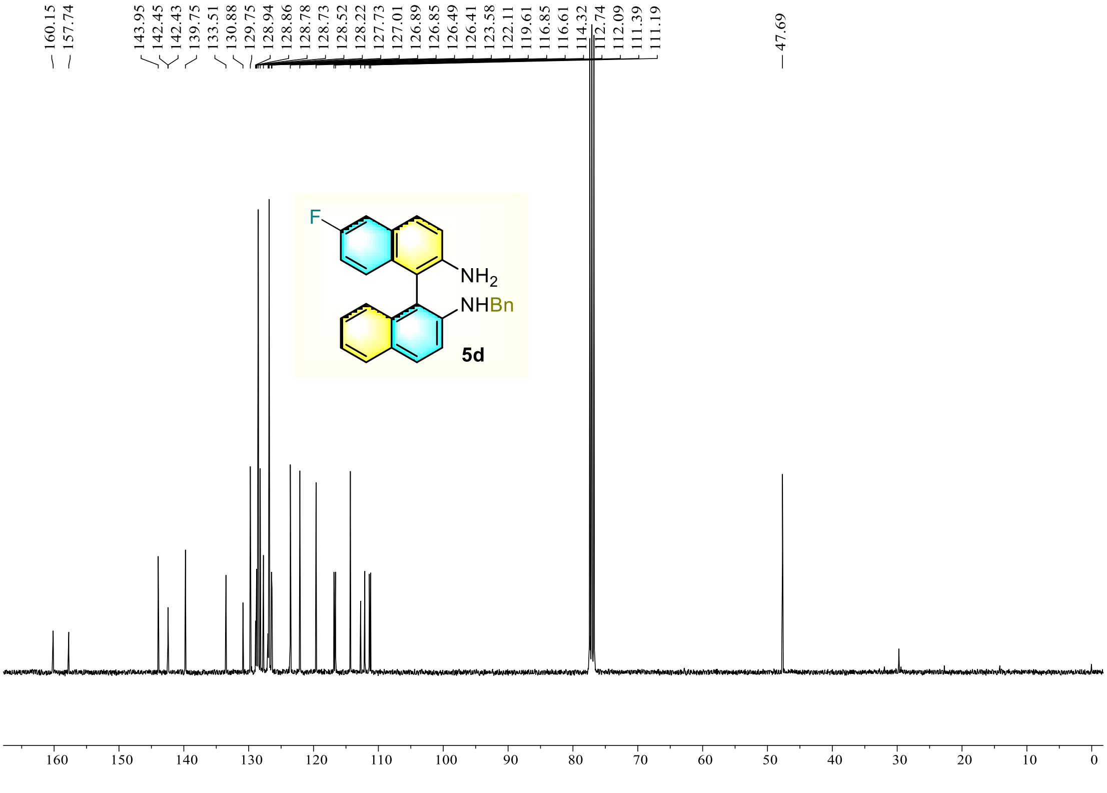

160.15
157.74
143.95
142.45
142.43
139.75
133.51
130.88
129.75
128.94
128.86
128.78
128.73
128.52
128.22
127.73
127.01
126.89
126.85
126.49
126.41
123.58
122.11
119.61
116.85
116.61
114.32
112.74
112.09
111.39
111.19
47.69
F
NH2
NHBn
5d
160
150
140
130
120
110
100
90
80
70
60
50
40
30
20
10
0

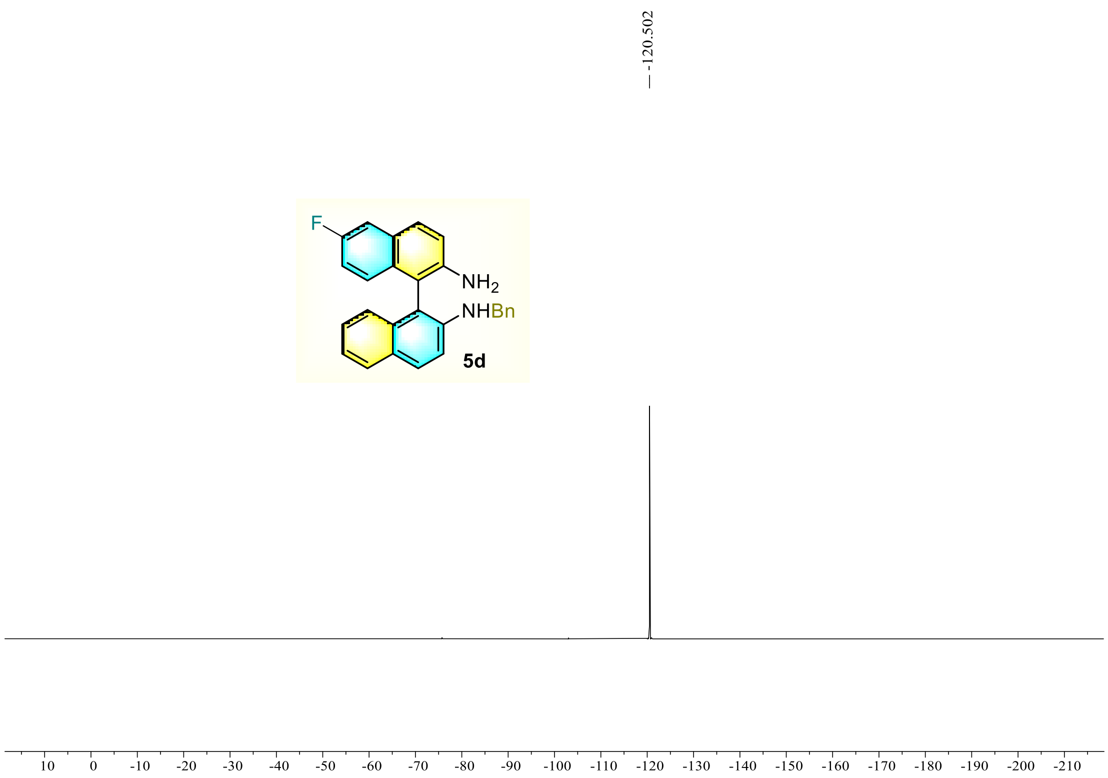
-120.502
F
NH_2
NHBn
5d
10 0 -10 -20 -30 -40 -50 -60 -70 -80 -90 -100 -110 -120 -130 -140 -150 -160 -170 -180 -190 -200 -210
